# Structural basis of the pleiotropic and specific phenotypic consequences of missense mutations in the multifunctional NAD(P)H:quinone oxidoreductase 1 and their pharmacological rescue

**DOI:** 10.1016/j.redox.2021.102112

**Published:** 2021-08-18

**Authors:** Juan Luis Pacheco-Garcia, Ernesto Anoz-Carbonell, Pavla Vankova, Adithi Kannan, Rogelio Palomino-Morales, Noel Mesa-Torres, Eduardo Salido, Petr Man, Milagros Medina, Athi N. Naganathan, Angel L. Pey

**Affiliations:** a Departamento de Química Física, Universidad de Granada, Av. Fuentenueva s/n, 18071, Granada, Spain; b Departamento de Bioquímica y Biología Molecular y Celular, Facultad de Ciencias, Instituto de Biocomputación y Física de Sistemas Complejos (GBsC-CSIC and Joint Unit), Universidad de Zaragoza, 50009, Zaragoza, Spain; c Institute of Microbiology, Academy of Sciences of the Czech Republic, Videnska 1083, Prague 4, 142 20, Czech Republic; d Department of Biochemistry, Faculty of Science, Charles University, Hlavova 2030/8, Prague 2, 128 43, Czech Republic; e Department of Biotechnology, Bhupat & Jyoti Mehta School of Biosciences, Indian Institute of Technology Madras (IITM), Chennai, 600036, India; f Departmento de Bioquímica y Biología Molecular I, Facultad de Ciencias y Centro de Investigaciones Biomédicas (CIBM), Universidad de Granada, Granada, Spain; g Center for Rare Diseases (CIBERER), Hospital Universitario de Canarias, Universidad de la Laguna, 38320, Tenerife, Spain; h Departamento de Química Física, Unidad de Excelencia en Química Aplicada a Biomedicina y Medioambiente e Instituto de Biotecnología, Universidad de Granada, Av. Fuentenueva s/n, 18071, Granada, Spain

**Keywords:** Flavoprotein, Multifunctional protein, Ligand binding, Disease-causing mutation, Post-translational modification, NQO1

## Abstract

The multifunctional nature of human flavoproteins is critically linked to their ability to populate multiple conformational states. Ligand binding, post-translational modifications and disease-associated mutations can reshape this functional landscape, although the structure-function relationships of these effects are not well understood. Herein, we characterized the structural and functional consequences of two mutations (the cancer-associated P187S and the phosphomimetic S82D) on different ligation states which are relevant to flavin binding, intracellular stability and catalysis of the disease-associated NQO1 flavoprotein. We found that these mutations affected the stability locally and their effects propagated differently through the protein structure depending both on the nature of the mutation and the ligand bound, showing directional preference from the mutated site and leading to specific phenotypic manifestations in different functional traits (FAD binding, catalysis and inhibition, intracellular stability and pharmacological response to ligands). Our study thus supports that pleitropic effects of disease-causing mutations and phosphorylation events on human flavoproteins may be caused by long-range structural propagation of stability effects to different functional sites that depend on the ligation-state and site-specific perturbations. Our approach can be of general application to investigate these pleiotropic effects at the flavoproteome scale in the absence of high-resolution structural models.

## Highlights

• Flavoprotein function is affected by ligands, mutations and posttranslational modifications.
• The structural and energetic basis of these effects are not well understood.
• We carried out extensive structure-function studies on human NQO1 WT and two mutants.
• Mutations cause long-range effects on different functional sites depending on the bound ligand.
• Phenotypic consequences are rationalized by these extensive structure-function studies.

## Graphical abstract

**Figure:**
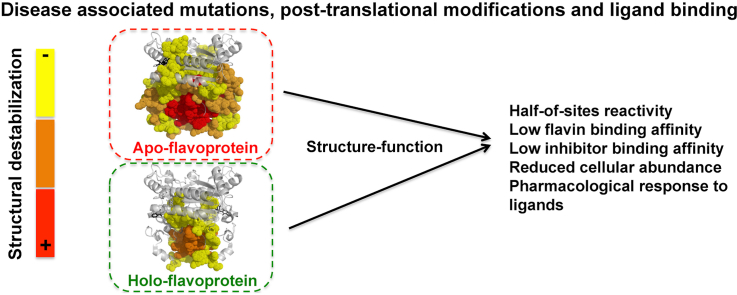

## Abbreviations

A: Arrhenius frequency factor
ASA: accessible surface area
CTD: C-terminal domain
ΔC_p_: apparent binding heat capacity
% D: percentage of deuterium incorporated
Dic: dicoumarol
DBS: dicoumarol binding site
DCPIP: 2,6-dichlorophenolindophenol
DAD: donor-acceptor-distance
DT: deuterium-transfer
E_a_: activation energy
FAD: flavin-adenine dinucleotide
FBS: FAD binding site
FMN: flavin mononucleotide
ΔΔG_prot_: change in thermodynamic stability towards proteolysis
ΔH: apparent binding enthalpy
HDX: hydrogen/deuterium exchange
HT: hydride-transfer
IPTG: isopropyl β-d-1-thiogalactopyranoside
ITC: isothermal titration calorimetry
*K*_d_: dissociation constant
*k*_HT_: limiting hydride-transfer rate constant
KIE: kinetic isotope effect
*k*_obs_: observed rate constant
*k*_prot_: second-order proteolysis rate constant
LDA model: ligand-dependent anisotropic model
LDI model: ligand-dependent isotropic model
MCI model: mutation-centered isotropic model
MMI: monomer:monomer interface
NADH: Nicotinamide adenine dinucleotide
NADD: deuterated nicotinamide adenine dinucleotide
NADPH: Nicotinamide adenine dinucleotide 2′-phosphate
N_conf_: residues folded upon ligand binding
NQO1: NADP(H):quinone oxidoreductase 1
NQO1_apo_: NQO1 with no bound ligand
NQO1_holo_: NQO1 with bound FAD
NQO1_dic_: NQO1 with bound FAD and dicoumarol
NTD: N-terminal domain
PDB: protein data bank
SDS-PAGE: polyacrylamide gel electrophoresis in the presence of SDS
Rib: riboflavin
WT: wild-type

## 1 Introduction

The human flavoproteome consist of about a hundred of different proteins [1,2] that can be regarded as *multifunctional* proteins. In a wide sense, we refer to *function* as the ability to carry out enzymatic reactions, to mediate biomacromolecular interactions and to be transported for operating in different subcellular locations, among others (Table S1). As a principle, this multifunctionality must be intrinsically linked to the ability of human flavoproteins to populate different conformational states (i.e. to their conformational lansdscape). Regarding biochemical reactions, the capacity of human flavoproteins to catalyze (mostly redox) reactions requires the presence of a bound flavin cofactor (primarily FAD and FMN) as well as the binding of other substrates and cofactors. In this sense, the diversity of biochemical reactions catalyzed by flavoproteins is remarkable (Table S1). Biomolecular association with other proteins and nucleic acids has potential regulatory effects on different cellular processes (Table S1). In addition, a large fraction of human flavoproteins operate in the mitochondria [1] and therefore, most of them must be synthesized in the cytosol and imported (i.e. through *unfolding*) to this organelle [3]. Nonetheless, human flavoproteins display a substantial diversity of subcellular locations (about two-thirds show more than one cellular location; Table S1), and upon cytosolic synthesis must fulfill different requirements for translocation through dedicated macromolecular machineries to different subcellular locations such as the nucleus, peroxisomes or endoplasmic reticulum among others [[4], [5], [6]]. Flavin binding is also required to modulate their enzyme activity, intracellular stability and protein:protein interactions [7,8]. In addition, the redox status of the flavin is not only required for their enzymatic function, but can also modulate others features such as subcellular location [9]. Interestingly, although many high-resolution structures have been described for human flavoproteins with different bound ligands (i.e. ligation states), there is virtually no such information on their flavin-free apo-state (Table S1). This is an important issue, since the structure and energetics of the apo-state can contribute to different functions, such as flavin binding affinity, intracellular stability, biomacromolecular interactions and subcellular transport.

Due to their multiple functions, a large fraction of human flavoproteins has been associated with inherited diseases through loss-of-function mutations [2]. In addition, a vast number of potential post-translational modifications (mostly phosphorylation events) have been reported by high-throughput techniques, although the site-specific functional consequences of these events are largely unknown [10]. Considering the evidence on the multifunctional nature of human flavoproteins, it is plausible that missense mutations and post-translational modifications will affect to different extents certain of these multiple functional features [[11], [12], [13], [14]]. Indeed, structure-based stability analyses support that disease-associated mutations and phosphorylation may share a similar destabilizing effect on the structure of human flavoproteins, particularly when such events affect residues buried within the protein interior (Figure S1 and Table S2).

**Fig. 1:**
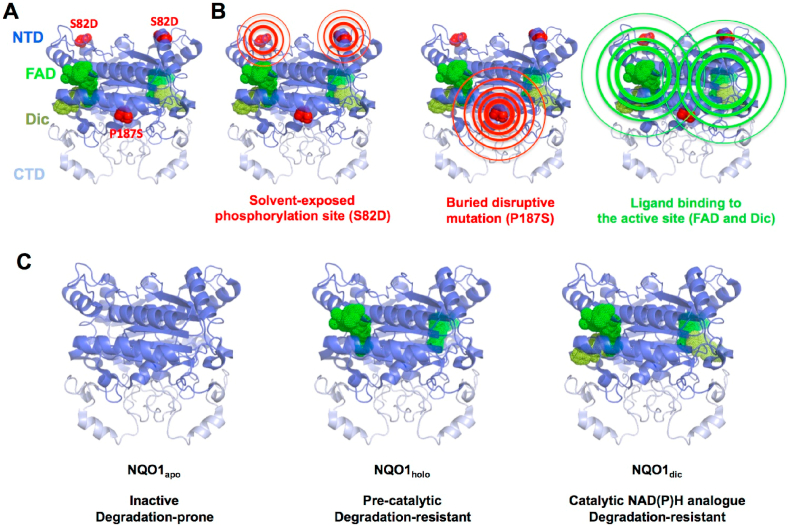
**Structural features of the NQO1 protein dimer and the sites perturbed in this work**. A) Crystallographic model of the NQO1 dimer (PDB code 2F1O [25]) showing its two-domain structure (NTD, N-terminal domain and CTD, C-terminal domain) and the location of the S82D and P187S sites as well as FAD and Dic binding sites. Panel B shows the potential propagation of the *allosteric perturbation events* studied here (S82D, P187S, FAD and Dic binding). C) Relevant ligation states investigated in this work show different catalytic and stability features in WT NQO1.

To study the *allosteric* interplay between ligand binding, post-translational modifications and disease-associated mutational effects, we have investigated here the multifunctional human protein NAD(P)H:quinone oxidoreductase 1 (NQO1) [15]. Alterations in NQO1 functionality are associated with a variety of diseases, including cancer, Parkinson's and Alzheimer diseases, diabetes, multiple sclerosis, schizophrenia, metabolic syndrome and benzene toxicity [15,16]. NQO1 is a flavoprotein that catalyzes the two-electron reduction of a wide variety of natural and synthetic quinones (in the presence of NAD(P)H as coenzyme) and interacts with and stabilizes transcription factors critical for cancer development (such as p53, p73α and HIF-1α) [[15], [16], [17], [18]]. NQO1 is primarily cytosolic, although it can also be found in mitochondria, nucleus and perinuclear regions (Table S1) [9]. NQO1 forms functional homodimers with a molecular size of 62 kDa and containing two functional domains within each monomer (Fig. 1A) [[19], [20], [21]]. The N-terminal domain (NTD, residues 1–225) is capable of forming stable dimers that bind FAD, although the presence of the C-terminal domain (CTD) is required to complete the active site [21,22]. NQO1 is a remarkable example of human flavoprotein that exists in different conformational (i.e. ligation-dependent) states with very different functionalities [[7], [8], [9],^12^,^15^,^16^,^18^,^23^]. In the absence of FAD (the NQO1_apo_ state) the WT protein shows a remarkably low conformational stability, with a minimal stable core that holds the protein dimer and the FAD and NAD(P)H binding sites populating non-competent binding conformations [23]. The NQO1_apo_ state is rapidly degraded inside the cell by ubiquitin-dependent proteasomal activity and display altered interactomic patterns [7,8]. Consequently, FAD binding (forming the NQO1_holo_ state) triggers a population-shift in the conformational ensemble (i.e. a conformational change) leading to a precatalytic competent state in which the FAD and substrate/NADP(H) binding sites are stabilized, providing a boost in intracellular stability through stabilization of the CTD [7,12,15]. Binding of NAD(P)H is the rate-limiting step in the reductive half-reaction of NQO1 [22,24], and this state with the flavin reduced is associated with binding to microtubules and α-tubulin acetylation [9], and more generally, it promotes the interaction of NQO1 with protein partners [16,18]. Binding of dicoumarol (Dic, forming the state NQO1_dic_), a competitive inhibitor of NAD(P)H that may resemble a transition state analogue in both reductive and oxidative half-reactions, also lead to significant changes in the local stability in the protein ensemble without causing gross conformational changes [[23], [24], [25]]. Binding of this inhibitor seems to prevent the interaction of NQO1 with other proteins [18].

Several single amino acid substitutions in NQO1 have been shown to affect different functional sites (and features) of NQO1, sometimes located far from the mutated site [8,[10], [11], [12], [13], [14],^26^,^27^]. The common cancer-associated polymorphism Pro187Ser/P187S [16,28] is known to severely affect FAD binding and to reduce the intracellular stability of the protein, affecting also the interactomics of the protein [7,8,12,15,21,22,26]. This polymorphism affects a residue that is fully buried in the structure (% ASA strictly 0; using PDB code 2F1O and the software GetArea; http://curie.utmb.edu/getarea.html; average of eight monomers) and close to the dimer interface (Fig. 1B). Therefore, we may expect that its local structural and energetic effects will efficiently propagate across the entire structure of the NQO1 dimer (likely amplified through some *constructive interference* between the two mutated sites in the homodimer) (Fig. 1). The phosphomimetic mutation Ser82Asp/S82D, affects a partially exposed site (% ASA of 16 ± 8%; using the same procedure) (Fig. 1A) and seems to cause more local effects, causing a 30-fold decrease in binding affinity for FAD [10](Fig. 1). However, this decrease in affinity and/or alterations in structural stability may lead to a concomitant decrease in intracellular stability [10]. Here, we have used these two mutations to unravel how different type of mutations may lead to different phenotypic consequence in a multifunctional flavoprotein, and how these effects are dependent on bound ligands (Fig. 1). To this end, we carried out extensive structure-function analysis using a wide array of computational, biochemical, biophysical, cell and structural biology approaches.

## 2 Materials and methods

### 2.1 Protein expression and purification

Expression and purification of NQO1 WT, S82D and P187S were carried out as described [23,24]. *E. coli* BL21 (DE3) cells were transformed with the pET46 Ek/LIC vector containing the cDNA of NQO1 WT, S82D or P187S [10,11]. A preculture of single clones was grown in 400 mL LBA medium (LB containing 0.1 mg·ml^−1^ ampicillin) for 16–20 h at 37 °C. Cells were harvested and resuspended in 4.8 L of LBA and grown at 37 °C for 3 h and NQO1 expression was induced by the addition of 0.5 mM IPTG (isopropyl β-d-1-thiogalactopyranoside) and lasted for 6 h at 25 °C. Induced cells were harvested by centrifugation and frozen overnight at −80 °C. NQO1 proteins were purified using immobilized nickel affinity chromatography columns (GE Healthcare) and size-exclusion chromatography as described [24]. Isolated dimeric fractions of NQO1 variants were buffer exchanged to K-HEPES buffer 50 mM pH 7.4 using PD-10 columns (GE Healthcare). The UV–visible spectra of purified NQO1 proteins were registered in a HP8453 UV–Visible spectrophotometer (Agilent Technologies, Waldbronn, Germany) and used to quantify the content of FAD as described [24]. Prior to pre-steady state kinetic analyses, proteins were incubated with 1 mM FAD and the excess of FAD removed using PD-10 columns, obtaining a saturation fraction (FAD:NQO1 monomer) higher than 95% based on UV–visible spectra. Apo-proteins were obtained by treatment with 2 M urea and 2 M KBr as described [23], obtaining samples with less than 2% saturation fraction of FAD based on UV–visible spectra. Samples were stored at −80 °C upon flash freezing in liquid N_2_. Protein purity and integrity was checked by polyacrylamide gel electrophoresis in the presence of sodium dodecylsulphate (SDS-PAGE) and intact mass analysis using direct infusion on 15T ESI-FT-ICR MS as described previously [23].

### 2.2 Hydrogen-deuterium exchange (HDX) monitored by mass spectrometry

Backbone amide hydrogen/deuterium exchange (HDX) was monitored for NQO1_apo_, NQO1_holo_ and NQO1_dic_ forms of NQO1 using mass spectrometry. Data were collected for S82D and P187S variants whereas data for WT are those previously published [23]. It should be noted that all the data were acquired within one set of experiments which ensures their full comparability and diminishes possible variations eventually arising due to different experimental settings. Therefore all procedures including sample preparation, sample measurement and data processing were identical and performed as described previously [23]. To generate NQO1_holo_ and NQO1_dic_ states, 20 μM purified NQO1 protein was preincubated with 10-molar excess of FAD (NQO1_holo_) for 5 min. Generation of NQO1_dic_ required an additional 5 min incubation with 10-molar excess of dicoumarol. Isotope exchange reaction was started by 10-fold dilution of the protein solution into D_2_O-based 50 mM K-HEPES, pD 7.4, 1 mM TCEP (tris(2-carboxyethyl)phosphine). The exchange was followed for 10 s, 30 s, 2 min, 5 min, 20 min, 1 h and 3 h. The reaction was quenched by the addition of 0.5 M Glycine-HCl, pH 2.3 in a 1:1 ratio and flash frozen in liquid N_2_. The 10 s, 5 min and 3 h aliquots were replicated three times. LC-MS analysis was started by rapid thawing of the sample and injection onto the custom-made nepenthesin-2 (Nep2) and pepsin columns coupled in tandem (each of them having a bed volume 66 μL). Generated peptides were trapped and desalted on a VanGuard Pre-column (ACQUITY UPLC BEH C18, 130 Å, 1.7 μm, 2.1 mm × 5 mm, Waters, Milford, MA, USA). The digestion and desalting (total time 3 min) were driven by 0.4% formic acid (FA) in water pumped by 1260 Infinity II Quaternary pump (Agilent Technologies, Waldbronn, Germany) at a flow rate of 200 μL min^−1^. Desalted peptides were then separated on an analytical column (ACQUITY UPLC BEH C18, 130 Å, 1.7 μm, 1 mm × 100 mm, Waters, Milford, MA, USA) using linear gradient (5%–45% B in 7 min) followed by a quick step to 99% B lasting 5 min, where the solvent A was 0.1% FA/2% acetonitrile (ACN) in water, B was 0.1% FA/98% ACN in water. The gradient was delivered by the 1290 Infinity II LC System (Agilent Technologies, Waldbronn, Germany) at a flow rate 40 μL ·min^−1^. The analytical column was coupled to an ESI source of 15T FT-ICR mass spectrometer (solarix XR, Bruker Daltonics, Bremen, Germany) operating in broad-band MS mode. Acquired data were exported using DataAnalysis v. 5.0 (Bruker Daltonics, Bremen, Germany), processed by in-house designed software program Deutex (unpublished) and handled as described previously [23]. Peptide identification was performed using separate LC-MS/MS analyses followed by MASCOT database searching (version 2.4, Matrix Science, London, United Kingdom) against a custom-built database containing sequences of the proteases and the NQO1 variants under investigation. Fully deuterated controls were prepared as described previously [23] and correction for back-exchange was applied according to Ref. [29]. HDX-MS related data, graphs and images in the Supplementary material include sequence coverage map (plotted with MSTools - http://peterslab.org/MSTools/), all deuterium uptake plots for apo, holo and dicoumarol states of wild-type, S82D and P187S NQO1 variants, selected examples of isotopic profiles (all time points plus non- and fully-deuterated samples) for NQO1_apo_ and tables (Tables S3-S4) summarizing digestion metrics and a full HDX dataset (averaged values - %D - after back-exchange correction). HDX results are presented in two different ways: i) %D at a given time, that just reflects the % of total H exchanged for a given protein segment; ii) Δ%D_av_, a single metric that compares the kinetic behavior of a given segment between two variants in a given ligation state (i.e. S82D_apo_ vs. WT_apo_) as the average of the three time points that show maximal difference in % D along a time course. This metric provides a simple way to identify those segments with different stabilities between two protein variants in a given ligation state as well as a semiquantitative ranking of these differences [23].

### 2.3 Structure-based models for the propagation of mutational and ligand binding effects from HDX

The native conformational landscape of NQO1_apo_ was generated from the Ising-like and native-centric WSME (Wako-Saitô-Muñoz-Eaton) model [30,31]. This employs a binary treatment of folding wherein folded- and unfolded-like residues are assigned the binary variables *1* and *0*, respectively. We used a version that allows for only a single stretch of folded residues (single sequence approximation, SSA), two stretches folded residues (double sequence approximation, DSA) and DSA with interaction across the folded islands despite allowing for the intervening residues to be unfolded (DSAw/L), to generate the conformational landscape of NQO1. Moreover, instead of considering every residue as a folding unit that results in >400 million microstates, we employ an approximation where consecutive residues are treated as blocks (in this case, a block length of 5 is considered) [32]. This reduces the total number of microstates to 1,443,586 making it computationally less intensive. The model includes contributions from van der Waals interactions (identified with a 6 Å cutoff excluding nearest neighbors), electrostatics (via a Debye-Hückel formalism), simplified solvation and sequence-structure dependent conformational entropy [33]. We employ a mean-field van der Waals interaction energy of −49 J mol^−1^, entropic penalty for fixing a residue in the native conformation of −14 J mol^−1^ K^−1^ per residue, heat capacity change on forming a native contact of −0.36 J mol^−1^ K^−1^ per native contact and an excess conformational entropy (for disordered residues and glycine) of −6.1 J mol^−1^ K^−1^ per residue. Free energy surfaces were generated by accumulating partial partition functions with fixed number of folded blocks or their combination.

First, we applied a *mutation-centered-isotropic model* (MCI model). Briefly, the effects of mutations on the local stability were derived from analysis of HDX data by calculating Δ%D_av_ values for different segments between a mutant and the WT protein for each ligation state (NQO1_apo_, NQO1_holo_ and NQO1_dic_). Then, the distance between the mutated site (residue 82 or 187) and any given protein segment derived from HDX data, was calculated as the minimal distance (d) between the C_α_ atoms in the center of the segments corresponding to the mutated site and the rest of (104) segments analyzed experimentally. Therefore, the reference system for these calculations is the same for all ligation states (i.e. the mutated site). For these calculations, we used the structure of dimeric NQO1 in the NQO1_dic_ state (PDB: 2F1O). The results (Δ%D_av_ vs. distance, d) were eventually clustered in four groups according to their distance to the mutated site: < 10 Å, 10–20 Å, 20–30 Å and >30 Å. Data pairs (Δ%D_av_ vs. distance) corresponding to each group were averaged (±s.d.) and fitted to the following equation (Equation (1)):

(Eq 1) $$A\left(d\right)=A_{0}\cdot e^{\left(-\frac{d}{d_{c}}\right)}$$

where A(d) is the Δ%D_av_ at a given d (for each of the four groups), A_0_ is the Δ%D_av_ at d = 0 (i.e. at the mutated site) and d_c_ is a characteristic distance value for the propagation of stability effects.

In a second approach, we used reference systems that changed according to the ligation state, while keeping the dependent variable (i.e. Δ%D_av_) as it was described in the MCI model. Briefly, the reference systems were: i) NQO1_apo_.- the reference system was the Cα of the mutated site; ii) NQO1_holo_ and NQO1_dic_.- the reference system was the center of mass of the ligand (i.e. FAD and Dic, respectively). In all cases, the centroid of the Cα atoms of the experimentally determined segments were used to calculate distances from the corresponding reference point. This approach was implemented in two different ways. In the first one, the pairs of Δ%D_av_ and d values were calculated and clustered in four groups as described in the MCI model. This way was termed as *ligand-dependent-isotropic model* (LDI model). In a second one, we applied a zone method in which the 3D space was divided into 6 zones (angle of 60° each) using 3 planes at an angle of 120° to each. Zone 1 contains the Cα – Cβ bond of the mutated residue in case of the NQO1_apo_ and the imaginary line connecting the center of masses of the ligand and its interacting residues in case of NQO1_holo_ and NQO1_dic_. Zones 2 and 6 are adjacent to Zone 1 and together account for residues in the direction of perturbation. Zones 4, 5 and 3 fall exactly opposite to zones 1, 2 and 6 respectively and are structurally opposite to the direction of perturbation. Residues inside each zone were clustered into 4 groups as abovementioned and averaged (±s.d.). We refer to this method as *ligand-dependent-anisotropic model* (LDA model).

### 2.4 Pre-steady state enzyme kinetic analysis

Fast hydride-(HT) or deuteride-(DT) transfer reactions were carried out under anaerobic conditions using a stopped-flow spectrophotometer (SX.18 MV, Applied Photophysics Ltd.) interfaced with a photodiode array detector, essentially as described [24]. Briefly, the reductive half-reaction was measured by mixing NQO1_holo_ variants (7.5 μM) with either NADH ranging from 7.5 to 100 μM or with NADPH or 4*R*-^2^H-NADH (NADD) at stoichiometric concentrations (7.5 μM). To study the oxidative half-reaction, 7.5 μM NQO1_hq_ (formed by reaction of NQO1_holo_ with stoichiometric amounts of NADH) was mixed with 7.5 μM of 2,6-Dichlorophenolindophenol (DCPIP). Reactions were performed in 20 mM HEPES-KOH, pH 7.4. Multiple wavelength absorption data in the flavin absorption region were collected and processed as described [24]. Time-dependent spectral deconvolution was performed by global analysis and numerical integration methods using previously described procedures [24]. Basically, this deconvolution procedure was carried out considering sequential and irreversible steps in the context of two (A→B→C) or three (A→B→C→D) step mechanisms, where A-D are spectral species (not necessarily a given *state*), and allowed to determine observed rate constants (*k*_obs_) for these steps as well as spectroscopic properties of A and D are initial and final, but not intermediate states A to D species. According to a recent study, catalytically relevant processes involved steps A→B→C [24]. Hyperbolic dependences of *k*_obs_ vs. NADH concentrations were fitted using equation (2):

(Eq 2) $$k_{obs}=\frac{k_{HT}\cdot \left[NADH\right]}{K_{d}^{NADH}+\left[NADH\right]}$$

where *k*_HT_ is the limiting rate constant for HT and K_d_^NADH^ is the equilibrium dissociation constant to a given active site.

For estimation of primary kinetic isotopic effects (KIEs) in the HT process [34], HT or DT *k*_obs_ from NADH/D to NQO1_holo_ were evaluated at different temperatures in samples containing equimolecular mixtures of protein and coenzyme (7.5 μM of each component) using NADH and [4R-^2^H]-NADD. KIEs were thus determined as the ratio of the *k*_obs_ values using NADH and NADD at a given temperature. Activation parameters (frequency factor, A, and the activation energy, E_a_) were determined using the Arrhenius equation as described [24].

### 2.5 Proteolysis by trypsin

Stock solutions of trypsin from bovine pancreas (T1426, from Sigma-Aldrich) were prepared at 80–100 μM in 50 mM HEPES-KOH, pH 7.4 and stored at −80 °C in small aliquots upon freezing in liquid nitrogen. Trypsin concentration was determined using *ε*_280_ = 35100 M^−1^·cm^−1^ (according to the manufacturer's instructions). NQO1 samples were diluted to a final concentration of 15 μM in protein subunit in 50 mM HEPES-KOH, pH 7.4. For NQO1_holo_ samples, a final concentration of FAD 100 μM was added, while in NQO1_dic_, final concentrations of FAD and dicoumarol were 100 μM. The reaction mixtures were incubated for 10 min at 25 °C prior to addition of a concentrated trypsin solution (to a final concentration ranging from 5 nM to 2 μM in protease). Aliquots were withdrawn at different time points, mixed with 1/10^th^ volume of 10 mM Phenylmethylsulfonyl fluoride (PMSF, Sigma-Aldrich) in ethanol and then with 1 volume of 4 x Laemmli's buffer and immediately denatured for 5 min at 95 °C. Samples were resolved in 12% acrylamide SDS-PAGE gels, stained with Coomassie® Brilliant Blue R250 (Sigma-Aldrich). Gels were analyzed using ImageJ (https://imagej.nih.gov/ij/). The intensity of the uncleaved (full-length) protein (I) vs. time was used to determine the observed (apparent) rate constant *k*_obs_ from fittings using the following exponential function:

(Eq 3) $$I=I_{0}\cdot exp^{-k_{obs}\cdot t}.$$

From the linear dependence of *k*_obs_ on trypsin concentration, the second-order rate constants *k*_prot_ were obtained. Thus, the change in thermodynamic stability of the CTD due to a mutation or ligand binding (state i), ΔΔG_prot_, can be calculated using a given reference state (for instance, WT NQO1_apo_) using equation 4:

(Eq 4) $$\Delta \Delta G_{prot}=-R\cdot T\cdot ln\left(\frac{k_{prot\left(i\right)}}{k_{prot\left(ref\right)}}\right).$$

Therefore, a negative/positive value of ΔΔG_prot_ reflects that the i state has lower/higher CTD thermodynamic stability than the reference state.

### 2.6 Thermodynamics of dic binding by isothermal titration calorimetry (ITC)

ITC experiments were carried out using an ITC_200_ microcalorimeter (Malvern). NQO1 samples were loaded in the cell at a concentration of 15–17 μM (in monomer) as holo-proteins (+100 μM of FAD). NQO1_holo_ proteins were titrated using Dic (150–180 μM, plus 100 μM FAD) by performing an initial injection (0.5–1 μL) followed by 18–23 injections of 1.5–2 μL each, spaced by 150–210 s. Experiments were carried at 25 °C (at least three independent titrations), 20 °C and 15 °C (a single titration for these temperatures). Data analyses were performed as previously described [13,21]. Briefly, binding isotherms were analyzed considering a single type of independent binding sites, and dilution heats were considered as a fitting parameter. This yielded all apparent binding thermodynamic parameters (K_a_, ΔG, ΔH, ΔS) whereas the apparent change in heat capacity (ΔC_p_) was determined from the linear dependence of the apparent ΔH on temperature.

Apparent ΔH and ΔC_p_ were used to estimate the magnitude of the conformational change triggered by Dic binding as recently described [13]. Briefly, it was considered that these two variables contain a contribution from intrinsic binding (i.e. lock-and-key mechanism) ΔH_int_ and ΔC_p,int_, plus a contribution arising from the confomational change, ΔH_conf_ and ΔC_p,conf_ [35,36]. Intrinsic binding parameters were determined from changes in apolar and polar surface upon Dic binding (−969 Å^2^ and -621 Å^2^, respectively) estimated from high-resolution X-ray structural models of NQO1 with Dic and using well-known structure-energetics correlations [13,36,37]. These correlations provided values of ΔH_int_ = −5.8 kcal mol^−1^ and ΔC_p,int_ = −0.14 kcal mol^−1^. Thus, the difference between experimental (apparent) ΔH and ΔC_p_ and those from intrinsic binding provided the contribution from the conformational change [13,35,36,38]. Previous studies have supported that for Dic binding to NQO1, ΔH_conf_ and ΔC_p,conf_ mainly arise from the conformational transition (i.e. unfolded/partially folded to folded) induced by Dic binding on the CTD and thus, these can be used using well-known structure-energetics for protein folding thermodynamics [13,21,39] to estimate in two different manners the apparent number of residues folded upon binding (N_conf_) that provides an estimate of the magnitude of the conformational change associated with Dic binding (Equations (Eq 5), (Eq 6))):

(Eq 5) ΔH_conf_ = 0.215 · N_conf_

(Eq 6) ΔC_p,conf_ = 0.0138 · N_conf_

Where ΔH_conf_ and ΔC_p,conf_ are expressed in kcal·mol^−1^ and kcal·mol^−1^·K^−1^, respectively. These values of N_conf_ are expressed per NQO1 monomer.

### 2.7 Expression analysis in eukaryotic cells

The mutations P187S and S82D were introduced by site-directed mutagenesis on the wild-type (WT) NQO1 cDNA cloned into the pCINEO plasmid by GenScript (Leiden, The Netherlands). Mutagenesis was confirmed by sequencing the entire cDNA. The resulting construct produces the full-length WT protein containing three extra amino acids (MLA) prior to Met1 but no additional tags.

HAP1 NQO1 knockout cells (HAP-1 NQO1-KO; Horizon, Waterbeach, UK; ref. K9HZGHC006138C004) were cultured in Iscove's modified Dulbecco's medium (IMDM, Lonza, Barcelona, Spain) supplemented with 10% fetal bovine serum (HyClone, GE Healthcare, Barcelona, Spain), 100 U·mL^−1^ penicillin and 100 μg·mL^−1^ streptomycin (Sigma Aldrich, Madrid, Spain) and cultured at 37 °C in a humidified incubator with 5% CO_2_. Cells were transfected using Lipofectamine LTX with Plus Reagent (Thermo Fisher Scientific, Madrid, Spain) and selected using 1 mg·mL^−1^ of G418 (Sigma Aldrich). Cells were treated with riboflavin (100 μM) or riboflavin + Dic (both at 100 μM and from Sigma Aldrich) for 24 h, using untreated cells under the same conditions as controls. For proteasomal inhibition studies, MG-132 (Calbiochem, Merck, Madrid, Spain) was added to the medium (at 10 μg·mL^-1^) for 4 h at 37 °C. After treatment, cells were scrapped and lysed in RIPA buffer (50 mM Tris-HCl, 150 mM NaCl, 0.1% Triton X-100, 0.1% sodium dodecyl sulphate, 1 mM sodium orthovanadate, 1 mM NaF pH 8) with protease inhibitors (COMPLETE, from Roche, Spain). Soluble extracts were denatured using Laemmli's buffer and resolved by SDS-PAGE and transferred to polyvinylidene difluoride membranes (GE Healthcare). Immunoblotting was carried out using primary monoclonal antibodies anti-NQO1 (sc-393736, Santa Cruz Biotechnology) and anti-β-actin (sc-47778, Santa Cruz Biotechnology) from mouse at 1:200 and 1:5000 dilutions, respectively. As a secondary antibody, we used chicken anti-mouse IgG-HRP (sc-516102, Santa Cruz Biotechnology) at 1:2000 dilution. Samples were visualized using luminol-based enhanced chemiluminescence (from BioRad Laboratories). All experiments included untreated cells transfected with WT NQO1 as reference.

## 3 Results and discussion

### 3.1 The mutants S82D and P187S increase the population of partially folded conformations in the native state ensemble of NQO1_apo_

**Fig. 2:**
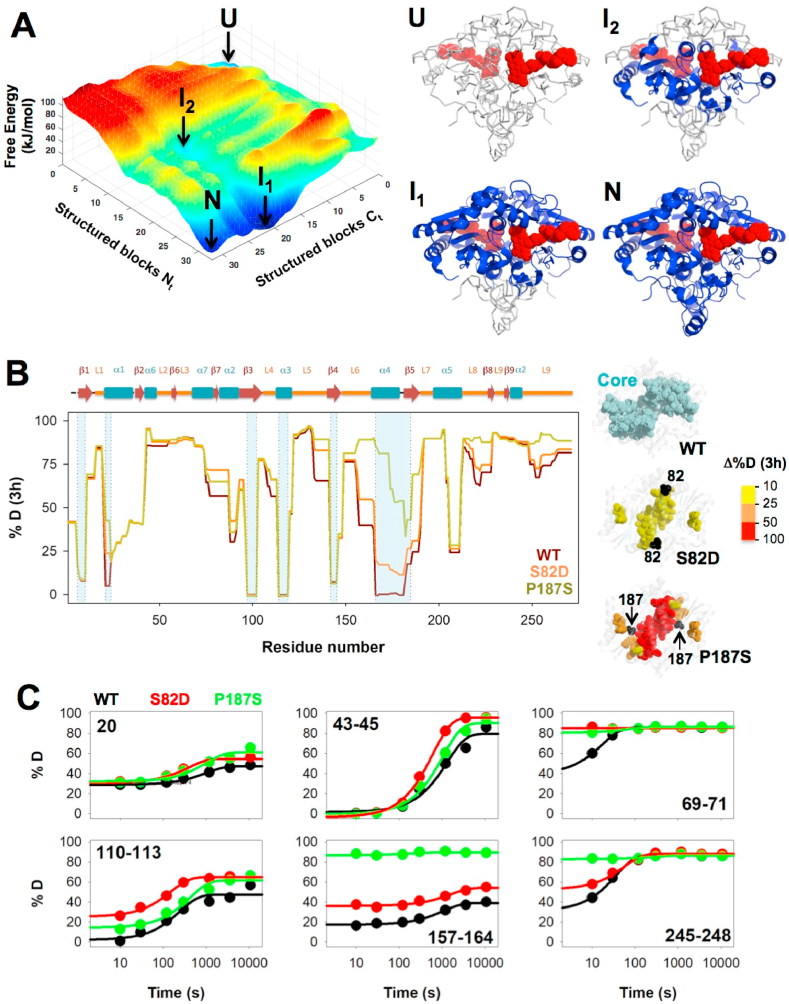
**Effects of S82D and P187S on the conformational ensemble of NQO1**_**apo**_. A) The left panel shows the free energy landscape of WT NQO1_apo_ generated as a function of the number of structured blocks in the NTD and CTD; In the right panel, the structured regions for four relevant conformational substates (N, I_1_, I_2_ and U) are mapped onto the crystal structure of NQO1 as blue cartoon (PDB code 2F1O [25]). For reference, the bound FAD is shown as dot representation in red. B) HDX (%D) after 3 h at 25 °C for WT_apo_, S82D_apo_ and P187S_apo_. Secondary structure elements are shown according to Ref. [19]. Shadowed regions indicate those belonging to the stable core (<20% D after 3 h) [23]. Colour codes in structural representations indicate values of Δ%D after 3 h according to the scale using WT_apo_ as reference. Structural representations (right panels) show the location of the stable core in WT_apo_ as well as the destabilization by S82D and P187S (as a Δ%D after 3 h); C) HDX analysis for WT, S82D and P187S in the NQO1_apo_ showing kinetic heterogeneity along 3 h exchange time and the effect of these two amino acid substitutions. HDX data for WT are from Ref. [23]. (For interpretation of the references to colour in this figure legend, the reader is referred to the Web version of this article.)

Unlike NQO1_holo_ and NQO1_dic_, no high resolution structural information is available for NQO1_apo_ (likely due to its high intrinsic flexibility) [12]. Importantly, local stability analyses by hydrogen/deuterium exchange (HDX) kinetics of NQO1_apo_ showed remarkable heterogeneity across the structure. Different regions of the WT protein showed widely different extent and kinetics of deuterium incorporation (%D) in a 3 h experiment [23]. This behavior suggests that NQO1_apo_ populates different conformational substates under native conditions with very different local stabilities. Presumably, the population of these partially folded states is connected with certain functional features such as FAD binding, proteasomal degradation of the protein, or biomacromolecular interactions [7,8,12]. To further characterize this conformational heterogeneity, we carried out a statistical mechanical analysis with the Ising-like and native-centric WSME (Wako-Saitô-Muñoz-Eaton) model, in which the conformational ensemble of NQO1_apo_ WT is generated from ~1.4 million microstates. The resulting conformational landscape consistently reproduces the heterogeneity of states expected from HDX experiments (Fig. 2A and S7) [23]. In addition to the Native (N) and Unfolded (U) ensembles, at least two partially folded ensembles (I_1_ and I_2_) are significantly populated (Fig. 2A). The I_1_ intermediate contains residues 1–220 folded, whereas the I_2_ intermediate has residues 90–220 folded.

What are the structural-functional features of these intermediates? The intermediate I_1_ is predicted to exhibit a conformation in which the stable core of NQO1_apo_ is folded including those residues critical for FAD binding (i.e. it would have *high affinity* for FAD), whereas the intermediate I_2_ contains 74% of this core in a folded conformation but only a few of the residues key for FAD binding (Fig. 2A–B). The population of conformational states similar to the I_1_ intermediate in NQO1_apo_ is further supported by previous mutagenesis studies in which the CTD (residues 225–274) was excised (i.e. Δ50-NQO1). Δ50-NQO1 stably forms dimers and retains significant binding affinity for FAD (with 8-fold lower than the full-length apo-protein) but reduces NQO1 activity by two-orders of magnitude [21,22,40]. Additionally, the population of these intermediates can be critical in determining efficient ubiquitination and degradation of NQO1 inside cells, which is mainly dictated by the presence of an unfolded CTD [7,12,22,41].

The NQO1_apo_ state shows a marginal stability and the dimeric state is held by a stable core that undergoes minimal HDX after 3 h (Fig. 2B and [23]). Importantly for our study, the mutations S82D and P187S affected this core, and their effects were quantitatively different (Fig. 2B). S82D mildly destabilized segments 21–23 (helix α1) and 166–181 (helix α4) whereas P187S largely destabilized segments 166–178 (helix α4) and moderately destabilized segments 21–23 (helix α1) and 179–185 (sheet β5). Hence, the mutation P187S causes a stronger structural destabilization that also propagates more extensively through the stable core of NQO1_apo_ (Fig. 2B). In addition, HDX kinetics and statistical mechanical analyses support that both P187S and S82D may increase the population of the I_1_ intermediate, and to a lesser extent of I_2_ intermediate, and these population-shift should be more pronounced in the case of the P187S mutant (Fig. 2 and S7-8).

### 3.2 Structural perturbations in the NQO1_holo_ and NQO1_apo_ states largely differ between the S82D and P187S mutants and explain their reduced affinity for FAD

FAD binding to NQO1 WT causes remarkable changes in protein structural stability to form a pre-catalytic and intracellularly stable NQO1_holo_ state [7,12,23,[42], [43], [44]]. Perturbations in the structural stability of the NQO1_holo_ and NQO1_apo_ states by P187S and S82D are likely associated with their altered function and stability [7,10,12,21,22,42,44]. Particularly, alterations of the flavin binding site (FBS) stability are associated with the 10–40 fold lower affinity for FAD observed for the S82D and P187S mutants [[10], [11], [12],^22^]. In addition, perturbations of the structural stability of the CTD by these mutations in the NQO1_holo_ and NQO1_apo_ states could also be associated with increased intracellular degradation and/or catalytic alterations. Thus, we might expect that specific structural perturbations in NQO1_holo_ and NQO1_apo_ due to S82D and P187S would lead to different molecular consequences and to diverse molecular mechanisms associated with their loss-of-function phenotypes. However, to date, no high-resolution experimental study has explained these effects.

**Fig. 3:**
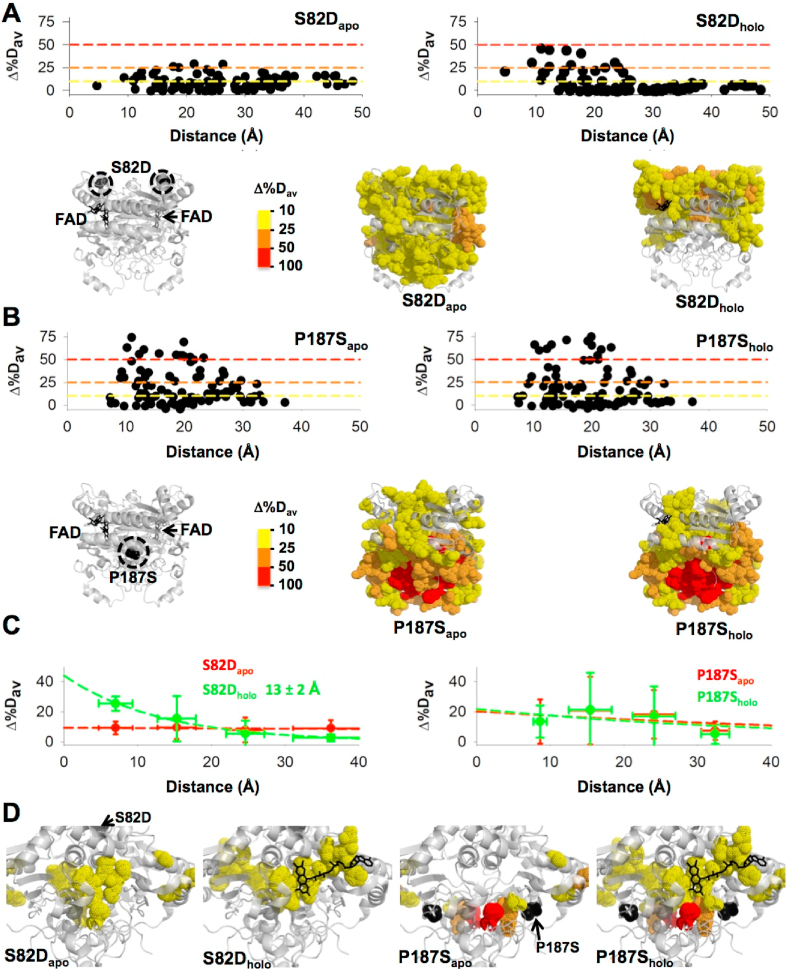
**The effects of S82D and P187S on the structural stability of NQO1**_**holo**_**and NQO1**_**apo**_**using the mutated site as reference (MCI model)**. Effects of S82D (A) and P187S (B) on Δ%D_av_ for NQO1_holo_ and NQO1_apo_. Plots show the values of Δ%D_av_ using the WT protein as a reference in each ligation state as a function of the distance between the mutated site and the different segments evaluated by HDX. Structural representations show the location in the structure of perturbed regions. C) Distance-dependent perturbations shown in panels A and B are grouped according to their distance to the mutated site in four groups (<10 Å, 10–20 Å, 20–30 Å and >30 Å). For segments in each group, distances and Δ%D_av_ values are represented as mean ± s.d. Lines are exponential fits. The value of d_c_ (in Å) is only reported in those cases in which a clear exponential behavior is observed. D) Structural representation of the effects of S82D and P187S on the FBS. Values of Δ%D_av_ larger than 10% are depicted as dot representation. Note that a positive value of Δ%D_av_ indicates destabilization vs. the WT protein. Colour codes in structural representations indicate values of Δ%D_av_ according to the scale shown. In all cases, the structural model PDB code 2F1O [25] was used for display and calculations. (For interpretation of the references to colour in this figure legend, the reader is referred to the Web version of this article.)

To provide quantitative analysis on the perturbations caused by the mutations S82D and P187S on NQO1_holo_ and NQO1_apo_, we evaluated the overall effects of these sequence variations on the structural stability of these two states by comparing their HDX kinetics using the Δ%D_av_ parameter [23](Fig. 3, Figures S8-S9). This parameter reflects changes in amplitudes and/or kinetics of HDX in a single metric, allowing us to compare two given protein species; in this case, we compared a given mutant vs. WT NQO1 in a given ligation state (NQO1_holo_ and NQO1_apo_, respectively) (Fig. 3 and S9-S10).

In NQO1_apo_, both S82D and P187S led to significant local destabilization that propagated to almost the entire structure of the protein, although these effects were much more intense for P187S (Fig. 3A and B). Interestingly, FAD binding led to markedly different effects in these two variants vs. the WT protein. In S82D, structural destabilization in the N-terminal region (residues 1–110) was also observed upon FAD binding, and in the region 57–76 this was even larger than in NQO1_apo_, whereas the mild destabilization found in the C-terminal region of NQO1_apo_ was largely reduced upon FAD binding (Fig. 3A). Thus, perturbations of NQO1_holo_ due to S82D should be considered *more local* to the mutated site. In P187S, part of the destabilizing effects in the N-terminal region (residues 1–110) were reduced upon FAD binding, while the C-terminal region remains largely perturbed even in the NQO1_holo_ state (Fig. 3B). P187S also destabilized to a larger extent the monomer:monomer interface (MMI) than S82D, and this effect was similarly observed in the NQO1_holo_ and NQO1_apo_ states of both mutants (Figure S11). An interesting finding is that both mutations S82D and P187S altered the stability of regions located far from the mutated site in both NQO1_holo_ and NQO1_apo_, although the magnitude of this effect was evidently stronger for P187S and depended on the ligation state (Fig. 3A–B).

To globally evaluate the different impact of S82D and P187S on the structural stability of the NQO1 structure, we first investigated the propagation of stability effects using a model (MCI model) in which the reference system was the mutated site and propagation was considered isotropic (i.e. it just depended on the distance to the mutated site). These analyses further illustrated the different effects of S82D and P187S on protein structural stability (Fig. 3A–C). Destabilization caused by S82D was rather mild in the NQO1_apo_ state, and showed a very weak (and linear) dependence on the distance to the mutated site (i.e. propagated *similarly* to regions close and far from the mutated site). Conversely, in the NQO1_holo_ state, these effects were much stronger and exponentially depended on the distance to the mutated site, with a characteristic distance of ~13 Å (Fig. 3C). In the case of P187S, the perturbations were on average moderate for both NQO1_holo_ and NQO1_apo_ states, and also showed a weak (and linear) distance-dependence from the mutated site affecting regions located in virtually the entire 3D structure (Fig. 3C).

To understand the large decrease in FAD binding affinity caused by S82D and P187S, we analyzed their effects in the structural stability of FBS in NQO1_apo_ and NQO1_holo_ (Fig. 3D). Alterations in the stability of the FBS on either of these states may contribute to reduce FAD binding affinity. Decreased stability of the FBS in NQO1_apo_ would reflect a reduced population of FAD binding competent states and thus, an increased penalization to binding due to the conformational transition (i.e. from binding non-competent to competent states). Reduced stability of the FBS in the bound state would facilitate FAD release (i.e. increase the dissociation rate constant) reflecting destabilization of the complex. The S82D mutation affected mildly the stability of the set of residues in the FBS, both in the NQO1_apo_ and NQO1_holo_ states (Fig. 3D). The effects due to P187S were somewhat different. Although P187S also affected a set of residues in the FBS in both NQO1_apo_ and NQO1_holo_, in some residues these effects were very large (particularly relevant for Tyr156 and His162), and the set of residues affected in this variant as NQO1_holo_ was more extensive (Fig. 3D). Thus, even though both S82D and P187S affect the FAD binding affinity to a similar extent [10,11,22], the molecular details of the alterations in the structural stability of the FBS in NQO1_holo_ and NQO1_apo_ caused by these mutations are clearly different.

### 3.3 Structural perturbations caused by S82D and P187S are differently modulated by dicoumarol binding

Binding of dicoumarol (Dic) to P187S is 6 to 10-fold weaker than to the WT protein [13,21]. The origin of this lower affinity seems to be the thermodynamic destabilization of the CTD of P187S_holo_, that thus required a *folding-coupled-to-binding* process of the CTD to reach binding competent states for Dic binding [12,13,21]. This alteration in the structural stability of the CTD has additional phenotypic consequences: P187S_holo_ is efficiently degraded inside cells through recognition of the unstable CTD by the proteasomal degradation pathways [12,21]. Consequently, the abundance of this variant (in contrast to the WT protein) strongly responds to supplementation with Dic that may reduce ubiquitination of the CTD and consequently its degradation [12,21]. The effect of Dic supplementation on the intracellular stability of the S82D variant has not been investigated so far.

**Fig. 4:**
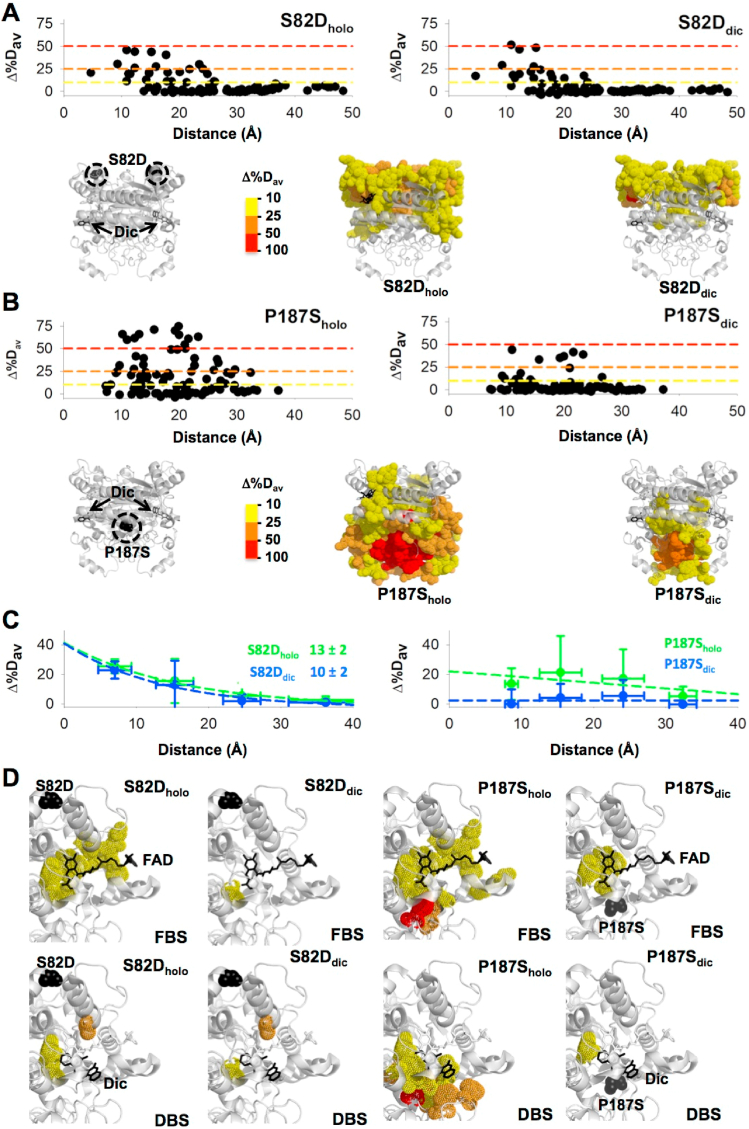
**The effects of S82D and P187S on the structural stability of NQO1**_**holo**_**and NQO1**_**dic**_**(MCI model)**. Effects of S82D (A) and P187S (B) on Δ%D_av_ for NQO1_holo_ and NQO1_dic_. Plots show the values of Δ%D_av_ using the WT protein as a reference in each ligation state as a function of the distance between the mutated site and the different segments evaluated by HDX. Structural representations show the location in the structure of perturbed regions. C) Distance-dependent perturbations shown in panels A and B are grouped according to their distance to the mutated site in four groups (<10 Å, 10–20 Å, 20–30 Å and >30 Å). For segments in each group, distances and Δ%D_av_ values are represented as mean ± s.d. Lines are exponential fits. The value of d_c_ (in Å) is only reported in those cases in which a clear exponential behavior is observed. D) Structural representation of the effects of S82D and P187S on the FBS and DBS. Values of Δ%D_av_ larger than 10% are depicted in dot representation. Colour codes in structural representations indicate values of Δ%D_av_ according to the scale shown. In all cases, the structural model PDB code 2F1O [25] was used for display and calculations. (For interpretation of the references to colour in this figure legend, the reader is referred to the Web version of this article.)

To investigate the effects of Dic binding on the structural stability of S82D and P187S, we carried out HDX kinetic experiments in the presence of Dic (the NQO1_dic_ state) (Figure S12). Binding of Dic to S82D leads to a quite similar structural stabilization to that found for the WT protein (Fig. 4A and Figure S12). Most of the regions that were destabilized in the N-terminal part of the protein (residues 1–110) in S82D_holo_ remained destabilized upon Dic binding, with the exception of two regions that showed enhanced stabilization upon inhibitor binding to S82D vs. WT (regions 11–20 and 107–113). The effects of Dic binding to P187S_holo_ were much more dramatic (Fig. 4B and S12). Dic binding overcome most of the destabilizing effects observed in P187S_holo_ across the entire protein, with the main exception being the CTD in which these effects were reduced but still noticeable (Fig. 4B). Similar analyses focused on these effects on the MMI (Figure S13) yielded essentially the same results (note the remarkable reduction in the MMI of P187S_holo_ upon binding the inhibitor). Application of the MCI model additionally supported the different global response of S82D and P187S upon Dic binding in terms of structural stability (Fig. 4A–C). The exponential dependence of the S82D was similar in the NQO1_holo_ and NQO1_dic_, whereas Dic binding to P187S in the NQO1_holo_ state strongly diminished the destabilizing effect vs. the WT protein (Fig. 4C).

NQO1_holo_ and NQO1_dic_ resemble two functionally relevant states of the enzyme during its catalytic cycle. The former is a pre-catalytic state that is ready for NAD(P)H binding and fast FAD reduction, whereas the latter may represent either the ternary complex with the NAD(P)H or the transition state for the NAD(P)H bound towards the reduced flavin [23]. According to this view, alterations in the structural stability of the FBS and DBS due to S82D and P187S may underlie alterations in the catalytic cycle, in both the reductive and oxidative half-reactions [24]. In WT_holo_, the FBS is particularly stabilized upon FAD binding and also some stabilization is found for the DBS, while binding of Dic leads to strong stabilization of both sites [23]. We have previously interpreted these effects as the formation of a highly *rigid* ternary complex that may allow proper orientation between NAD(P)H and FAD and highly efficient hydride transfer, thus explaining the high rate constant of this process in the WT enzyme [24]. It must be noted that the reductive half-reaction is the rate-limiting step of this catalytic cycle and that communication between the two active sites seems to exist (consistent with negative cooperativity towards NAD(P)H in both steady- and pre-steady state kinetics [24,45]). An important role of the MMI in this allosteric communication has been proposed due to the strong effects of FAD and Dic binding on the stability of this region [23].

We then focused our analyses on the effects of S82D and P187S on the structural stability of the FBS and DBS in NQO1_holo_ and NQO1_dic_ (Fig. 4D). The mutant S82D only showed significant structural destabilization of the FBS as NQO1_holo_, thus suggesting that this mutant might only moderately affect the rate of the reductive half-reaction. The effects of P187S were again much stronger. First, in NQO1_holo_, the structural stability of both the FBS and DBS were largely decreased in this variant, supporting that to form the ternary complex with NAD(P)H competent for flavin reduction, we might expect a larger structural reorganization (and possibly a large entropic penalization; actually this penalization has been observed by titration calorimetry [13,21]). In NQO1_dic_, the FBS of P187S still remains destabilized to some extent which might imply an increased flexilibity in the ternary complex (or the transition state for hydride-transfer reaction) and thus a lower efficiency in the flavin reduction step. All this might contribute to increase the kinetic barrier for the rate-limiting step of the reductive half-reaction due to P187S.

### 3.4 Anisotropic propagation of mutational and ligand binding effects

**Fig. 5:**
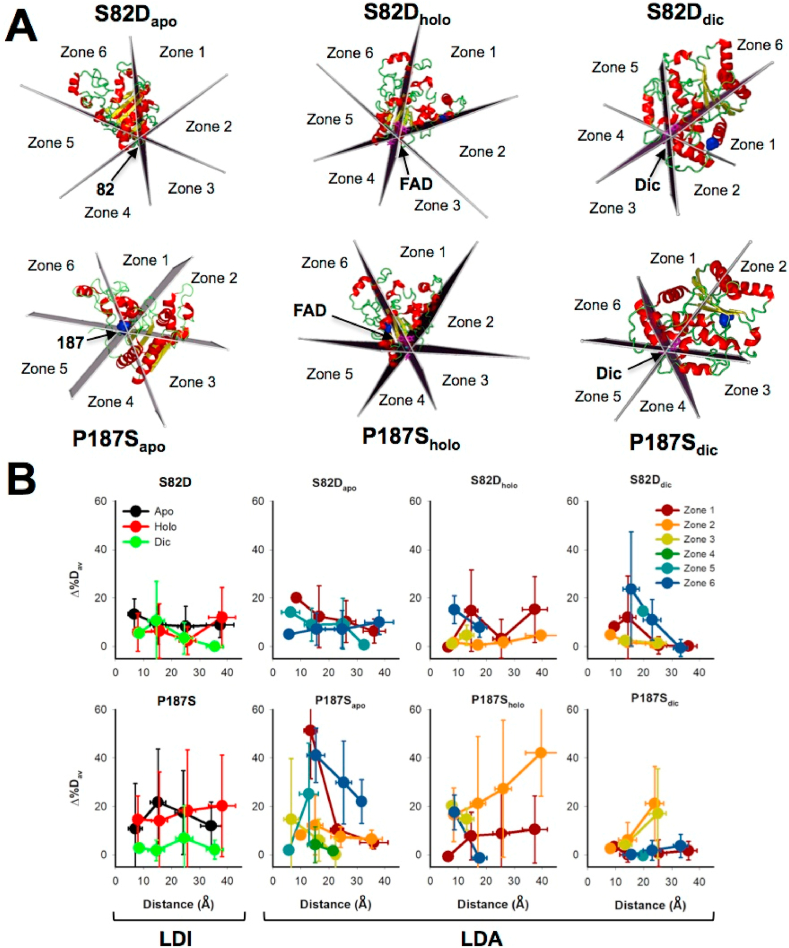
**Anisotropic propagation of mutational effects in different ligation states**. A) Representation of the procedure used to generate different zones for the anisotropic analysis (LDA model). The mutated residue is shown as a blue sphere. B) Isotropic (LDI model) vs. anisotropic (LDA model) analysis of the distance-dependent propagation of mutation- and ligand-binding effects. (For interpretation of the references to colour in this figure legend, the reader is referred to the Web version of this article.)

Visual inspection of the propagation of mutational effects due to S82D and P187S in different ligation states as well as global analysis by the isotropic MCI model (Fig. 3, Fig. 4), strongly supported the existence of anisotropy in the propagation of stability effects which is distinct for different mutations and ligation states. To get a deeper insight into this notion, we have applied two additional models to interpret the results from HDX regarding the propagation of stability effects across the NQO1 structure. In these two models, the reference system depends on the ligation state: for the NQO1_apo_ the reference is the mutated site while in NQO1_holo_ and NQO1_dic_ the reference is the center of mass of the corresponding ligand (for FAD and Dic, respectively). These two models differ regarding the directional propagation of mutational and ligand-binding effects: the LDI model considers isotropic propagation (all directions are considered as equal, and thus, distance is simply related to the perturbed site) whereas the LDA method explicitly considers anisotropic propagation by dividing the structure in six different directions or zones, but maintaining the same concept of distance (Fig. 5A).

The LDI method provides similar results to those of the MCI method (an expected result since both are isotropic approaches) for S82D_apo_ and P187S_apo_ (Fig. 5B). Interestingly, the LDI method shows some differences between S82D and P187S regarding ligand binding (Fig. 5B). The distance dependence of S82D is similar in the NQO1_apo_ and NQO1_holo_ states, whereas upon Dic binding the decay is somewhat more strongly distance-dependent than in the WT protein. P187S showed similar behaviour in the NQO1_holo_ and NQO1_apo_ states, but Dic binding essentially abolished the destabilizing effects of P187S (vs. WT NQO1) (Fig. 5B).

The LDA method provides some interesting insight into anisotropic propagation of stability effects. The entire protein structure is divided into six equal sections called *zones* intersecting at the reference point of each ligation state. The zone 1 contains either the side chain of the mutated site in case of NQO1_apo_ or the center of mass of the ligand interacting residues in case of the bound forms. Subsequent zones were marked in a clockwise manner (Fig. 5A). In simple words, zone 1 along with the adjacent zones 2 and 6 encompass residues in the direction of the perturbation whereas the other zones contain residues opposite to the perturbation direction. Using this method, mutational and ligand-binding effects on stability showed significant directionality (Fig. 5B). Regarding S82D_apo_, zones 1, 5 and 6 show the strongest effects (although milder than those observed for P187S), with zones 1 and 5 showing certain distance-dependence whereas zone 6 does not (Fig. 5B). In S82D_holo_, the strongest effects are observed for zones 1 and 6, although they seem to differ in the distance-dependence propagation (Fig. 5B). In S82D_dic_, the strongest effects are found for zone 6, and to a lesser extent for zone 1, with a noticeable distance-dependence (Fig. 5B). In the case of P187S_apo_, zone 1 and 6 show by far the strongest effects on stability, although their distance-dependences are different (Fig. 5B). The behavior of P187S_holo_ is a little odd, since multiple zones show significant stability perturbations, and for zones 1 and 2, these perturbations are essentially distance-independent (Fig. 5B). The situation for P187S_dic_ is apparently simpler, because destabilizing effects are basically abolished with the exception of some long-range perturbations (about 25 Å) of moderate intensity observed in zones 2 and 3. Overall, these anisotropic perturbation analyses provide at least two interesting conclusions: i) propagation of mutational and ligand-binding effects are quite anisotropic: these occurring in the direction of the perturbation (i.e. via zones 1, 2 and 6) are much more frequent and stronger; ii) the directional preference (in terms of magnitude of the *original* perturbation and the distance-dependence dissipation) for the propagation of mutational and ligand-binding effects strongly depend on both the mutation and the ligand bound.

### 3.5 Effects of S82D and P187S on enzyme catalysis

**Fig. 6:**
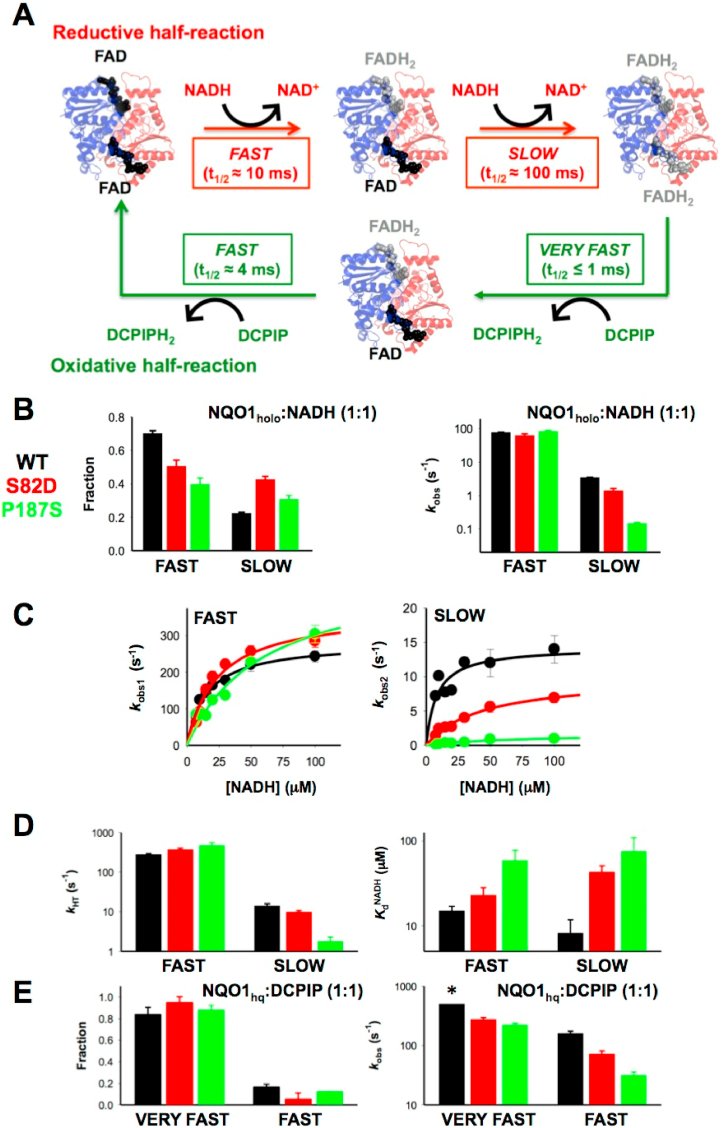
**Effects of S82D and P187S on pre-steady state enzyme kinetics**. A) Schematic representation of the NQO1 WT catalytic cycle (from Ref. [24]). The reductive half-reaction occurs through two main consecutive processes (referred to as *fast* and *slow* steps), likely reflecting the sequential reduction of the two FAD molecules in the NQO1 dimer by NADH with widely different kinetics. Similarly, the oxidative half-reaction occurs through two pathways with very high rate constants, (referred to as *very fast* and *fast* steps) using DCPIP as substrate; B-D) Kinetics for the reductive half-reaction of NQO1 variants by NADH; B) Kinetics using 7.5 μM NQO1:NADH. The left panel shows the fraction corresponding to the fast and slow processes determined as the fractional change in absorbance (average of 445–455 nm) corresponding to the spectral deconvolution (Figure S14). The right panel shows the observed rate constants (*k*_obs_) for each process from kinetic analysis (Figure S14). C) Dependence of the *k*_obs_ values on NADH concentration for the fast (*k*_obs1_) and slow (*k*_obs2_) processes in the reductive half-reaction. Lines are fits to equation (2). D) Limiting values of *k*_obs_ (*k*_HT_) and NADH equilibrium binding constants (*K*_d_^NADH^) for the fast and slow processes in the reductive half-reaction with NADH. E) Kinetics for the oxidative half-reaction of reduced variants (NQO1_hq_) using DCPIP as substrate (7.5 μM NQO1:NADH). The left panel shows the fraction corresponding to the very fast and fast processes determined as the fractional change in absorbance (average of 445–455 nm) corresponding to the spectral deconvolution (see Figure S16 and Table S6). The right panel shows the *k*_obs_ values for the very fast and fast processes. The asterisk indicates that *k*_obs_ > 500 s^−1^. Data for WT NQO1 are reproduced from Ref. [24]. All experiments were carried out at 6 °C. In panels B–E, data correspond to WT (black), S82D (red) and P187S (green). (For interpretation of the references to colour in this figure legend, the reader is referred to the Web version of this article.)

The reductive and oxidative half-reactions of WT NQO1 are not simple processes [24]. The two flavin molecules located in the two active sites of NQO1 WT are reduced with widely different kinetics: one of the FAD molecules is reduced 10-fold faster than the other one in the dimer (*fast* vs. *slow* steps) [24]. In the oxidative half-reaction, one molecule of reduced flavin, FADH_2_, is oxidized extremely fast (*very fast* step) whereas the second FADH_2_ molecule is oxidized more slowly (*fast* step). These features of the catalytic cycle of WT NQO1 are summarized in Fig. 6A. The non-equivalence of the FAD/FADH_2_ molecules bound to the two active sites has been proposed to represent a case of functional negative cooperativity [24].

Our analyses using HDX (NQO1_holo_ vs. NQO1_dic_, regarding the FBS and DBS; Fig. 4D) actually suggested that P187S, and to lesser extent S82D, may affect the catalytic cycle of NQO1. To test this, we first carried out experiments on the reductive half-reaction with the S82D and P187S mutants using equimolar concentrations of NQO1_holo_ and NADH (Figure S14). These experiments revealed some interesting effects (Fig. 6B). These two mutations did not alter the existence of two different pathways in the reduction of the flavin and hardly affected the observed rate constant (*k*_obs_) for the fast step (step A→B, *k*_obs1_) (Fig. 6B). However, at these conditions, the reduction of the second flavin molecule (step B→C, *k*_obs2_) occurred 4.5- and 70-fold more slowly for S82D and P187S, respectively (Fig. 6B). Further experiments to evaluate the NADH concentration dependence of the reductive half-reaction supported mild effects of these mutations on the limiting value of *k*_obs1_ (i.e. *k*_HT1_, where HT refers to hydride-transfer) and revealed a modest 4-fold decrease in *K*_d1_^NADH^ for P187S (Fig. 6C–D and Table S5). Thus, the catalytic efficiency (*k*_HT_/K_d_^NADH^) of S82D in the fast reduction step was similar to that of WT NQO1, while P187S decreased it by 2.4-fold (Table S5). Importantly, the effects of S82D and P187S were much more pronounced for the slow FAD reduction process (Fig. 6C–D and Table S5). The mutation S82D had a marginal effect on *k*_HT2_ but increased by 5-fold the *K*_d2_^NADH^. The effects of P187S were much more pronounced, with a decrease in *k*_HT2_ of 8-fold and an increase of 9-fold in the *K*_d2_^NADH^. Therefore, both mutations significantly affected the slow FAD reduction step, with a decrease in catalytic efficiency of 8-fold and 80-fold for S82D and P187S, respectively (Table S5). These observations were also supported by experiments carried out with NADPH in the reductive half-reaction (Figure S15), or using DCPIP as substrate in the oxidative half-reaction (in this case, NQO1_holo_ had been previously reduced with NADH) (Fig. 6E, Figure S16 and Table S6), although these reactions were too fast to carry out a more detailed characterization [24]. Since the reductive half-reaction is rate-limiting in the catalytic cycle of NQO1 [24], these results suggest that these two mutations (particularly P187S) exacerbate the negative functional cooperativity found in NQO1 WT under pre-steady state conditions. In particular for P187S, one of the active sites became essentially *useless*, thus likely contributing to reduce the specific activity inside the cell and constituting some sort of half-of-sites reactivity. We may speculate that these remarkable effects of P187S, particularly on the slow step, as well as this half-of-sites reactivity, could result from: i) the structural destabilization of NADH binding site (inferred from the effects observed in the FBS and DBS of P187S_holo_, Fig. 4D) that could affect directly catalysis; ii) destabilization of the MMI (Figure S13) that may contribute to alter the communication of stability effects between active sites during the catalytic cycle of NQO1.

**Fig. 7:**
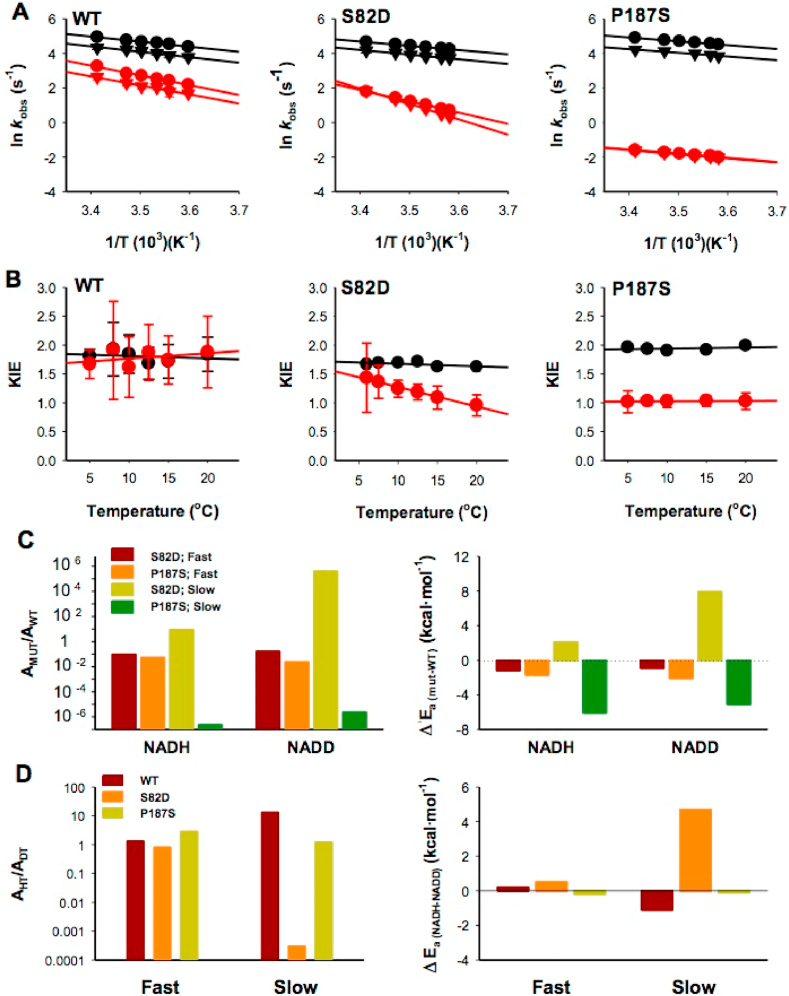
**Temperature-dependent enzyme kinetics and kinetic isotope effects (KIEs)**. A) Arrhenius plots for the reductive half-reaction carried out using NADH (circles) or NADD (triangles). Black and red symbols indicate the kinetics of fast and slow FAD reduction processes, respectively. B) Temperature dependence of KIEs. Experiments were carried out using 7.5 μM NQO1 and NADH/NADD (n ≥ 3; mean ± s.d). C) Effect of the S82D and P187S mutations (vs. WT) on the Arrhenius activation parameters (left panel, frequency factors; right panel, Activation energies). D) Arrhenius activation parameters for each variant using NADH and NADD (left panel, Frequency factors; right panel, Activation energies). (For interpretation of the references to colour in this figure legend, the reader is referred to the Web version of this article.)

To further explore these effects on the catalytic cycle of NQO1, we then carried out experiments on the reductive half-reaction to compare the behaviour towards NADH and NADD. These experiments were carried out using equimolecular concentration of enzyme and coenzyme to overcome technical limitations (particularly for the temperature-dependent studies) [24]. These experiments yielded the kinetic isotope effects (KIE) for both the fast and slow FAD reduction steps (Fig. 7A–B and Table S7). The first point to note is that when using NADD the fast reduction step is slowed down, resulting for all variants in similar KIEs (1.8–2.0). These relative low KIEs have been associated with the transition state being asymmetrical or nonlinear [24,46]. However, some differences were observed again for the slow reduction step. Basically, this slow step shows the same KIE as the fast step for WT NQO1, but its value is lower in the mutants, becoming essentially 1 for P187S (Fig. 7A–B and Table S7). This result reinforces the notion of P187S (and to a lower extent, S82D) affecting much more strongly the kinetics and activation energetics of the slow reduction pathway of FAD. In addition, these KIEs were essentially temperature-independent for both fast and slow FAD reduction steps in all variants (Fig. 7A–B), with the exception of the slow step for S82D. Further analyses of these results in the context of the Arrhenius equation revealed some additional effects of these mutations on the activation parameters of the reaction (Fig. 7C–D and Table S7). Using either NADH or NADD as coenzyme, the *fast* step was marginally affected by S82D and P187S mutations regarding the values of frequency factors (A, less than one order of magnitude) and activation energies (E_a_). However, more interesting differences were found (again) in the *slow* FAD reduction step: the S82D mutation increased the value of the frequency factor and activation energy to some extent, but the effects of P187S were much more dramatic, with a decrease in the preexponential factor of 6–7 orders of magnitude and a remarkable decrease in the activation energy of over 5 kcal mol^−1^ (vs. WT) (Fig. 7C). A simple calculation (based on the transition state theory) yield an increase in the activation free energy for the slow step of 2.0–2.5 kcal mol^−1^ for P187S (using NADH or NADD), thus supporting a noticeable entropic penalization for this step in P187S vs. the WT protein. A potential explanation for this is that P187S requires a larger structural reorganization to bind NADH/NADD to engage in the catalytic process, which actually agrees with our HDX analyses that showed a larger change in structural stability from NQO1_holo_ to NQO1_dic_ in the DBS of P187S (Fig. 4D).

KIEs and activation parameters serve to prove the nature of the chemical step and the effects of mutations on the structural organization and dynamics at the active site during catalysis [47,48]. For the fast FAD reduction step in all three variants, KIEs are temperature independent and above unity. Together with values of A_H_/A_D_ ≠1, these observations indicate that the mutations do not prevent the tunnel contribution to the fast HT process as reported for the WT protein [24]. In addition, the ΔE_aD–H_ values close to zero indicate in general a *tunnelling ready state* (TRS) where the donor-aceptor-distance (DAD) has been reduced during complex pre-organization motions. In this situation, *passive dynamics* (i.e. movements of active site heavy atoms that do not actively contribute to the tunnel probability but increase that to achieve tunnel ready conformations) is sufficient to bring the donor and acceptor to an adequately short distance for efficient tunnelling and HT. This suggests that S82D and P187S hardly impact the architecture and dynamics of the active site for the fast FAD reduction, and thus, the decreased HT efficiency in P187S simply arises from the lower affinity for NADH (Fig. 6C–D and Table S5). However, different contributions from dynamics to the slower FAD reduction process were observed for the S82D and P187S mutants. In WT NQO1, data were consistent with a native-like pre-organization and increased contribution of passive dynamics (ambiental) reorganization (i.e. increased A_H_/A_D_ ratio) to achieve HT relative to the faster process. On the contrary, the temperature-dependent KIE, low A_H_/A_D_ ratio and increased ΔE_aD–H_ for S82D are indicative of only H-tunnelling and of a DAD sampling coordinate (*gating* or active dynamics; i.e. environmental vibrational enhancement that alters the DAD and as consequence energy barrier for tunnel) starting to significantly contribute when packing defects both alter/enlarge the initial DAD and decrease the force constant of the local DAD sampling mode. This is a typical situation when thermal energy is required to maintain short DADs and H-tunnelling. Finally, lack of KIE and close to unity A_H_/A_D_ ratio in P187S suggest that tunnelling does not contribute to the slow HT event in this variant. Such observation agrees with the reduction in k_HT2_ as well as in coenzyme affinity (Fig. 6C–D and Table S5), indicating that the P187S mutation produces a negative impact in the pre-organization motions required to achive a native-like competent conformation for HT at the active site that carries out the slow step. In conclusion, the S82D and P187S mutations hardly affect the organization and dynamics of the active site for the faster HT from NADH/D to the coenzyme, but have an important negative impact in the preorganization motions to achive the reactive active site for the slower HT event. Thus, in the *slow* reduction gating partially overcomes the negative impact on preorganization for S82D, but not for P187S.

### 3.6 The intracellular stability of NQO1 is differentially modulated by ligand binding in S82D and P187S through effects on the CTD stability

The stability of the CTD is key to understand the intracellular degradation of NQO1 through the ubiquitin-dependent proteasomal activity [7,12,26]. In WT NQO1, under standard riboflavin supplementation, most of the protein likely populates the FAD bound state, whereas riboflavin starvation promotes the accumulation of newly synthesized NQO1_apo_ that is efficiently targeted to degradation through its CTD [7]. In P187S, destabilization of the CTD even in the NQO1_holo_ state has been associated with its low intracellular stability due to enhanced ubiquitination of this domain that accelerates degradation [7,21]. Consistently, Dic binding stabilizes the CTD and protects P187S towards ubiquitin-dependent proteasomal degradation in cells [7,21].

**Fig. 8:**
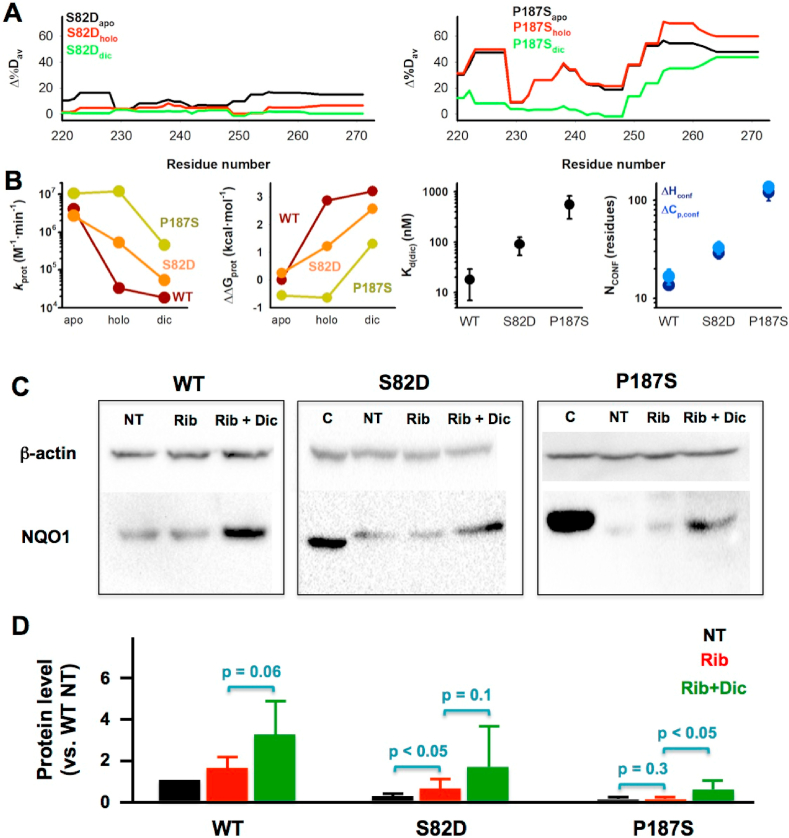
**The stability of the CTD determines the intracellular protein levels of NQO1 variants and their response to Dic supplementation**. A) Changes in local stability of the CTD for S82D and P187S variants in different ligation states (vs. WT); B) Thermodynamic stability of the CTD for S82D and P187S derived from proteolysis kinetics with trypsin (left panels, see Figures S17 and S18) and structure-energetic analysis of Dic binding (right panels, see Figure S19). C) Representative western-blot analysis of HAP-1 NQO1-KO cells transfected with NQO1 variants. NT indicates no treatment, Rib, treatment with riboflavin; Rib + Dic, treatment with riboflavin and Dic. C indicates a control from cells transfected with WT NQO1 without treatment; D) Densitometric analysis of western-blots. Data are from at least 4 technical replicates (using samples from two independent transfections) and are normalized using WT NQO1 without treatment. Statistical analyses were carried out from one-tailed t-tests and significance reported as *p* values.

Our HDX analyses revealed that the CTD of S82D is moderately destabilized in NQO1_apo_, whereas this effect is smaller as NQO1_holo_ or NQO1_dic_ (Fig. 8A). On the contrary, the CTD of P187S is largely destabilized in all ligation states, although some reduction of this destabilizing effect is observed upon Dic binding (Fig. 8A).

To further analyze these effects, we first used proteolysis by trypsin, that provides a partial proteolysis pattern consistent with initial cleavage at the CTD rendering an intermediate containing the N-terminal 240 residues [41](Figure S17). For all three variants in different ligation states, proteolysis kinetics was carried out at conditions (i.e. low protease concentration) in which the proteolysis step was rate-limiting (Figure S18). Thus, comparison of the proteolysis rate constants allows to obtain changes in thermodynamic stability of the CTD upon mutation or ligand binding (Fig. 8B, Figure S18 and Table S8) [12,49]. As NQO1_apo_, the CTD in all variants is very sensitive to degradation by trypsin. FAD binding to WT NQO1 increases the stability of the CTD by ~2.9 kcal·mol^−1^, and this cofactor-induced stabilization is severely reduced for the mutants, with S82D and P187S showing a stabilization of the CTD of ~1.6 and ~0 kcal·mol^−1^ upon FAD binding. Dic binding slightly increased the stability of the CTD in WT NQO1 (by ~ 0.4 kcal·mol^−1^) and this stabilization was larger for the mutants (~1.4 kcal·mol^−1^ and ~1.9 kcal·mol^−1^, for S82D and P187S respectively). Overall, we can conclude from these experiments that the CTD of S82D is moderately destabilized in the NQO1_holo_ state (by ~ 1.6 kcal·mol^−1^ vs. WT NQO1), whereas in P187S, this destabilization is larger as NQO1_holo_ (~3.5 kcal·mol^−1^) than for NQO1_dic_ (~1.9 kcal ·mol^−1^).

As a complementary approach to investigate the folding/stability of the CTD, we carried out calorimetric titrations with Dic using NQO1_holo_ variants (Figure S19 and Table S9). The apparent binding affinity (*K*_d_), as well as the apparent binding enthalpies (ΔH) and heat capacities (ΔC_p_) can be used to estimate the extent of the conformational change associated with Dic binding (Equations (Eq 3), (Eq 4)) [13]. Dic binds to WT NQO1 tightly, and binding is enthalpy-driven and displays a moderate binding heat capacity (Table S9). Using well-known structure-energetic relationships, a value for the parameter N_conf_ (related to the magnitude of the *conformational change* upon binding) of about 10–20 residues can be estimated from these thermodynamic variables in WT NQO1 (Fig. 8B), consistent with the minimal conformational rearrangements observed crystallographically [25]. Similar analyses carried out with the mutants S82D and P187S reveal clearly different behaviours (Fig. 8B and Table S9). S82D shows about 5-fold lower affinity than the WT protein, and binding proceeds with a more unfavourable entropic contribution (~4 kcal·mol^−1^) that is partially compensated by a more favourable enthalpy change. Structure-energetics analyses on this mutant yield values of N_conf_ of ~30–40 residues (Fig. 8B), supporting a mild but noticeable increase in the magnitude of the conformational change associated with Dic binding vs. WT. These results, combined with our HDX analyses, suggest that S82D mildly destabilizes the CTD in the NQO1_holo_ state. The behaviour of P187S regarding Dic binding is vastly different (Fig. 8B and Table S9). Its affinity for Dic is reduced by ~30-fold, and the thermodynamic signature is similar to the folding of a small protein upon Dic binding: a 25 kcal·mol^-1^ entropic penalization that is largely compensated by a favourable enthalpic contribution (Table S9). Structure-energetics calculations on this variant support that about 100 residues must fold to bind Dic, consistent with an extreme destabilization of the CTD in this variant, particularly as NQO1_holo_ (Fig. 8A–B).

We observed, at least qualitatively, good agreement on the effects of S82D and P187S on the stability of the CTD in different ligation states and using different approaches (HDX, proteolysis and ITC). Basically, S82D causes mild to moderate effects on the stability of the CTD, although these can be larger as NQO1_holo_, whereas P187S strongly destabilizes the CTD in all ligation states, although this effect is smaller in the NQO1_dic_ state. Assuming that the thermodynamic stability of the CTD mainly dictates the intracellular stability of NQO1 variants, these analyses allow us to hypothesize that: i) in the absence of Dic supplementation, the intracellular levels of S82D (as a proxy for their proteasomal degradation) should be intermediate between those of WT and P187S; ii) Dic supplementation should boost P187S protein levels (i.e. intracellular stability) to a higher extent than those of the mutant S82D.

To test these hypotheses, we stably transfected HAP-1 cells (that are knocked-out in NQO1) with NQO1 WT and the mutants P187S and S82D, and we grow them in a standard medium (IMDM) with or without supplementation with riboflavin (Rib) or Rib + Dic, and determined their protein levels by western-blot (Fig. 8C–D and Figure S20). The first point to note was that with or without riboflavin supplementation, protein levels of S82D were reduced to 15–20% of those of WT NQO1, whereas the effect of P187S was much larger (≤5% of WT NQO1). Treatment with the proteasomal inhibitor MG-132 led to a 4.5- (for P187S) and 1.8-fold (for S82D) increase in protein levels, whereas this treatment had no effect on WT NQO1 (Figure S20). These results are consistent with enhanced proteasomal degradation of P187S compared to S82D, which is very slow for WT NQO1. Secondly, supplementation with Dic notably increased the protein levels of P187S (about 7-fold vs. Rib treatment), and this effect was weaker for S82D and WT NQO1 (about 3-fold and 1.8-fold vs. Rib treatment, respectively). Therefore, these results supported our hypothesis linking the difference in protein abundance (as a proxy for intracellular degradation) and the response to Dic treatment to the specific effects of these mutations on the stability of the CTD in different ligation states.

## 4 Conclusions

The multiple functional features displayed by human flavoproteins (involved in metabolic reactions, regulatory biomacromolecular interactions, transport to different subcellular locations, intracellular stability) can be modulated by ligand binding, disease-associated mutations and post-translational modifications. However, these complex structure-function relationships are generally not well undestood, in part due to the lack of high-resolution structures in most cases. In this work, we investigated these relationships using the human NQO1 as a model of multifunctional protein [16], and evaluated the effect of two mutations, the cancer-associated polymorphism P187S and the phosphomimetic mutation S82D on several functional traits. Our results revealed that the local destabilization of the protein structure caused by these two mutations can be transmitted to distant functional sites, and thus, affect different functional features to different extents, including enzyme catalysis, functional cooperativity, intracellular abundance (i.e. stability) and pharmacological response to natural ligands. Since most of the human flavoproteins are associated with inherited diseases [2] and are potential targets of multiple post-translational events [10], the approach used in our work can be of general application to unravel these complex structure-function relationships in the human flavoproteome, in particular when high resolution structural information is unavailable or hard-to-get. In addition, the ability of mutations and post-translational modifications to affect multiple functional features in human flavoproteins also stress the importance of understanding the allosteric response to ligand binding, disease-associated and post-translational modifications in order to decipher their roles in physiological and pathological conditions.

## Funding

JLP-G and ALP were supported by the ERDF/Spanish Ministry of Science, Innovation and Universities—State Research Agency (Grant RTI2018-096246-B-I00) and Consejería de Economía, Conocimiento, Empresas y Universidad, Junta de Andalucía (Grants P11-CTS-7187 and P18-RT-2413). NM-T was supported by Aula FUNCANIS-UGR. ES was supported by the ERDF/Spanish Ministry of Science, Innovation and Universities—State Research Agency (Grant SAF2015-69796). Access to an EU_FT–ICR_MS network installation was funded by the EU Horizon 2020 grant 731077. EA-C and MM were supported by the Spanish Ministry of Science and Innovation—State Research Agency (Grant PID2019-103901 GB-I00) and Gobierno de Aragon-FEDER (Grant E35_20R). Support of the BioCeV center (CZ.1.05/1.1.00/02.0109) and the CMS/CIISB facility (MEYS CZ - LM2018127) is also gratefully acknowledged. ANN was supported by grants BT/PR26099/BID/7/811/2017 from Department of Biotechnology (DBT, India) and MTR/2019/000392 from Science, Engineering and Research Board (SERB, India).

## Declaration of competing interest

None.
