# Supplementary material for: Structural basis of the pleiotropic and specific phenotypic consequences of missense mutations in the multifunctional NAD(P)H:quinone oxidoreductase 1 and their pharmacological rescue
Source: Redox Biol. 2021 Aug 18;46:102112. doi: 10.1016/j.redox.2021.102112 (PMC8455868; doi:10.1016/j.redox.2021.102112)
Supplement: Multimedia component 1 [file mmc1.docx]

**Structural basis of the pleiotropic and specific phenotypic consequences of missense mutations in the multifunctional NAD(P)H:quinone oxidoreductase 1 and their pharmacological rescue.**

Juan Luis Pacheco-Garcia, Ernesto Anoz-Carbonell, Pavla Vankova, Adithi Kannan, Rogelio Palomino-Morales, Noel Mesa-Torres, Eduardo Salido, Petr Man, Milagros Medina, Athi N. Naganathan and Angel L. Pey.

**Structure-based protein stability analyses**

Structure-based protein stability analyses were performed using the FoldX 4 force field [[1](#_ENREF_1)]. Protein structures were first relaxed to reduce the global energy by using the *RepairPDB* command. Mutations or phosphorylated residues were introduced in the structures (see Table S1 for PDB codes and list of mutated sites) by using the *BuildModel* command with parameters set as 298 K, pH 7, ionic strength 50 mM and VdW design 2.

The free energy changes (∆∆G) obtained represent the difference in folding free energy between the mutant and the reference state (i.e. the WT protein) and were normalized by protein monomer. The accessible surface area (ASA) was evaluated at the residue level using these crystal structures and the software GetArea [[2](#_ENREF_2)] (http://curie.utmb.edu/getarea.html). Statistical analyses of stability changes between different groups of variants were performed using the language and environment for statistical computing R (V3.3.2) and graphs were generated using the GGPLOT2 package [[3](#_ENREF_3)].

A recent report has evaluated the effect of flavin starvation in the proteasomal degradation on 66 flavoproteins in cultured cells, thus accounting the behavior of 2/3 of the entire flavoproteome [[4](#_ENREF_4)]. From this set, we selected 17 proteins to carry out structure-based stability calculations due to disease-associated mutations and phosphorylation events since they all fulfilled all the following criteria: i) at least one suitable crystal structure was available (preferably, and in most of the cases, of the full-length protein; these were retrieved from the Protein Data Bank; https://www.rcsb.org/); ii) phosphorylation sites (at S, T and/or Y) were already reported by large-scale proteomic studies (these were mined from PhosphoSitePlus®; https://www.phosphosite.org); iii) disease-associated mutations were reported (either in OMIM, https://www.omim.org/, or ClinVar, https://www.ncbi.nlm.nih.gov/clinvar/databases) and we selected them since they cover a wide range of mutations (particularly at the level of the physicochemical changes caused in the side-chain upon mutation and solvent exposure in the structure)(Table S2). Using this set, we analyzed the effects of 75 disease-associated mutations and phosphorylation at 152 sites (including phosphorylation of S, T or Y) on the conformational stability of seventeen human flavoproteins (Table S2). Disease-associated mutations destabilized the protein (in terms of folding free energy) on average by 4.0 kcal·mol^-1^ and phosphorylation events by 3.0 kcal·mol^-1^ (with median values of 2.5 and 1.6 kcal·mol^-1^, respectively) (Figure S1A). The apparently lower destabilizing effect of phosphorylation *vs.* disease-associated mutations seemed to particularly arise from the lower penalty associated with phospho-serine sites. Among the different sets of phosphorylation sites, phospho-S showed an average penalty of 1.8 kcal·mol^-1^ (median of 0.7), whereas phospho-T and phospho-Y had averages of 4.1 and 3.7 kcal·mol^-1^, respectively (medians of 3.1 and 2.1 kcal·mol^-1^). These sets of sites displayed a multi-modal profile consistent with the global mutational-effects on protein thermodynamic stability [[5](#_ENREF_5)], and thus likely arose from the different solvent accessibility of the mutated sites (Figure S1B). Accordingly, when the stability effects due to phosphorylation and disease-associated mutations were clustered in two groups depending on site solvent accessibility (buried *vs.* solvent-exposed), it was evident that modification of buried sites was generally associated with a much larger destabilizing effect (Figure S1C). Overall, these analyses supported that destabilization due to disease-associated mutations and phosphorylation followed similar patterns and might be of similar magnitude at the flavoproteome scale.

**Figure S1. Structure-based stability analyses on the effects of disease-causing mutations and phosphorylation events in human flavoproteins.** A) Box plots for the stability effects of 75 disease-causing point mutations and 152 phosphorylation events in seventeen human flavoproteins (see Table S2 for a detailed list). The plot also showed the effect of phosphorylation events distinguishing between serine, threonine and tyrosine sites (phospho-S, phospho-T and phospho-Y). Boxes show inter-quartile ranges and thick horizontal lines show median values. B) Distribution of stability effects displayed with violin plots. Data shown are those in panel A without outliers. C) Box plots for the stability effects of data shown in panel B and grouped according to the solvent accessibility of the sites (buried, ≤10% of ASA, white; solvent-exposed, >10% of ASA, grey). Closed circles in panels A and C show outlier values beyond 1.5 times the inter-quartile ranges.


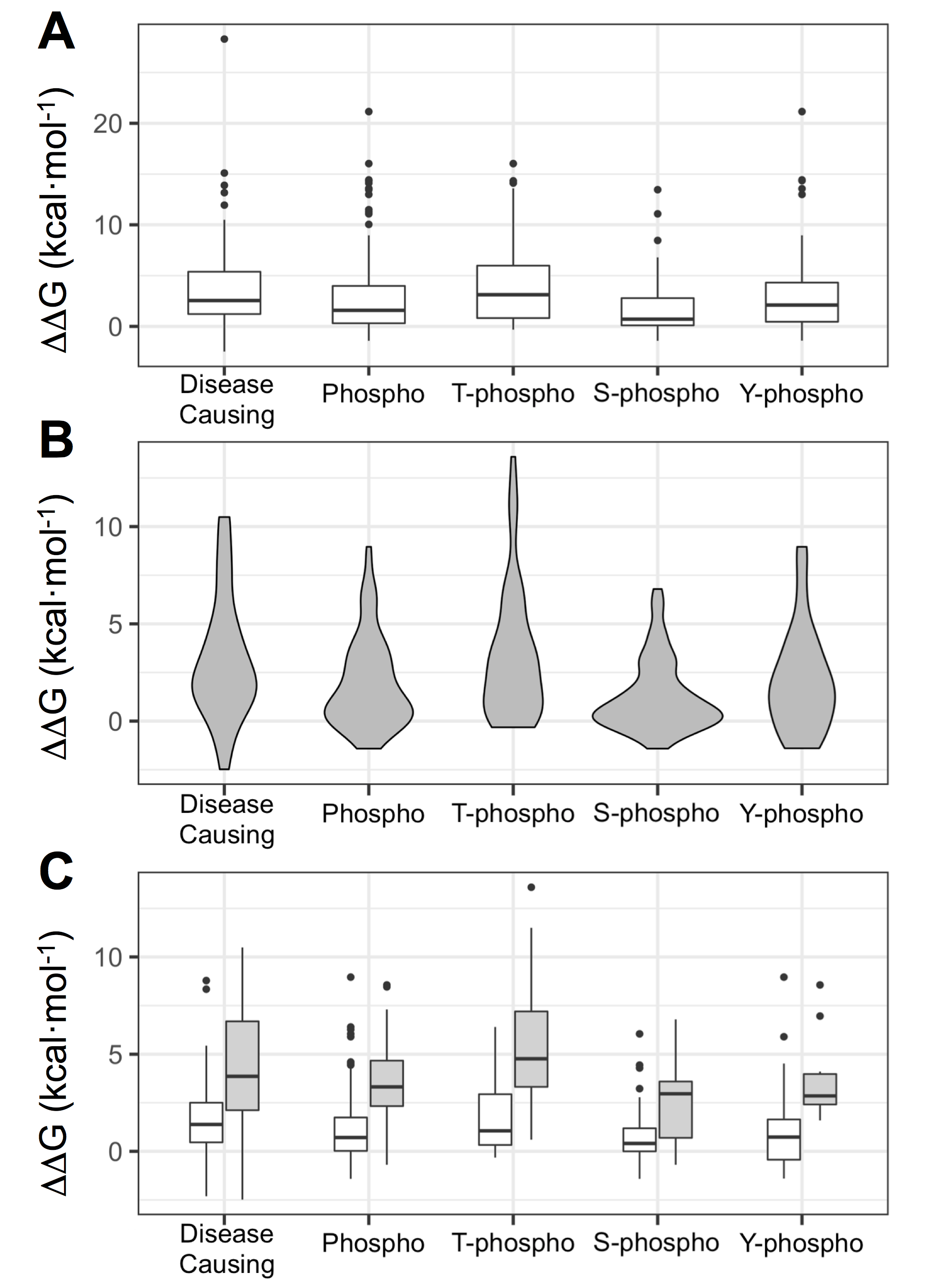


**
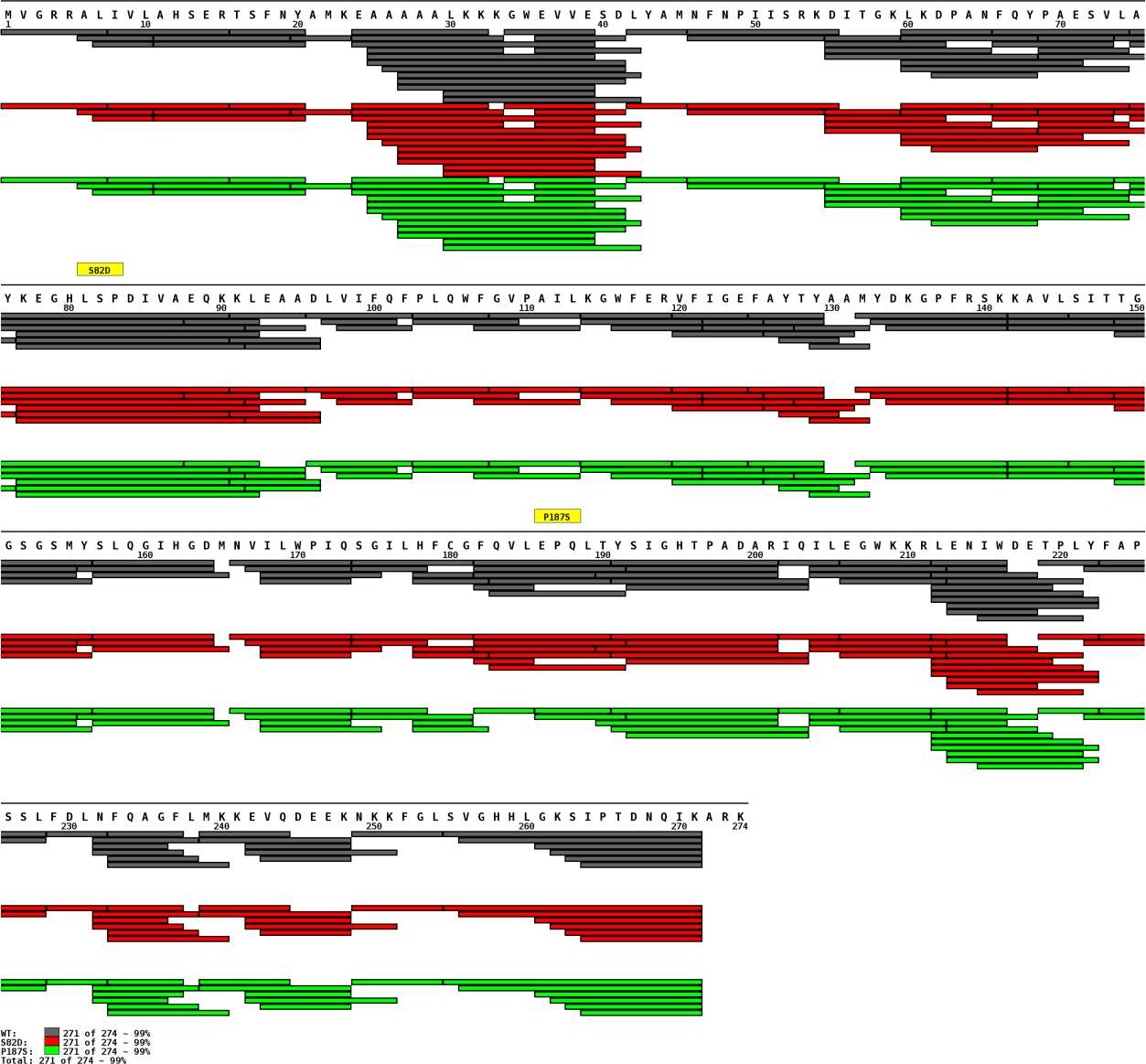
Figure S2. Coverage map for all three studied NQO1 forms** - **wild-type (grey)**, **S82D (red)** and **P187S (green)** variants. Sites of mutations are highlighted above the sequence. Only peptides providing HDX data are shown. Peptide sets are virtually identical, small differences are just between WT/S82D and P187S in the region covering residue 187.

**Figure S3. Deuterium uptake plots for all detected peptides of NQO1_apo_ state. Wild-type (black), S82D (red) and P187S (green).**

*
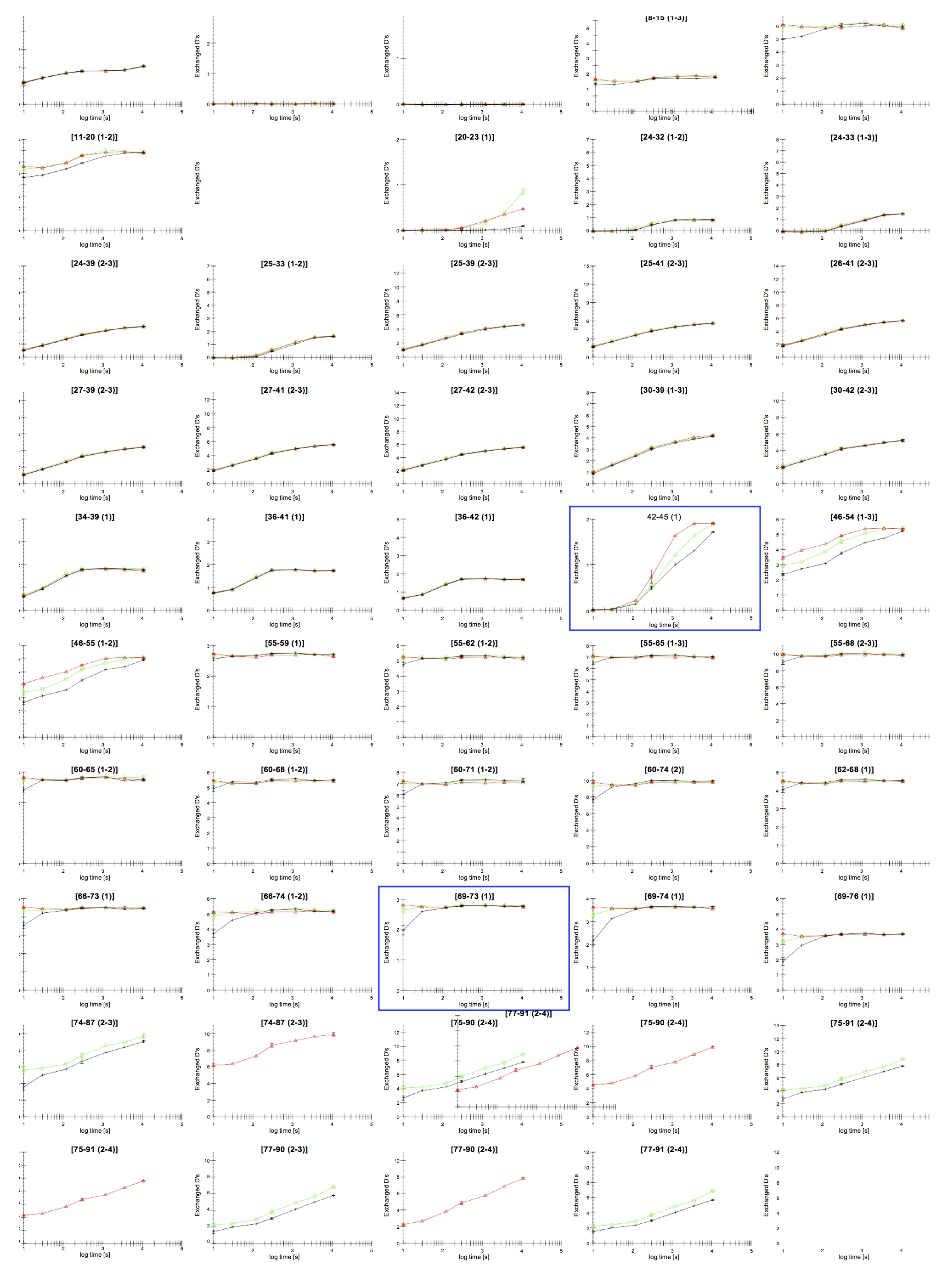
*


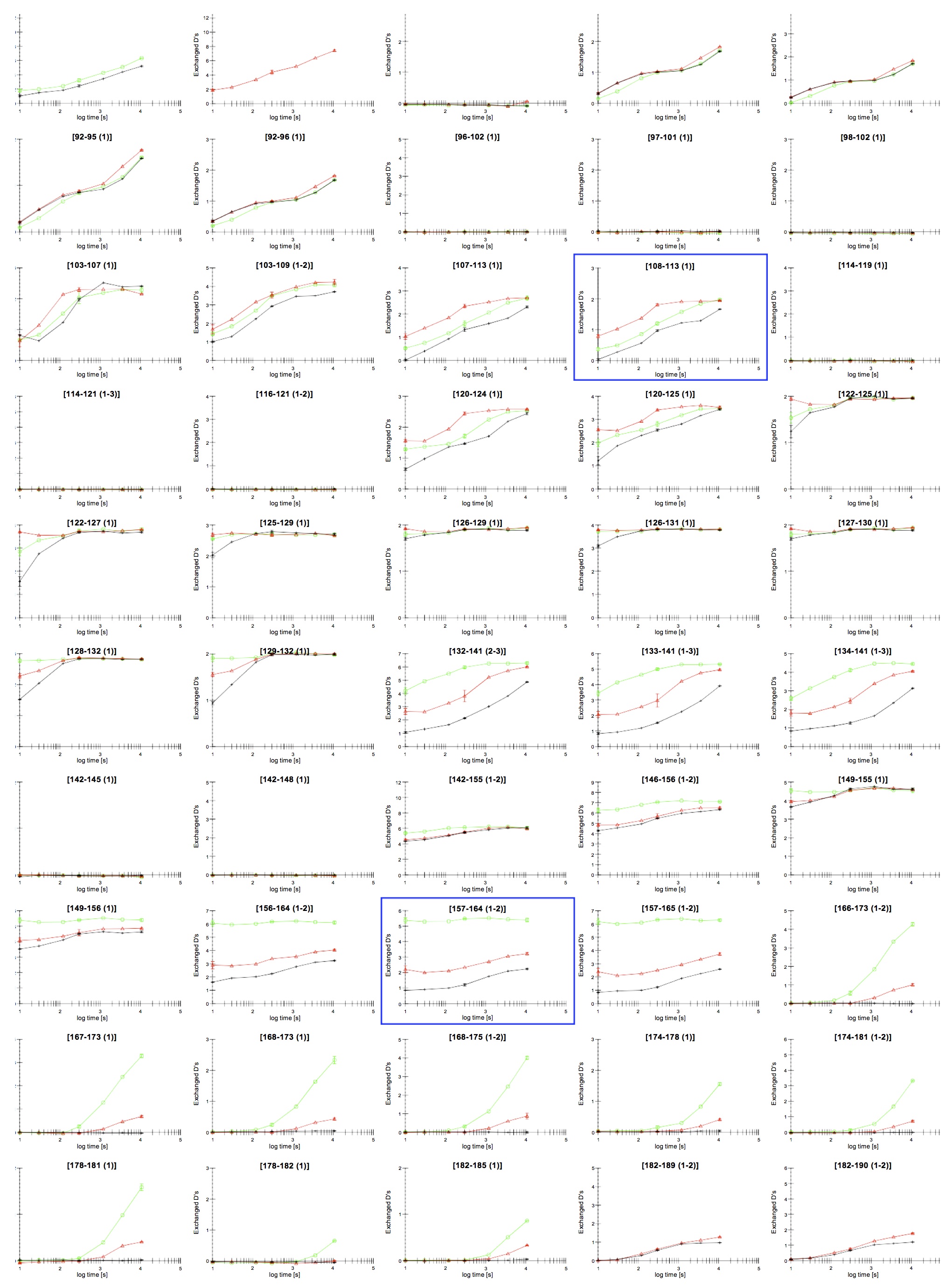


**
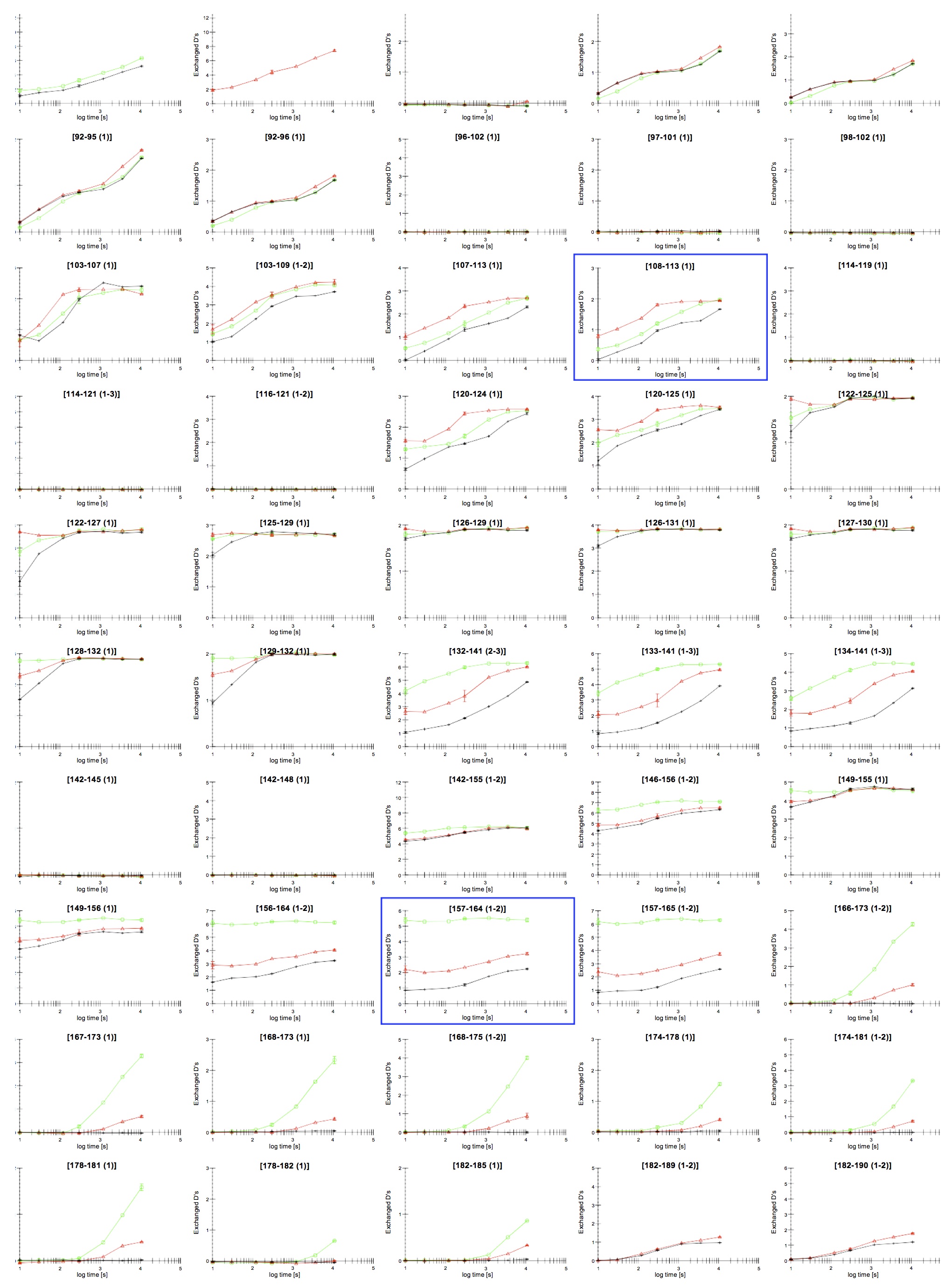
**

**Figure S4. Isotopic profiles for selected peptides derived from NQO1_apo_** (16-20, 42-45, 69- 73, 108-113, 157-164, 242-248 – deuterium uptake curves highlighted by a blue box in the set of plots above) corresponding to regions shown in the Figure 2C of the main text. Isotopic profiles for the individual time points of HDX kinetics for peptides: **(A)** 16-20 (TSFNY, theoretical *m/z* 631.2722 – non-deuterated, 1+) derived from the NQO1_apo_ state; **(B)** 42-45 (LYAM, theoretical *m/z* 497.2428 – non-deuterated, 1+) derived from the NQO1_apo_ state; **(C)** 69-73 (PAESV, theoretical *m/z* 502.2508 –
non-deuterated, 1+) derived from the NQO1_apo_ state; **(D)** 108-113 (GVPAIL, theoretical *m/z* 569.3657 – non-deuterated, 1+) derived from the NQO1_apo_ state; **(E)** 157-164 (SLQGIHGD, theoretical *m/z* 826.4054 – non-deuterated, 1+) derived from the NQO1_apo_ state; **(F)** 242-248 (EVQDEEK, theoretical *m/z* 876.3945 – non-deuterated, 1+) derived from the NQO1_apo_ state.

In all cases the graphs are ordered from top to bottom – ND (non-deuterated), 10s, 30s, 2min, 5min, 20min, 1h, 3h and FD (fully-deuterated). Left column – wild-type (black), middle column – S82D (red), right column - P187S (green).

**S4A**


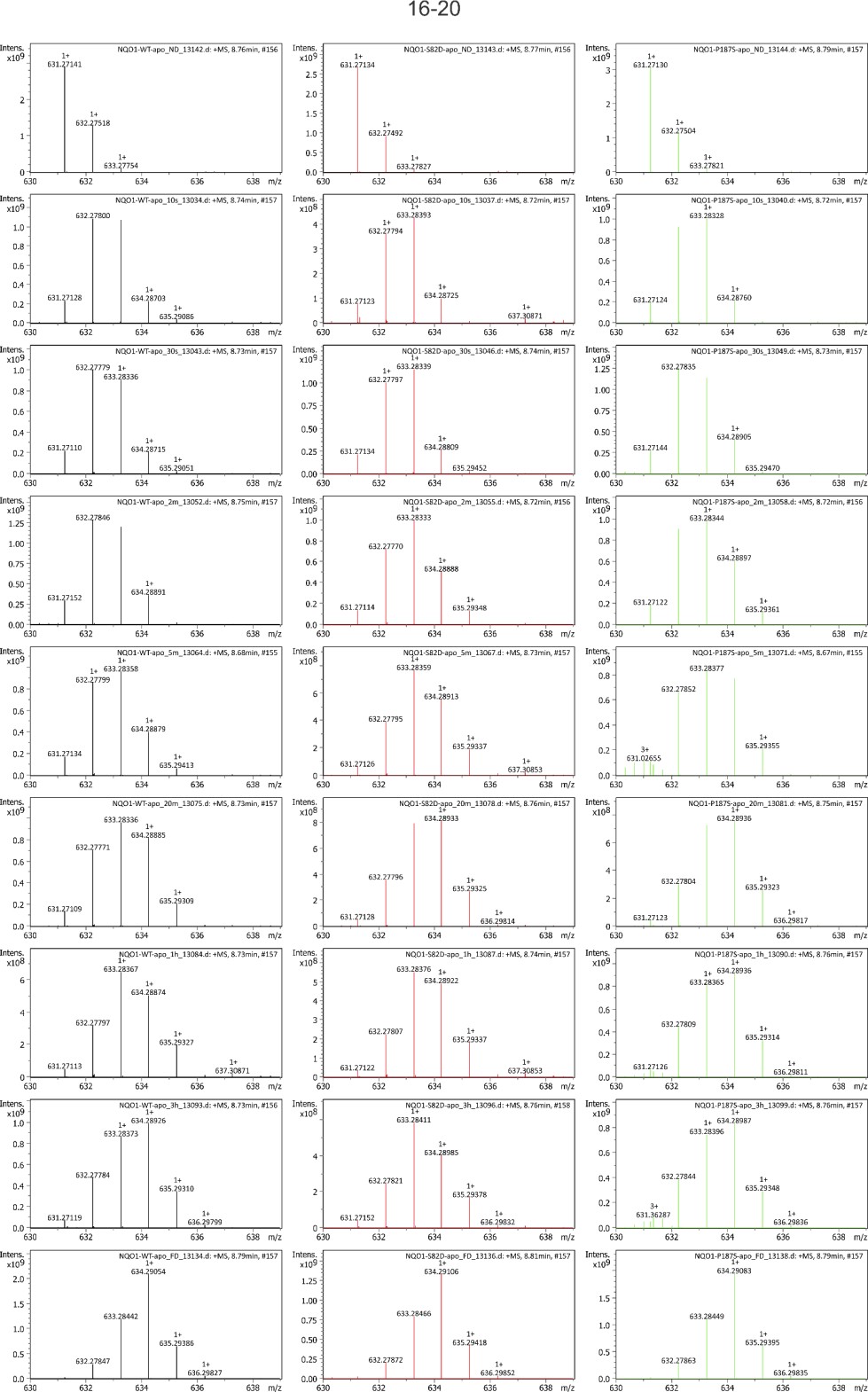


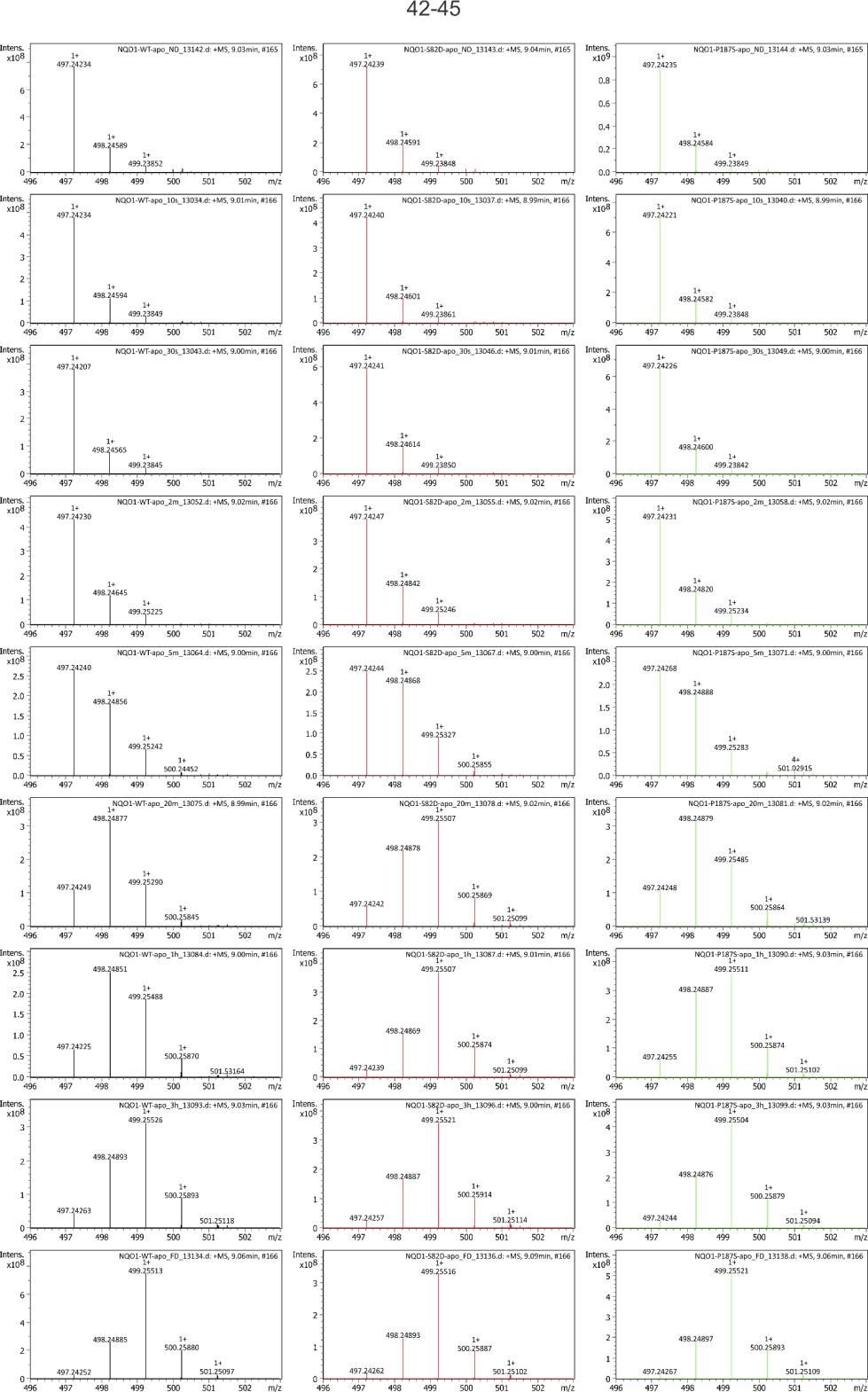


**S4B**

**S4C**


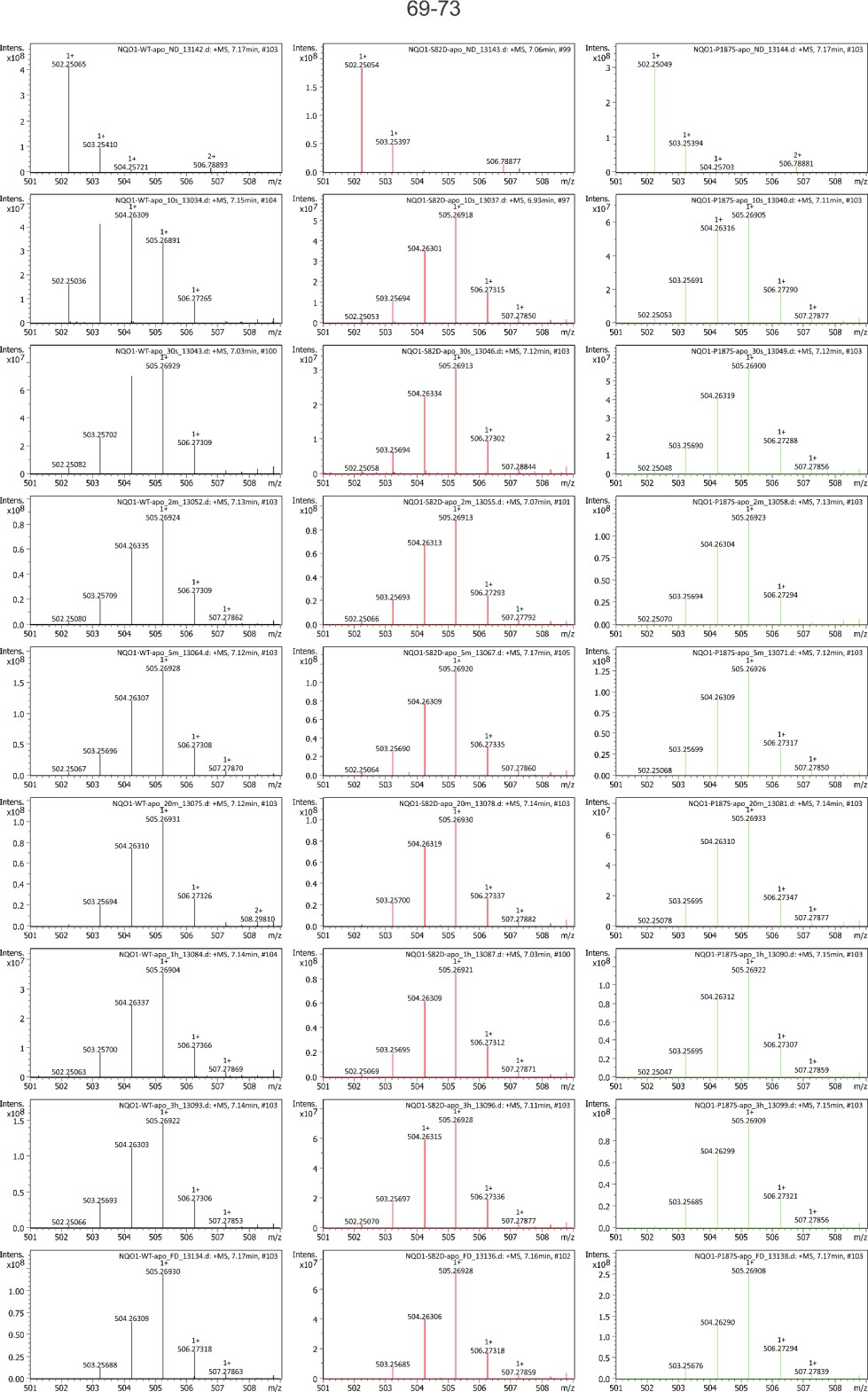


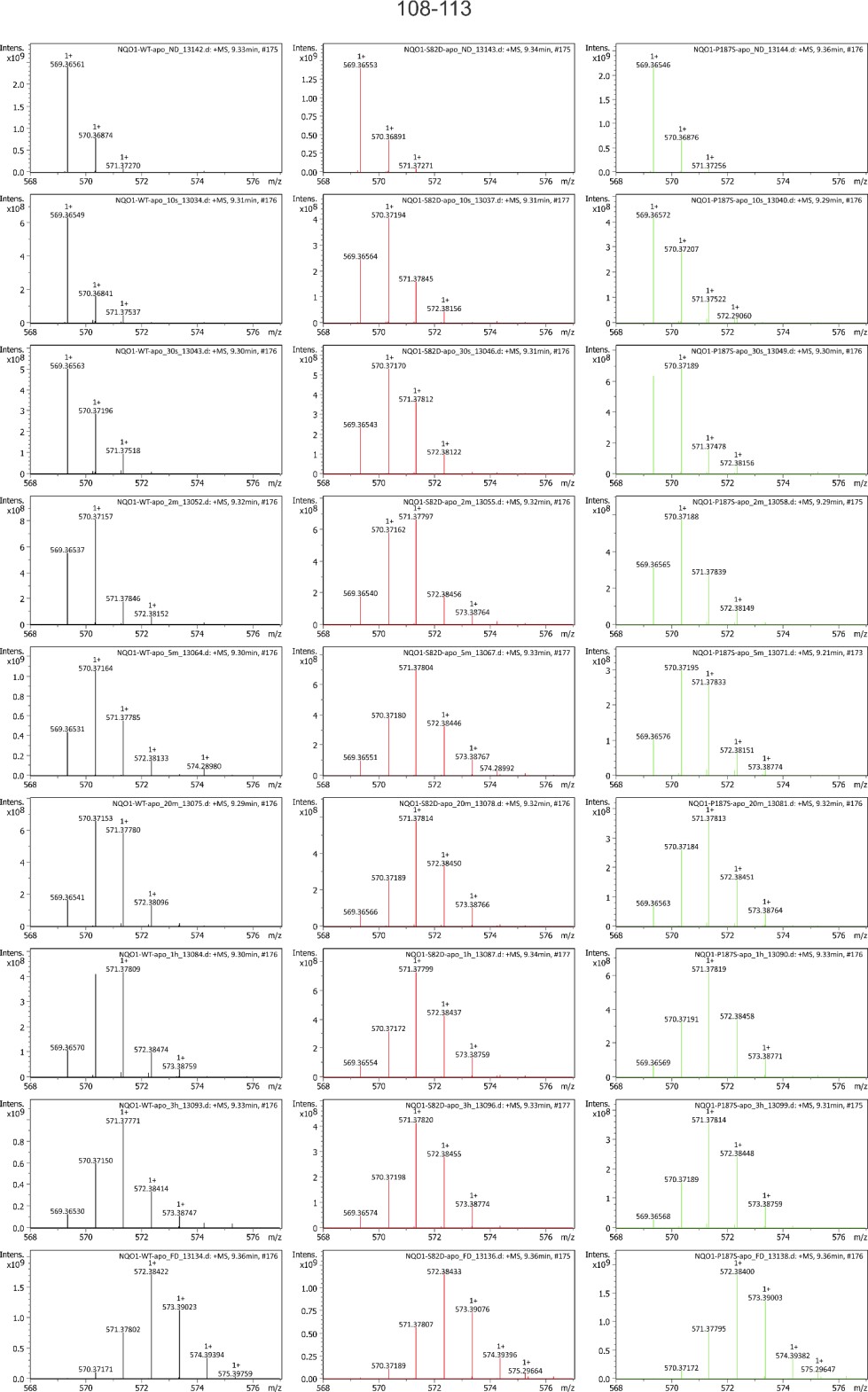
**S4D**

**S4E**


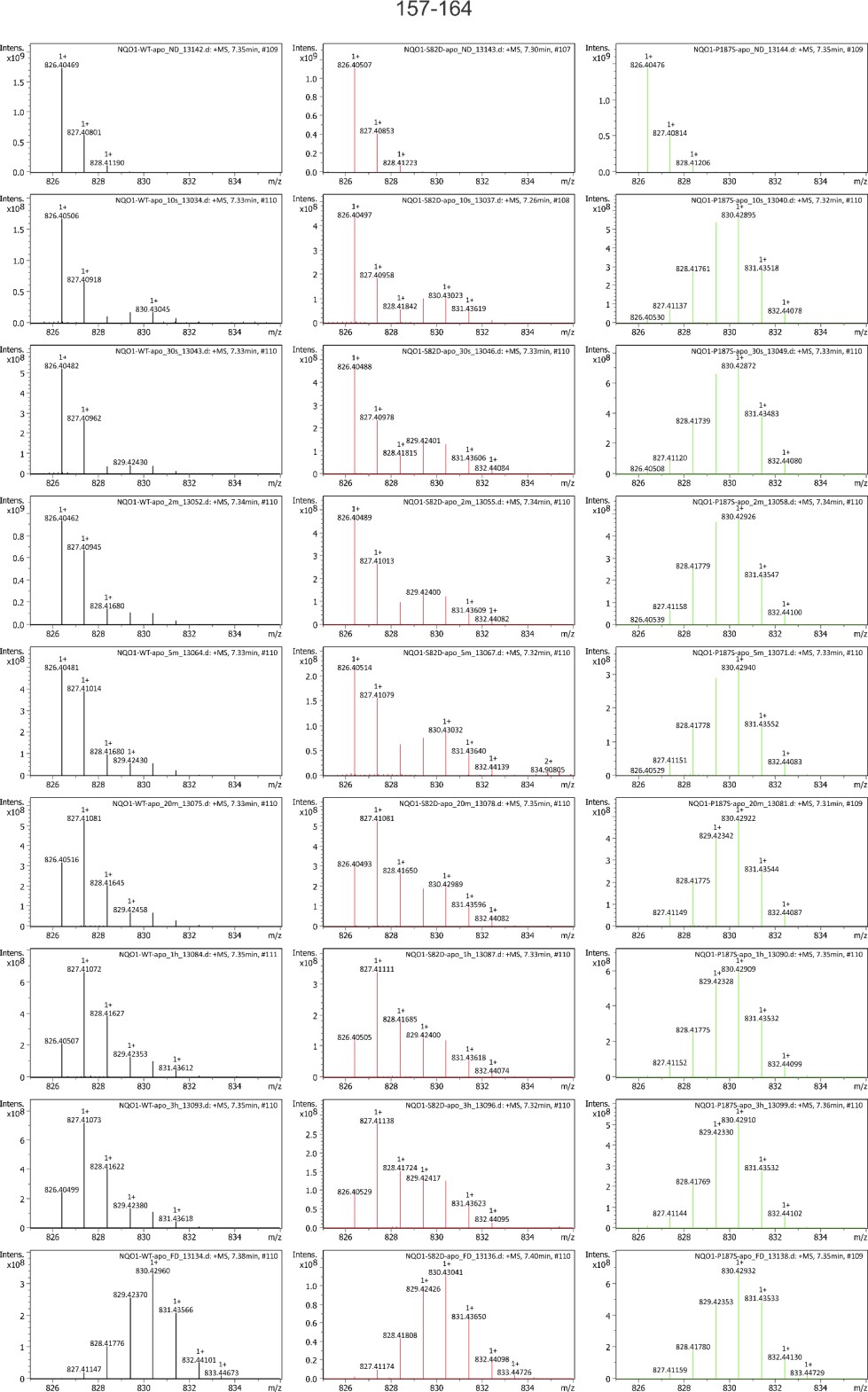


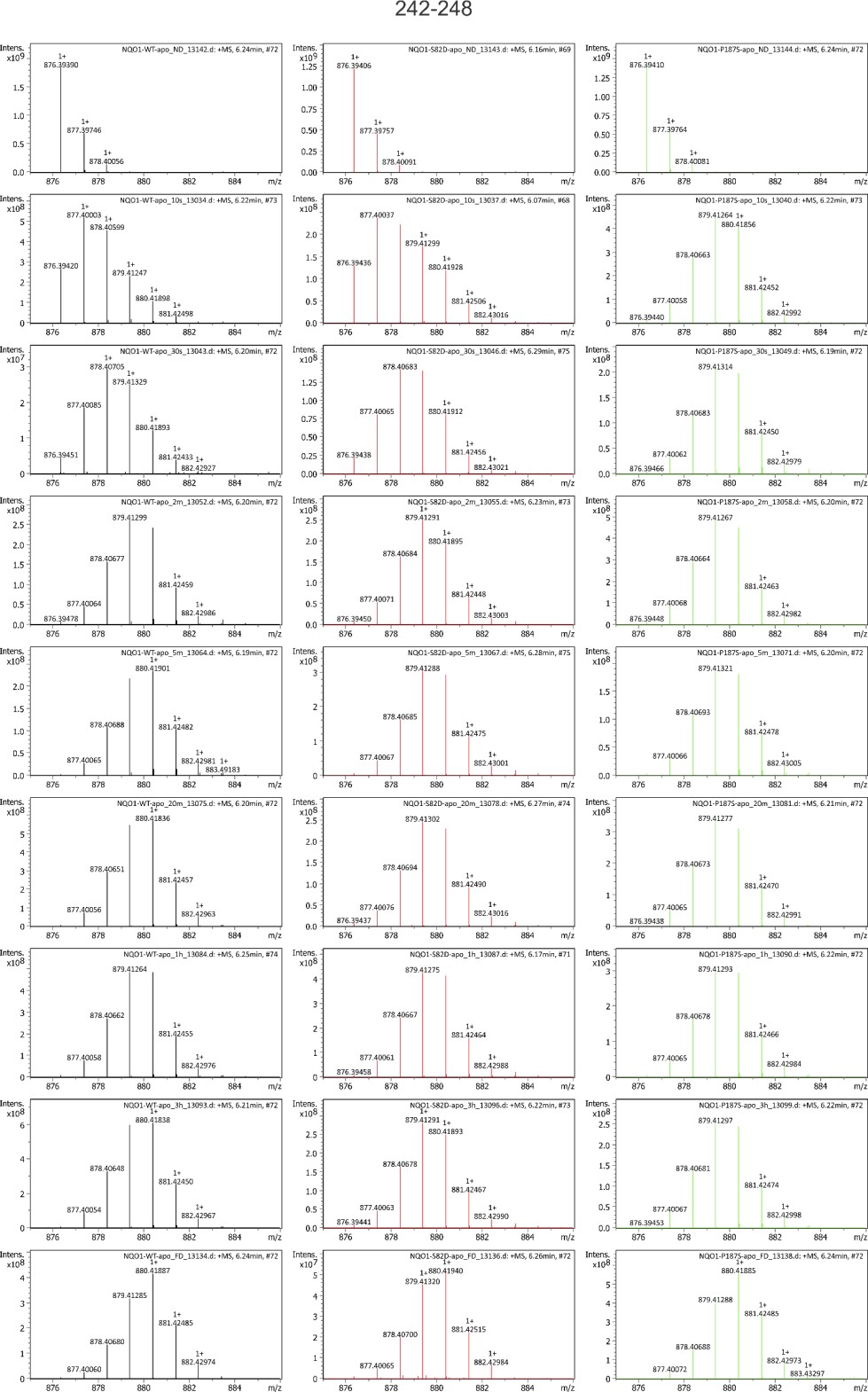
**S4F**

**Figure S5.** Deuterium uptake plots for all detected peptides of **NQO1_holo_** state. Wild-type (**black**), S82D (**red**) and P187S (**green**).

**
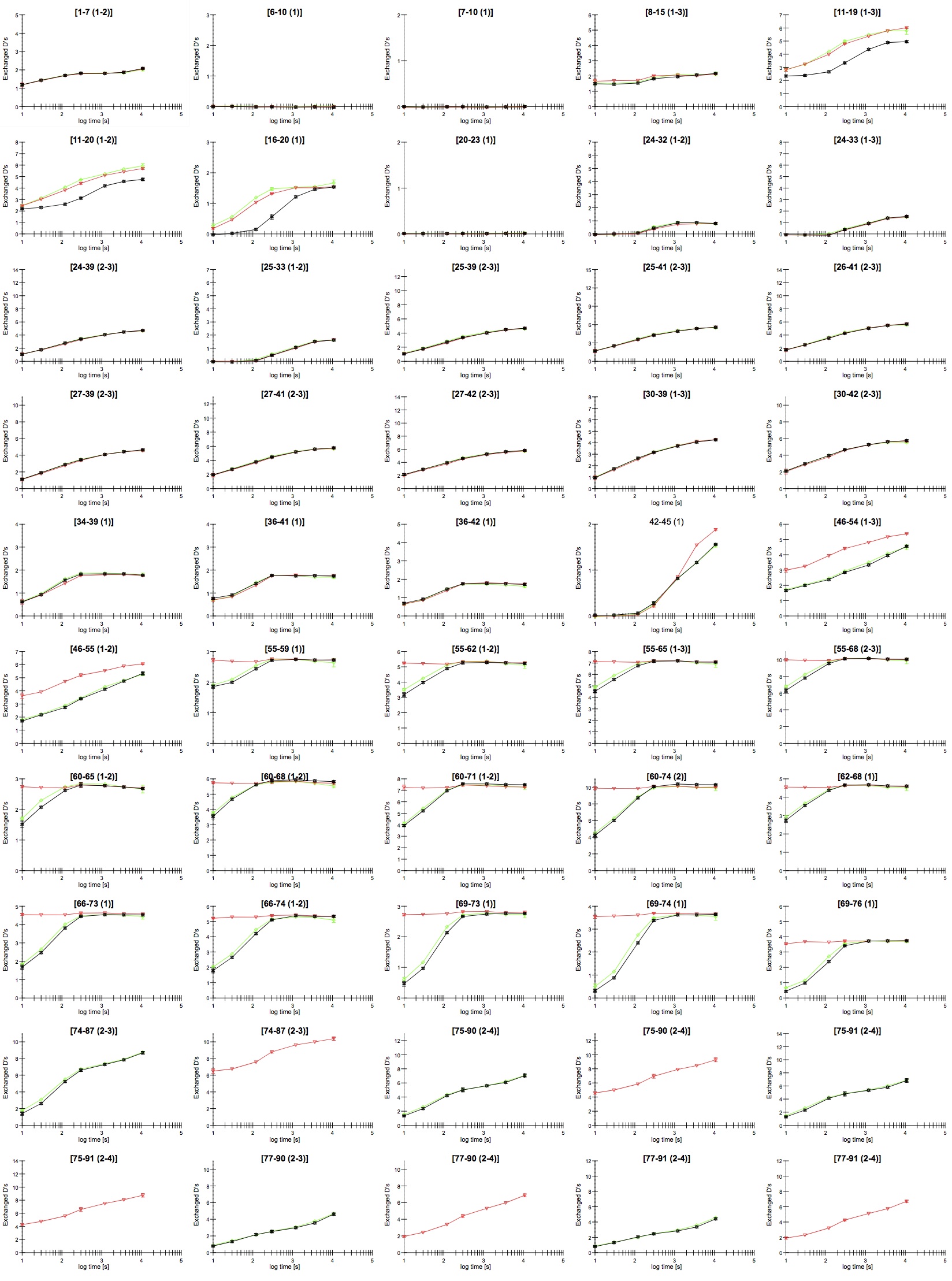
**

**
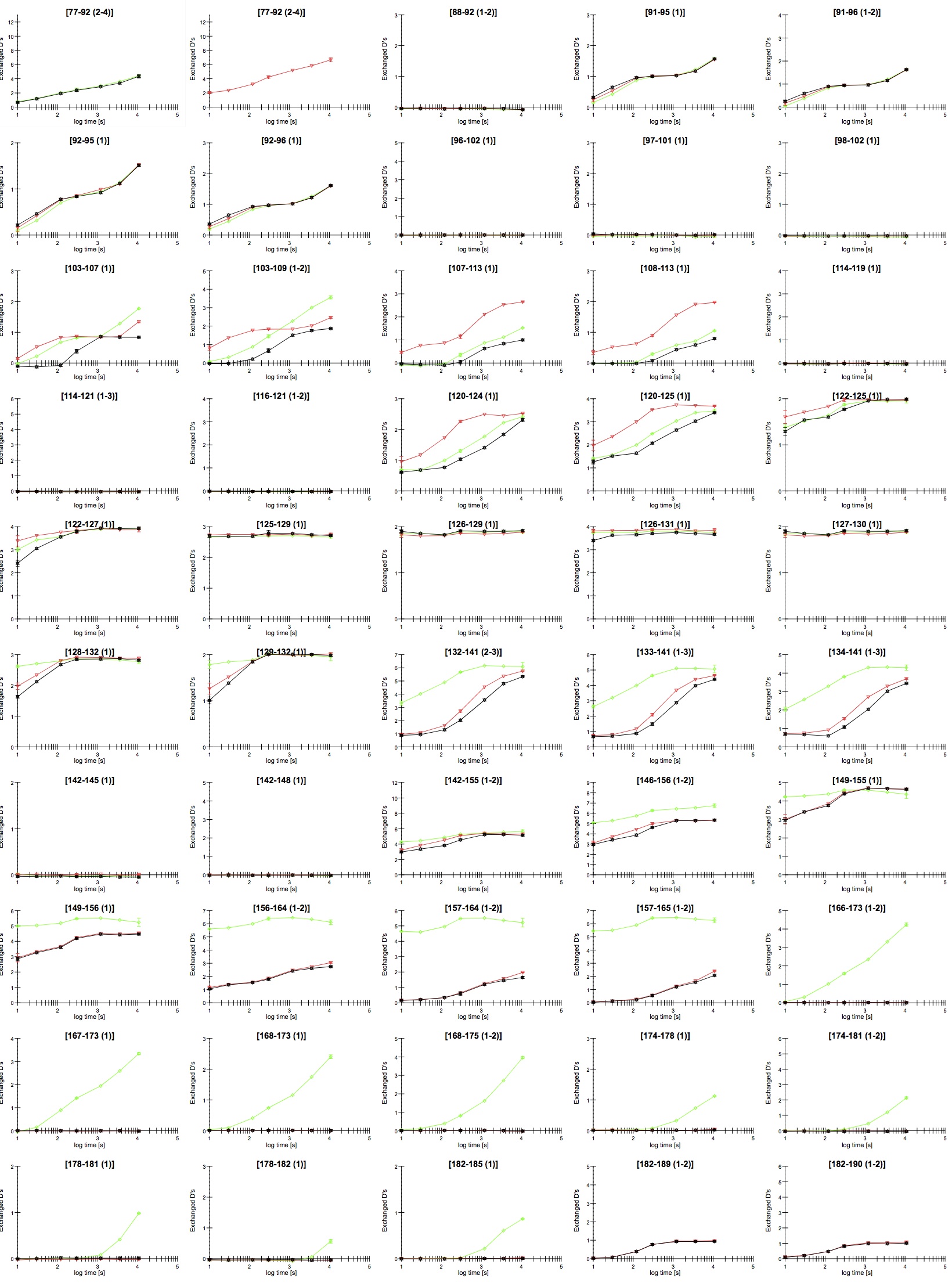
**


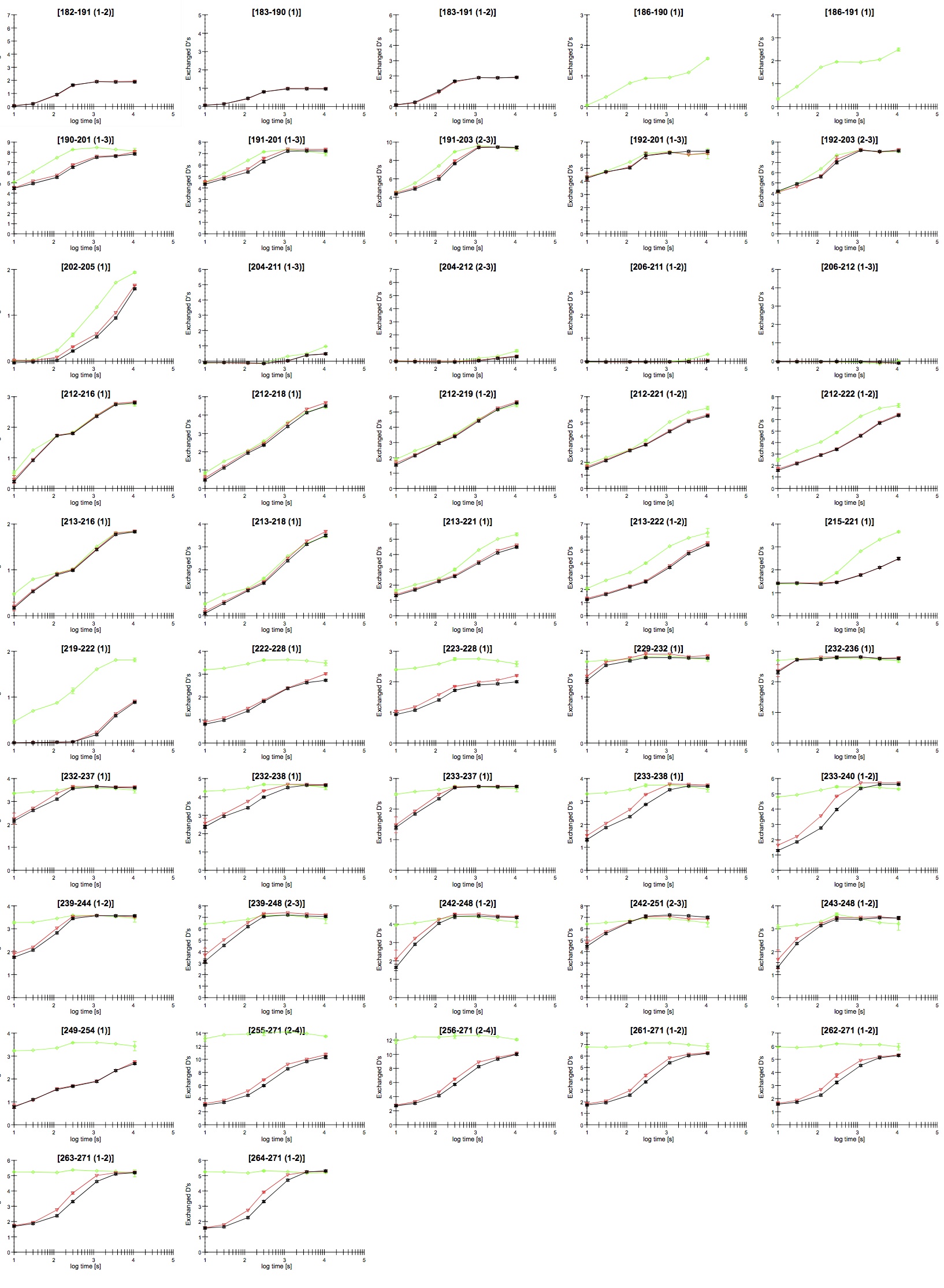


**Figure S6. Deuterium uptake plots for all detected peptides of NQO1_dic_ state. Wild-type (black), S82D (red) and P187S (green).**

**
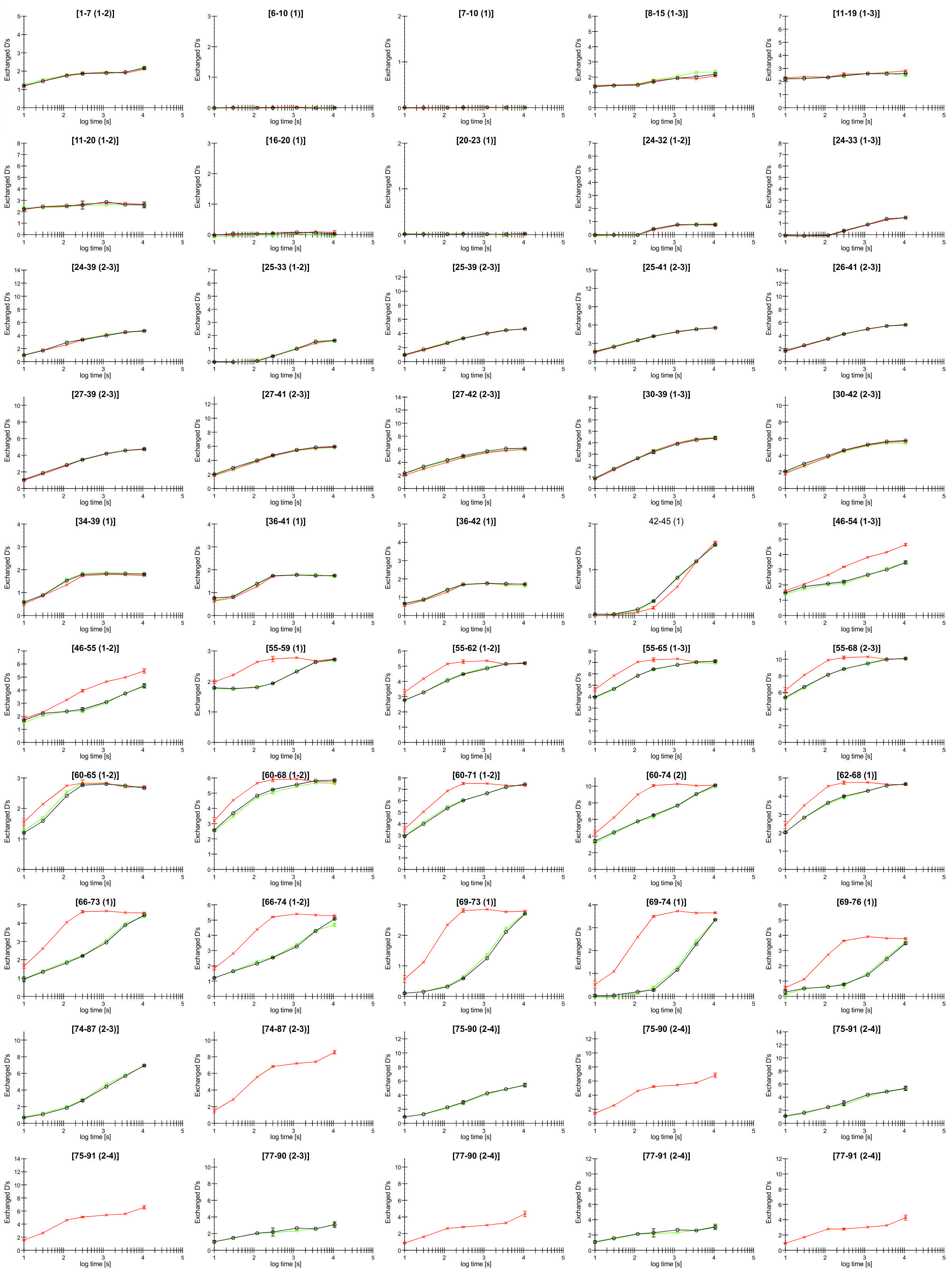
**

**
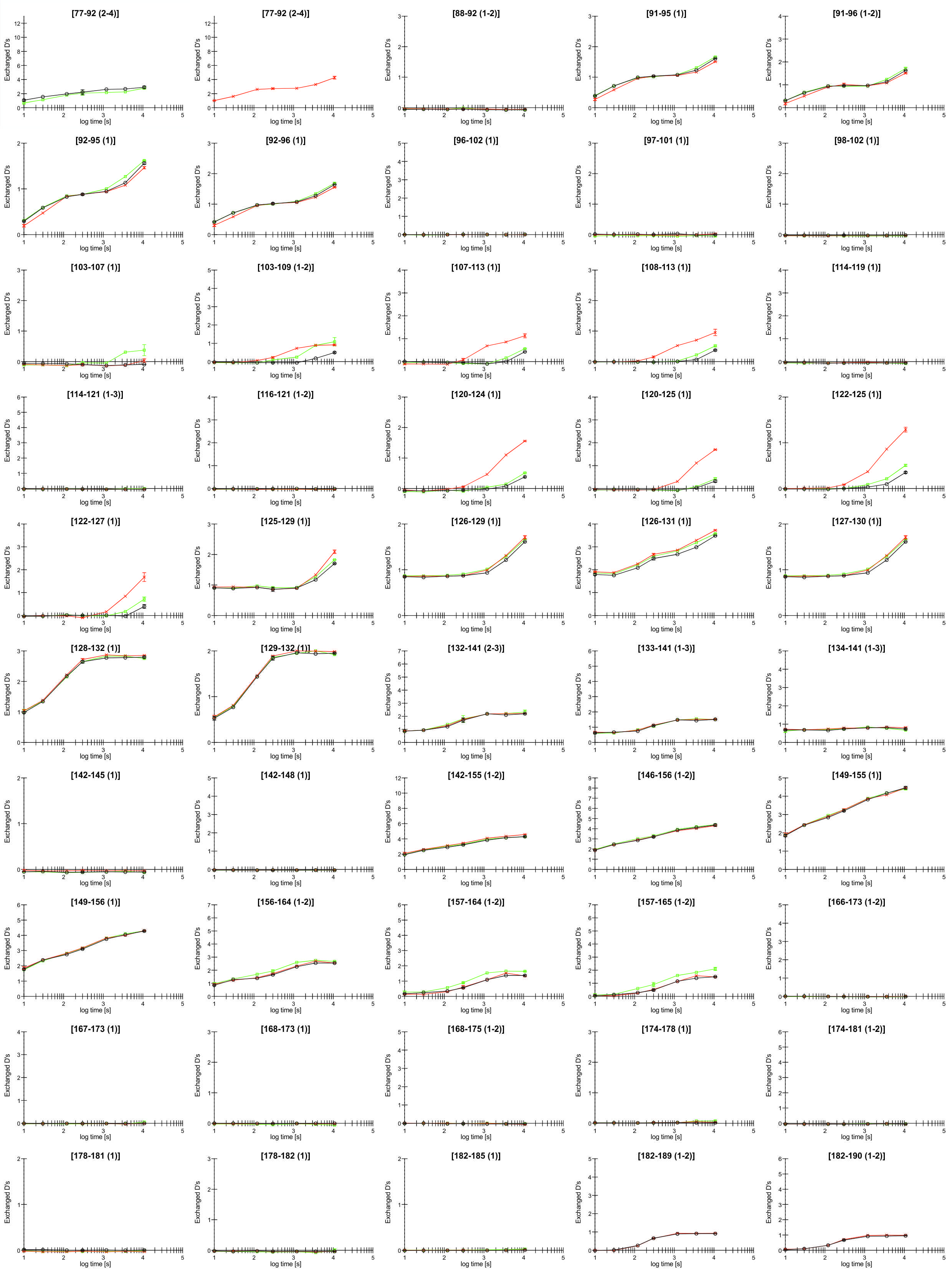
**

**
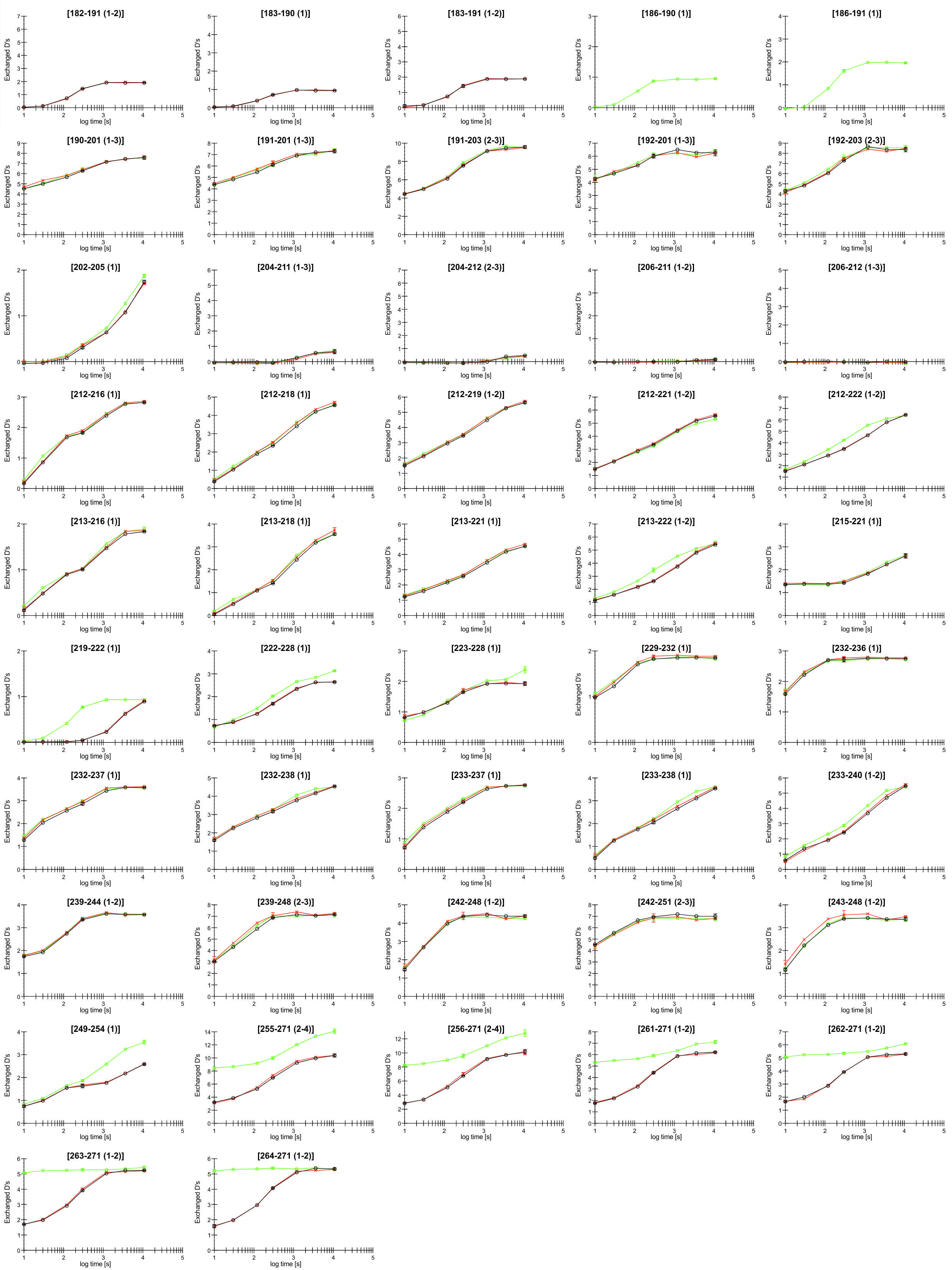
**

**Figure S7. Free energy calculations of the conformational ensemble of NQO1_apo_ WT.** A) Plot of the one-dimensional free energy profile as a function of the number of structured blocks (the reaction coordinate, RC) calculated at pH 7.0 and 298 K. At least two intermediate states (I_1_ and I_2_) apart from the native state (N) were identified. B) Representation of different combination of blocks (i.e. segments) structured at different points on the folding free energy profile (colored from 0 to 1 and in blue to red, with red representing fully folded). Intermediates can be seen by the vertical lines spanning the two panels.

**
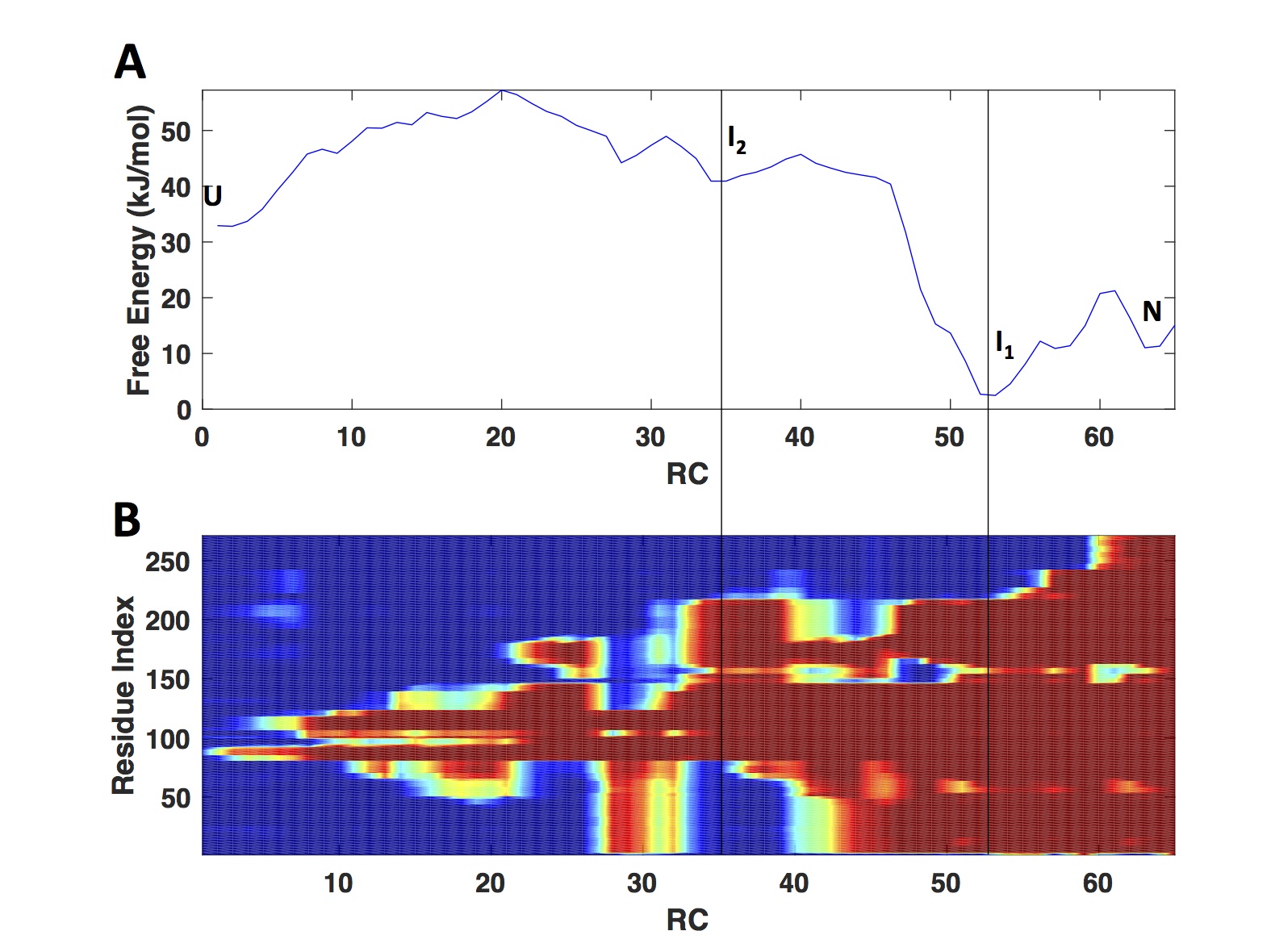
**

**Figure S8. Overall HDX kinetics for 105 segments of NQO1_apo_ variants.** The color scale indicates different times of exchange. Data for WT_apo_ are from [[6](#_ENREF_6)].

**
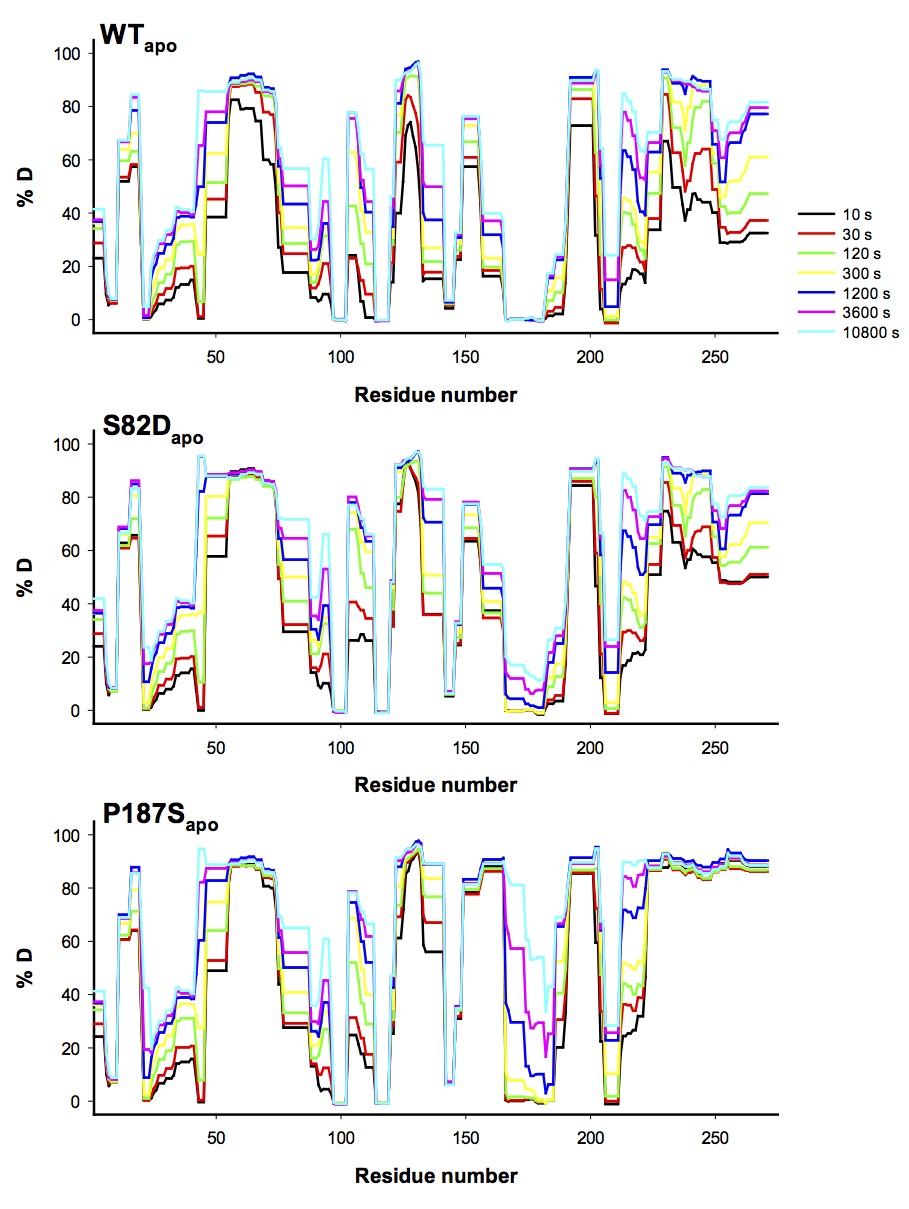
**

**Figure S9. Overall HDX kinetics for 105 segments of NQO1_holo_variants.** The color scale indicates different times of exchange. Data for WT_holo_ are from [[6](#_ENREF_6)].

**
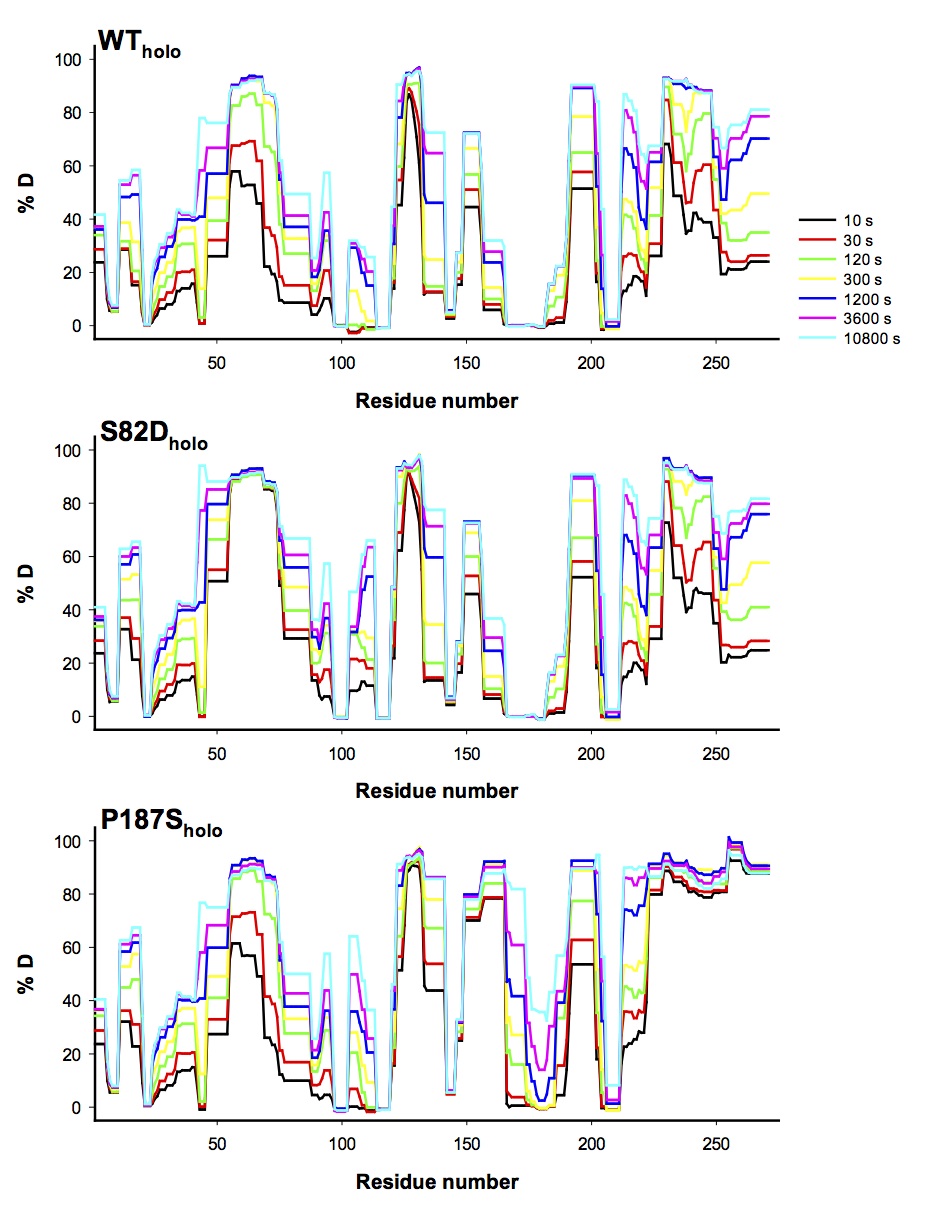
**

**Figure S10. Changes in HDX kinetics due to the S82D and P187S mutations on different ligation states.** Data are shown as Δ%D_av_ for different protein segments using the WT protein as reference [[6](#_ENREF_6)].

**
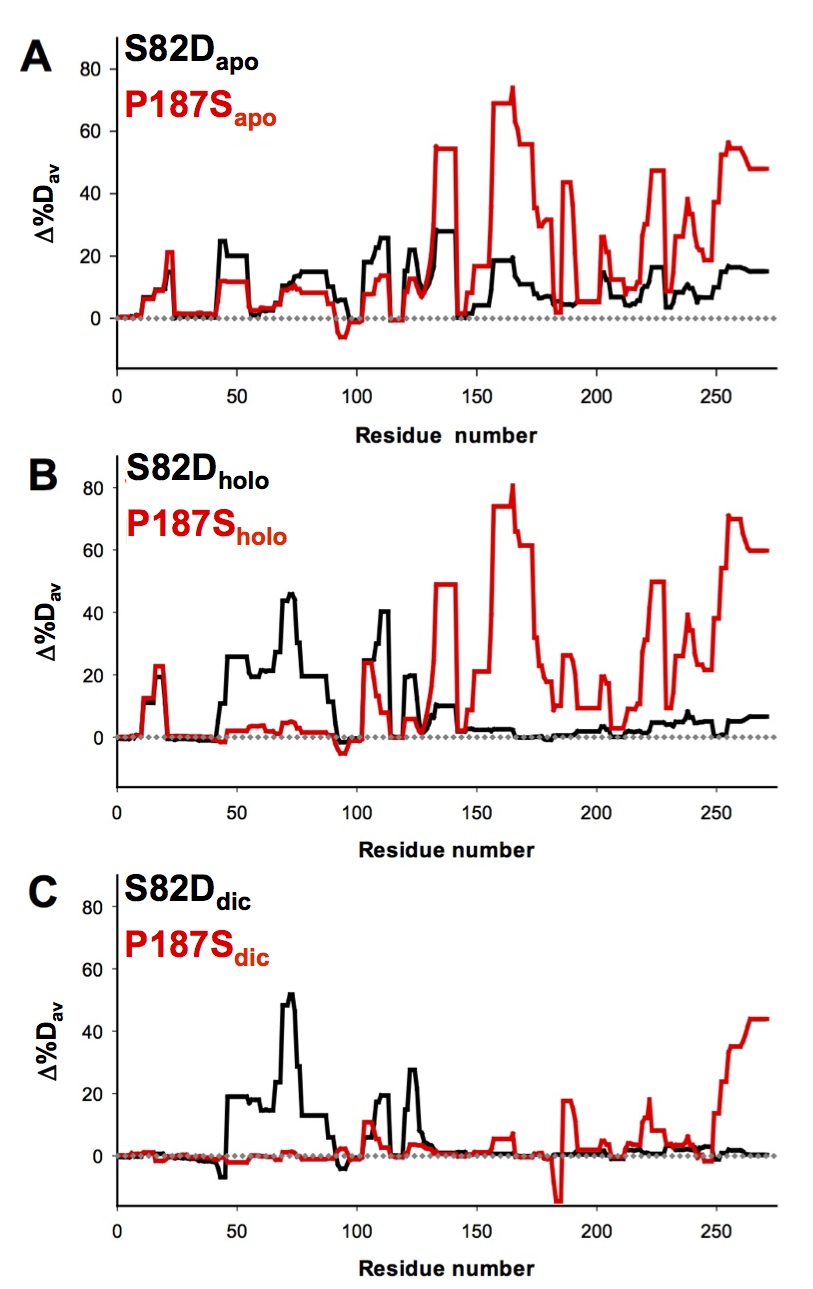
**

**Figure S11. Effects of S82D and P187S on the structural stability of the MMI in NQO1_holo_ and NQO1_apo_.** The plots in grey scale show the location of S82 and P187 (as black sphere representation) and the FAD molecule (as black stick representation), whereas those in color scale show the residues with values of Δ%D_av_ ≥ 10%. Two different views rotated by 90º are shown.


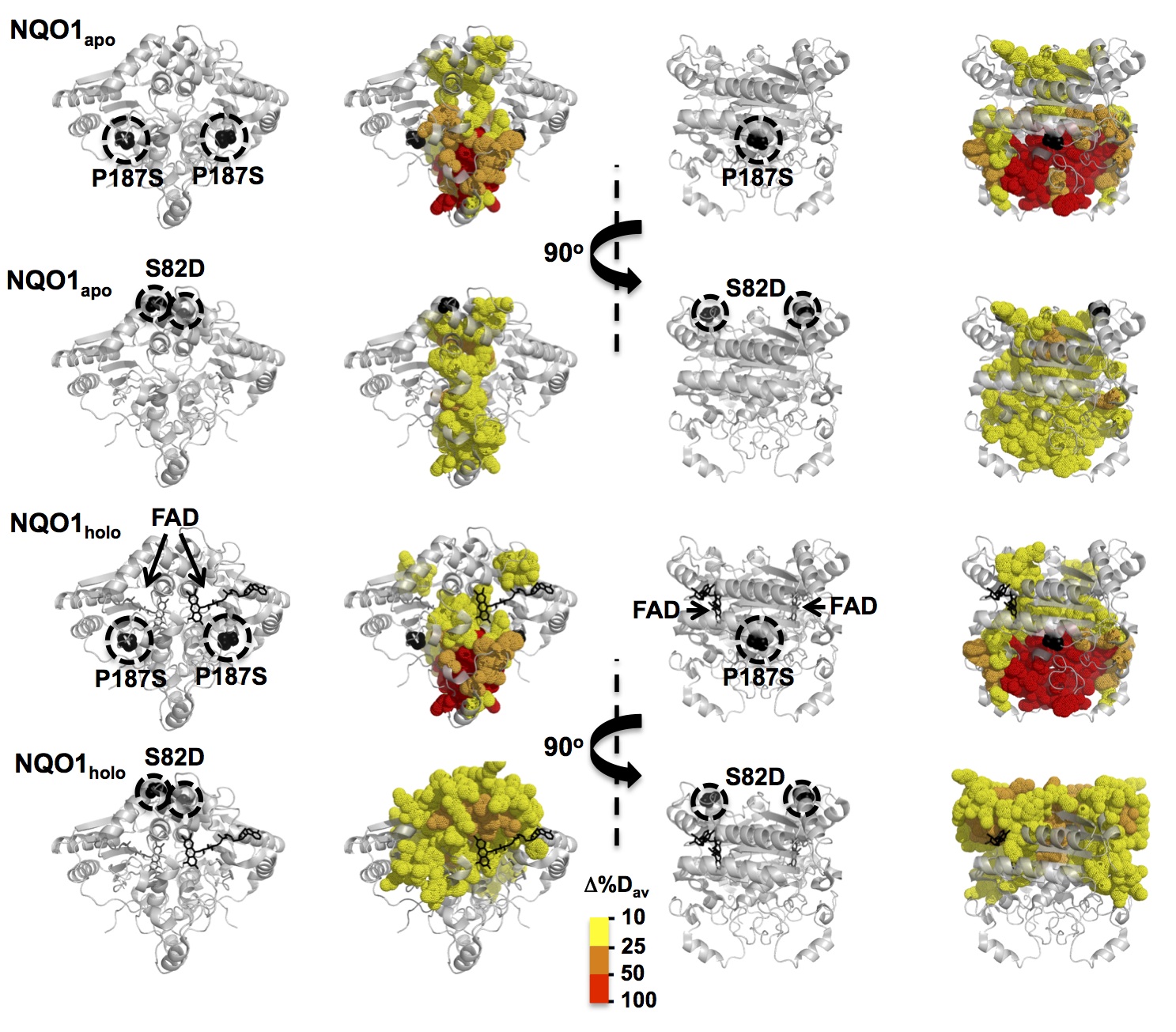


**Figure S12. Overall HDX kinetics for 105 segments of NQO1_dic_ variants.** The color scale indicates different times of exchange. Data for WT_dic_ are from [[6](#_ENREF_6)].

**
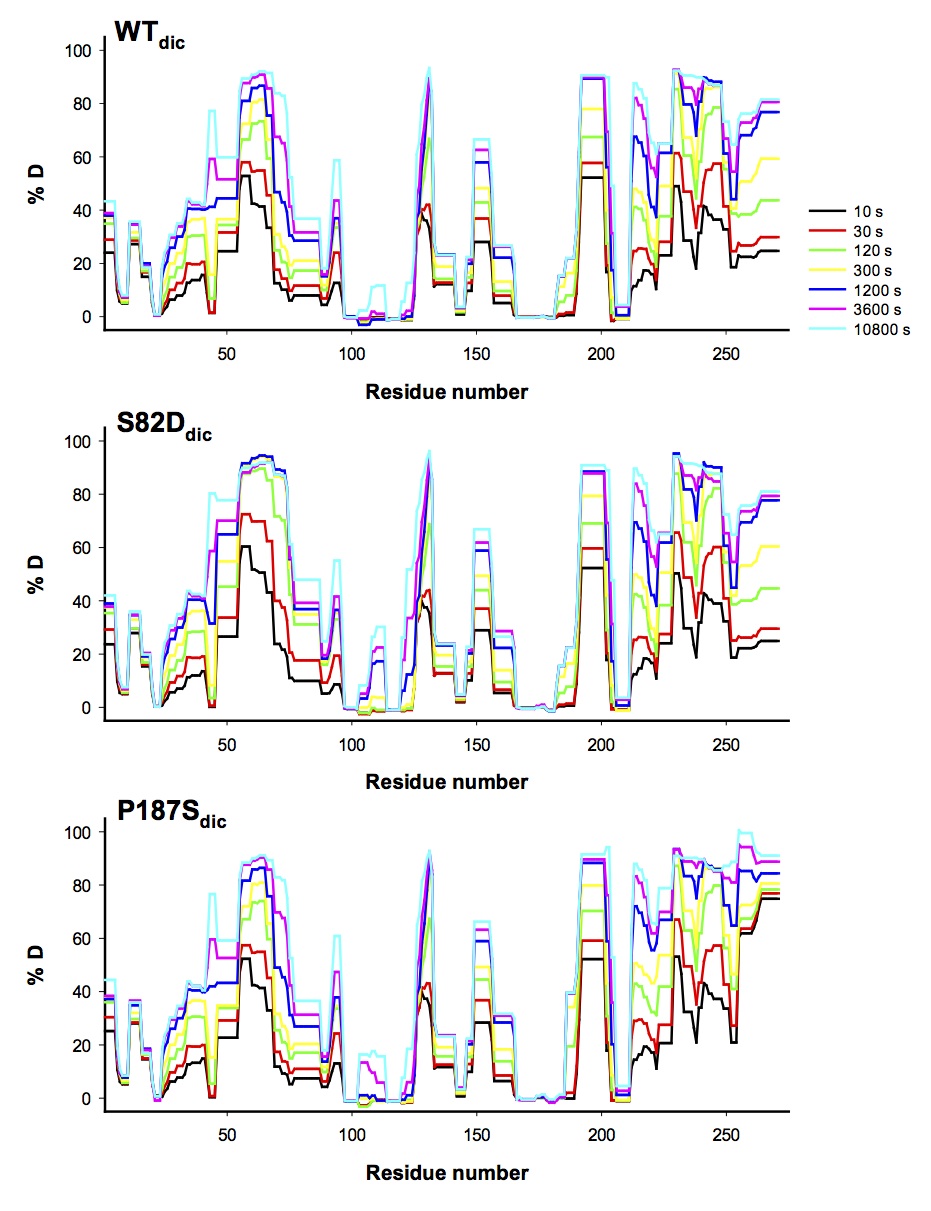
**

**Figure S13. Effects of S82D and P187S on the structural stability of the MMI in NQO1_holo_ and NQO1_dic_.** The plots in grey scale show the location of S82 and P187 (as black sphere representation) and the FAD and Dic molecules (as black stick representation), whereas those in color scale show the residues with values of Δ%D_av_ ≥ 10%. Two different views rotated by 90º are shown.

**
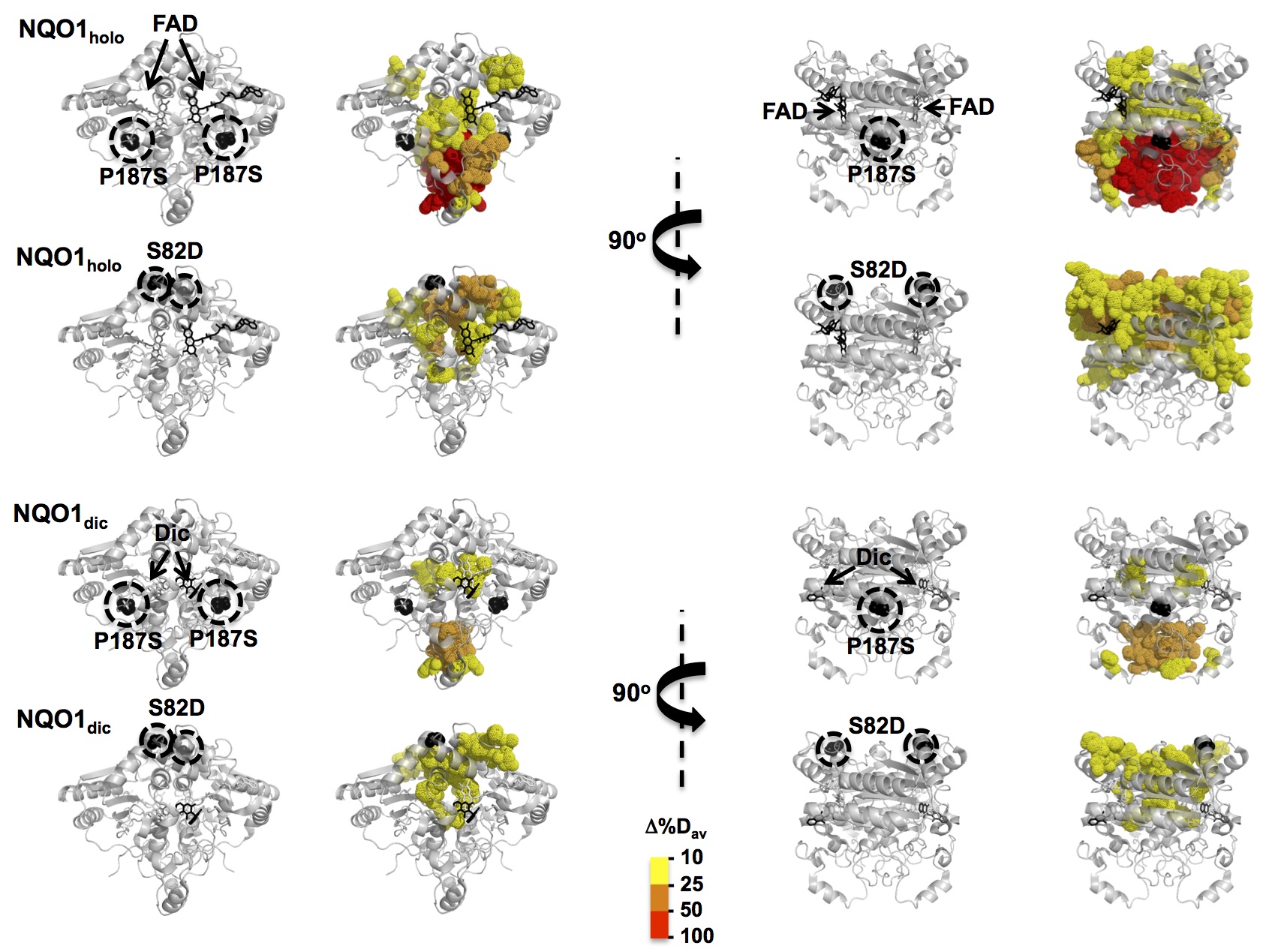
**

**Figure S14. Reductive half-reaction of FAD bound to NQO1 variants with NADH.** A) Time-dependent spectral evolution. B) Spectral deconvolution from a three-step model (A→B→C→D). C) Kinetic traces for the absorption at 450 and 475 nm (thin dashed lines) and fittings to a three-step model (thick solid lines). The upper panels show the residuals corresponding to fittings using data at 450 nm. Data correspond to NQO1 and NADH both at 7.5 μM. Temperature was 6^o^C. Data for WT NQO1 are from [[7](#_ENREF_7)].

**
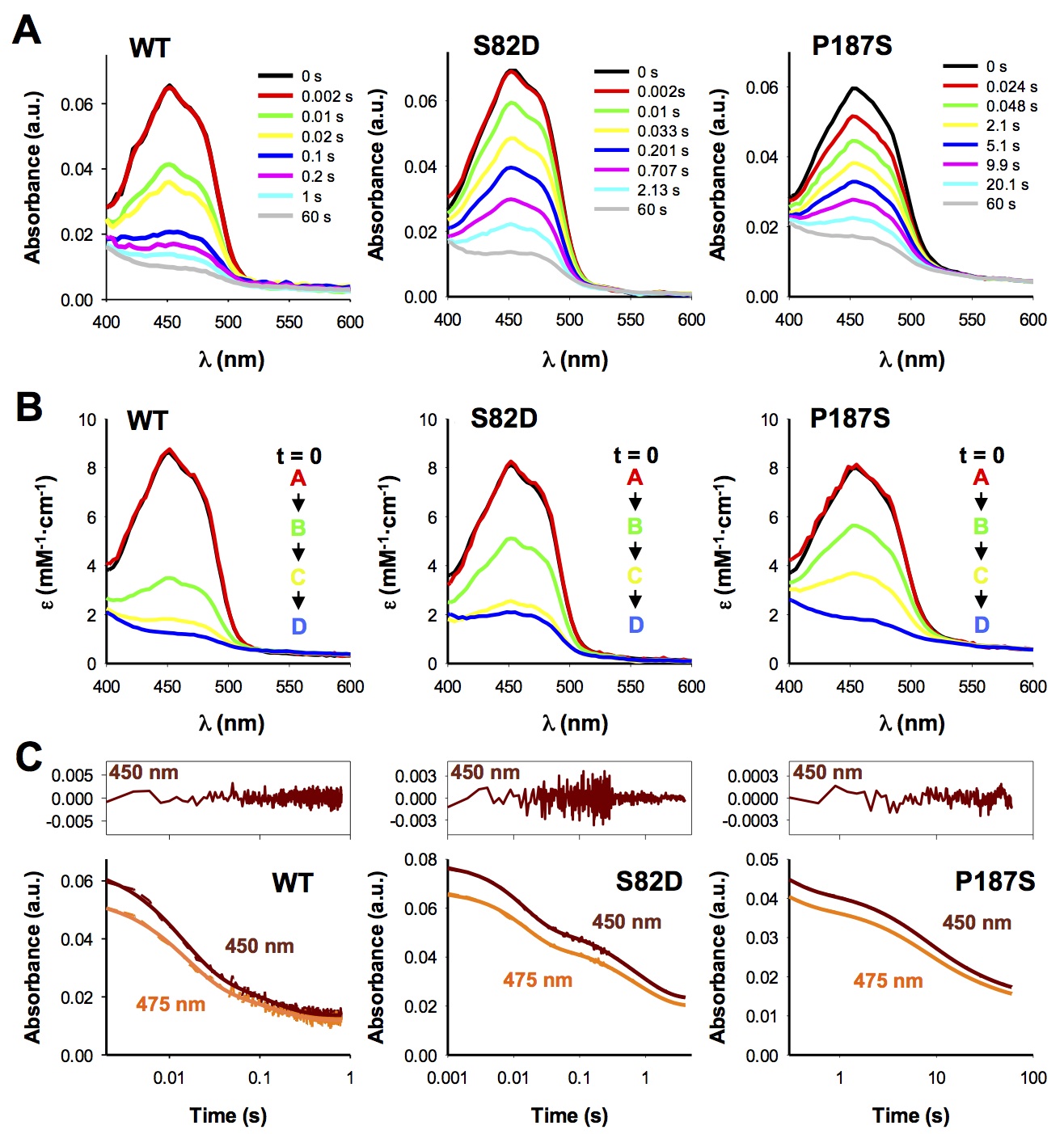
**

**Figure S15. Reductive half-reaction of FAD bound to NQO1 variants with NADPH.** A) Time-dependent spectral evolution. B) Spectral deconvolution from a two-step (A→B→C) or three-step model (A→B→C→D). When observed, the C→D step accounted for a very slow spectroscopic change with small amplitude, likely reflecting a process of little catalytic relevance. C) Amplitudes and observed rate constants. The right panel shows the fraction corresponding to the main processes (fast, A→B, and slow, B→C) determined as the fractional change in absorbance (average of 445-455 nm) corresponding to the spectral deconvolution (see panel B). The left panel shows the observed rate constant (*k*_obs_) obtained from kinetic analysis (see Panel D). Kinetic traces for the absorption at 450 and 475 nm (thin dashed lines) and fittings to a two-step or three-step model (thick solid lines). The upper panels show the residuals corresponding to fittings using data at 450 nm. Data correspond to NQO1 and NADPH both at 7.5 μM. Temperature was 6^o^C.

**
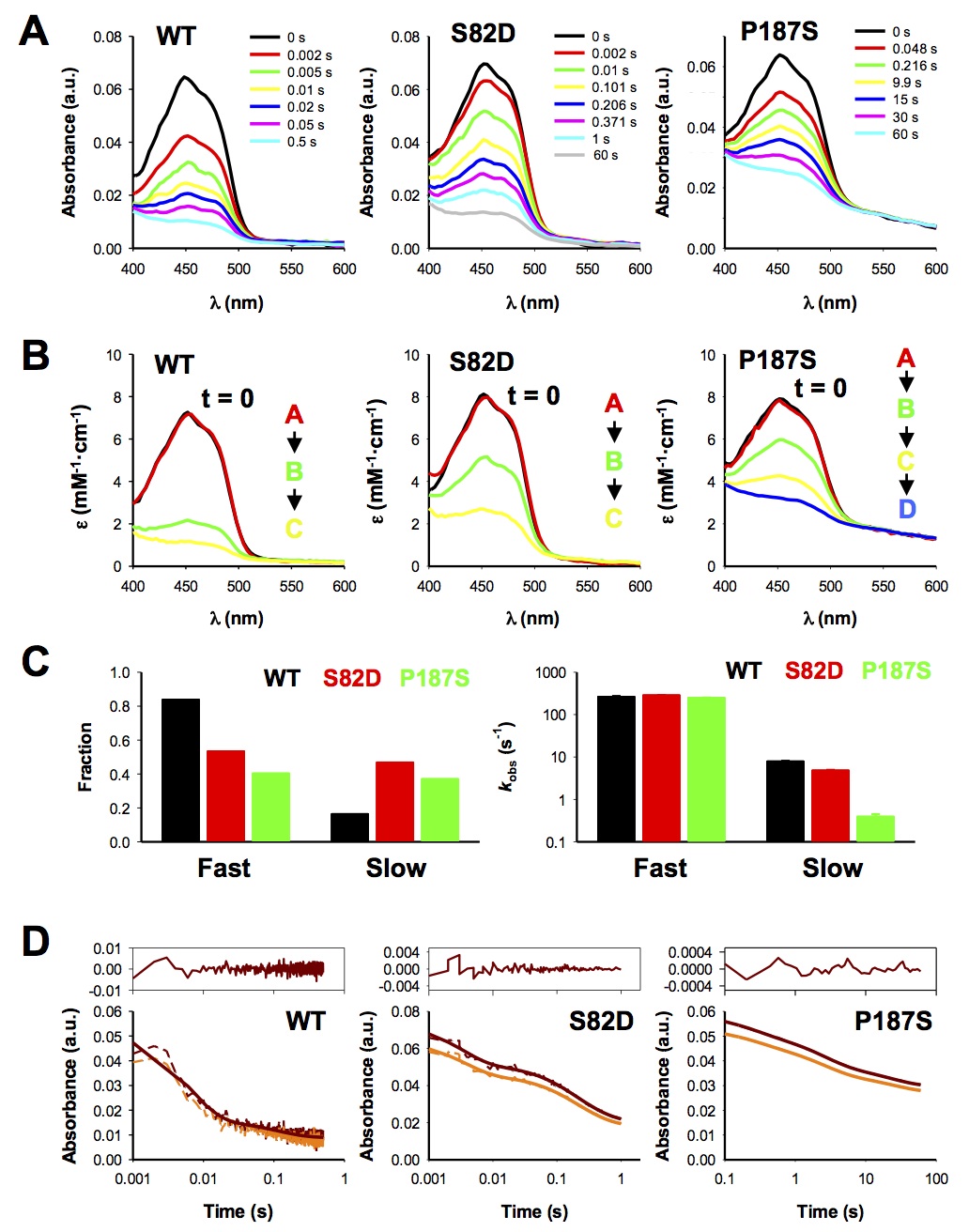
**

**Figure S16. Oxidative half-reaction of NQO1_hq_ variants with DCPIP.** A) Time-dependent spectral evolution. B) Spectral deconvolution from a two-step model (A→B→C). C) Kinetic traces for the absorption at 450, 475 and 600 nm (thin dashed lines) and fittings to a two-step model (thick solid lines). The upper panels show the residuals corresponding to fittings using data at 450 nm. Data correspond to NQO1_hq_ and DCPIP both at 7.5 μM. Temperature was 6^o^C.

**
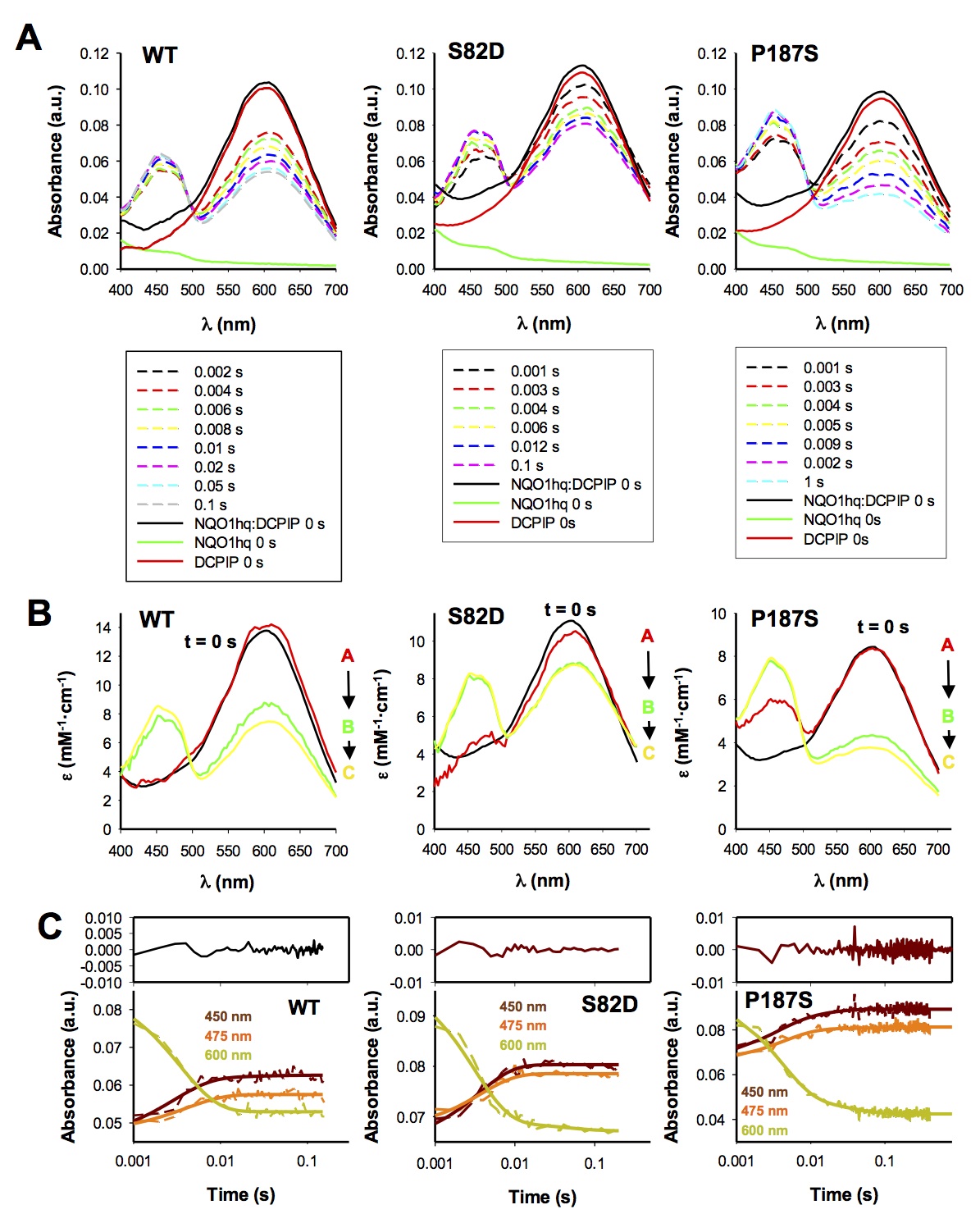
**

**Figure S17. Representative SDS-PAGE gels for proteolysis kinetics with trypsin.** Each variant and ligation state is indicated, as well as the time point of experiments and protease concentration.


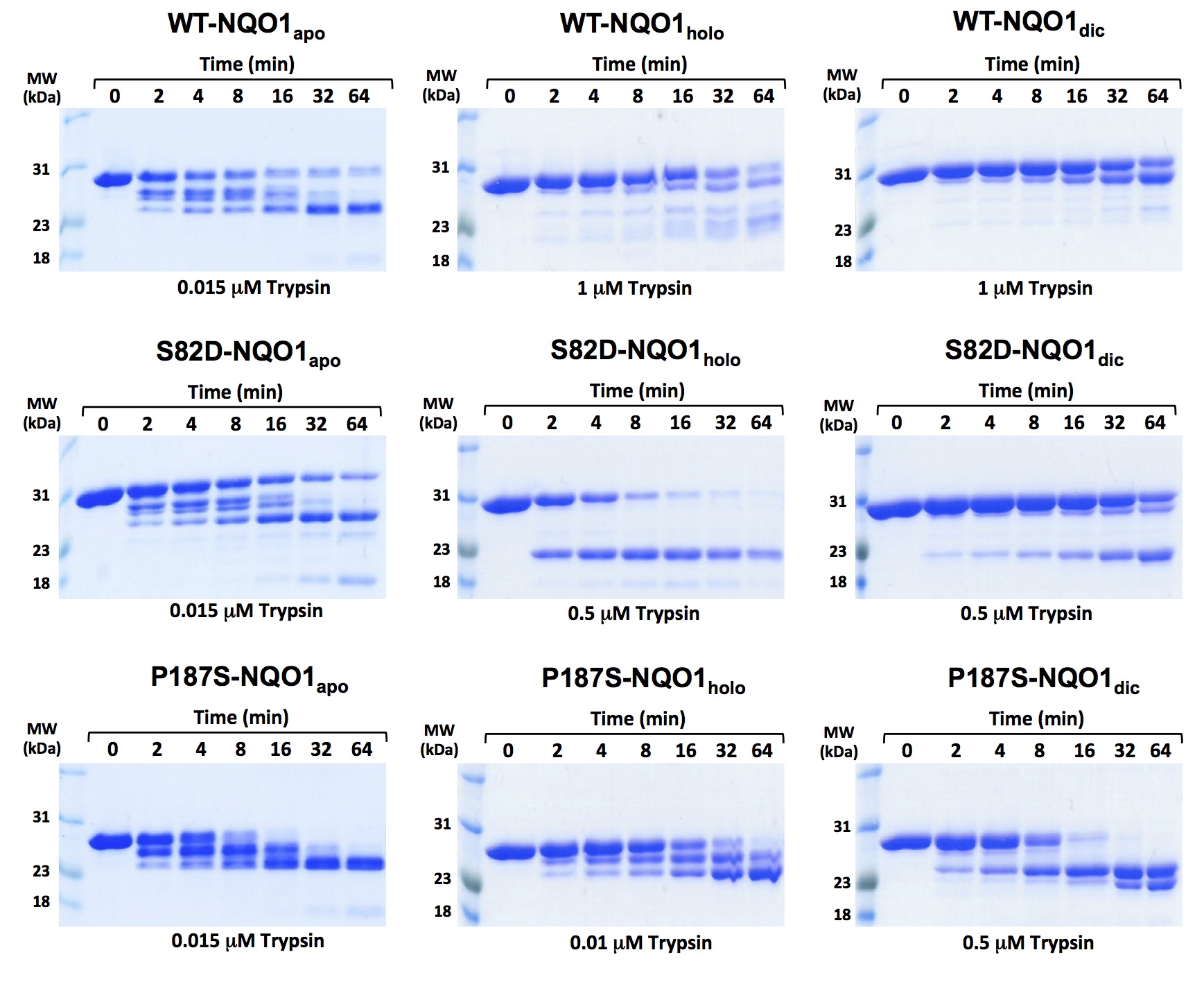


**Figure S18. Proteolysis kinetics by trypsin of NQO1 variants in different ligation states.** In different panels, the apparent first-order decays are shown at different protease concentrations for different variants and ligation states to provide *k*_obs_. From the linear dependence of *k*_obs_ on protease concentration (left row), the second-order rate constant is calculated.

**
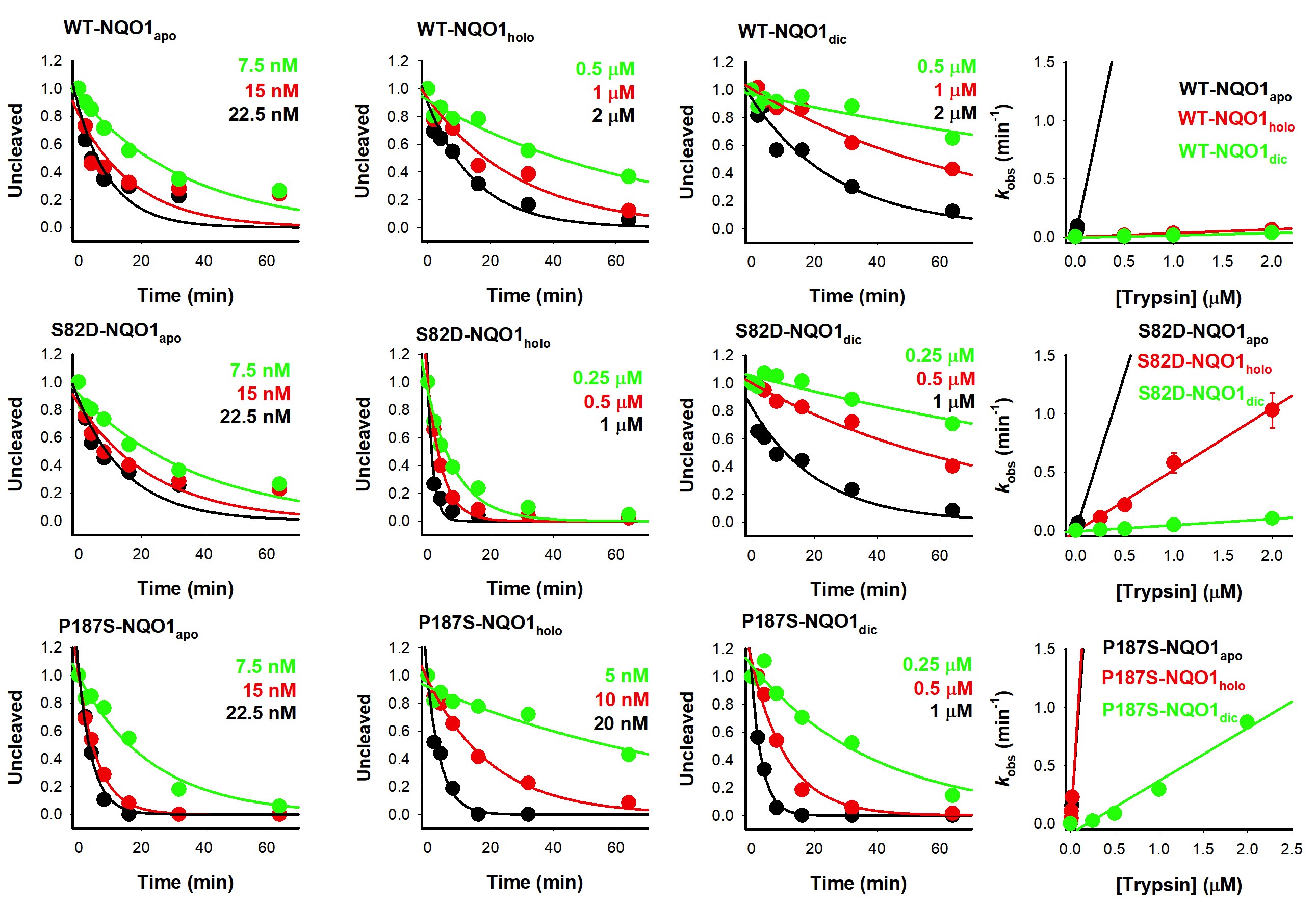
**

**Figure S19. Dic binding thermodynamics by ITC.** In A-C, left panels show representative titrations of NQO1 variants (15-17 μM in NQO1, 180 μM Dic; initial injection 0.5 μL, then 23 x 1.5 μL for WT and S82D or 18 x 2 μL in P187S; both protein and ligand with 100 μM FAD), whereas the right panels display representative binding isotherms at different temperatures. Panel D shows the temperature dependence of binding free energy, enthalpy and entropy. Data at 25^o^C are from 3-4 different titrations. Two protein batches from different purifications were used for each variant.


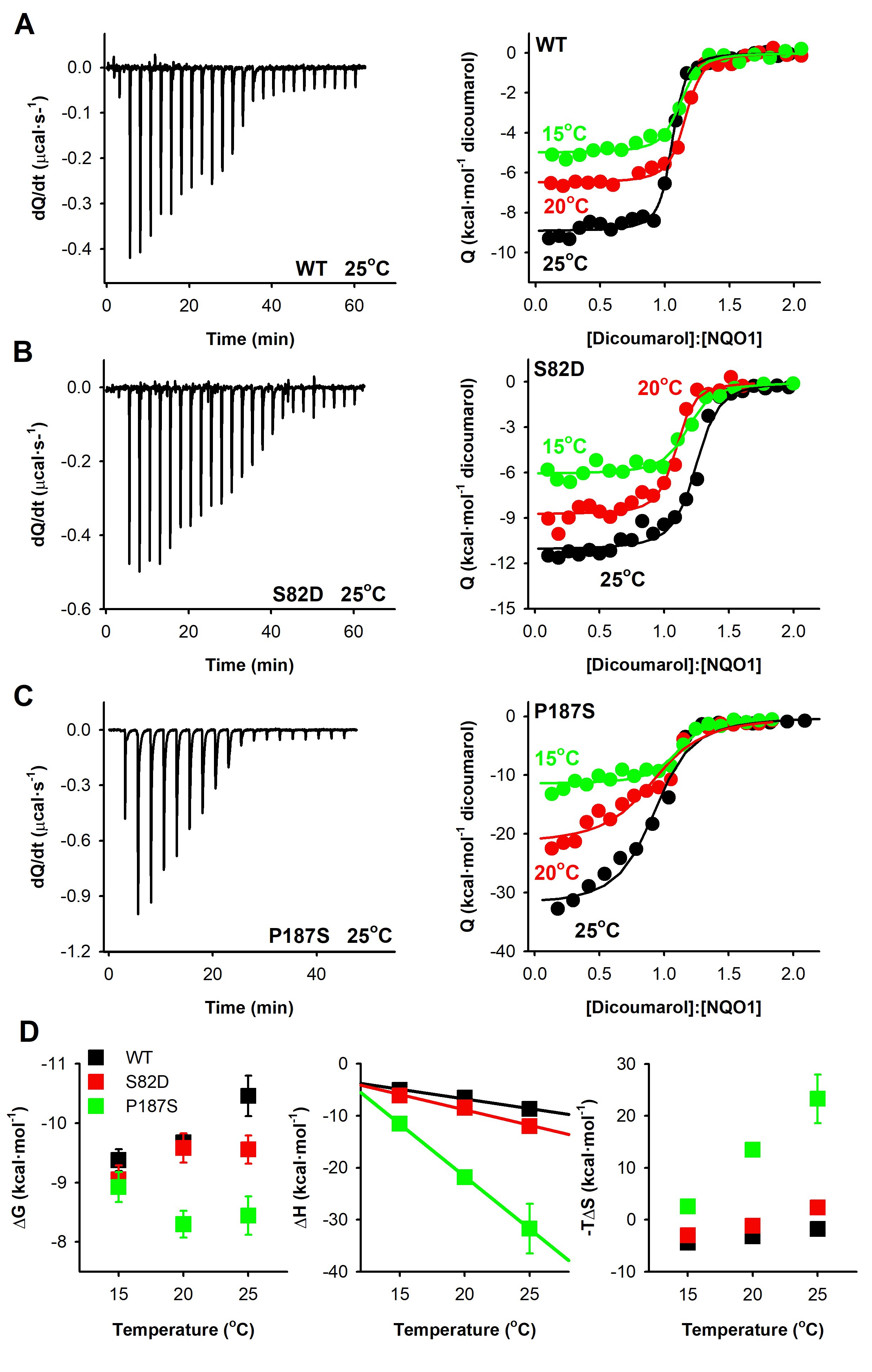


**Figure S20. The stability of S82D and P187S mutants is more sensitive to proteasomal degradation than that of WT NQO1.** Representative western-blot analysis of HAP-1 NQO1-KO cells transfected with NQO1 variants. Cells were treated for 4 h with the proteasomal inhibitor MG-132 and protein levels were determined by western-blot. Densitometric analysis of western-blots are indicated as mean ±s.d. from three 3 technical replicates and were normalized using WT NQO1 without treatment. Statistical analyses were carried out from one-tailed t-tests and significance reported as *p* values.


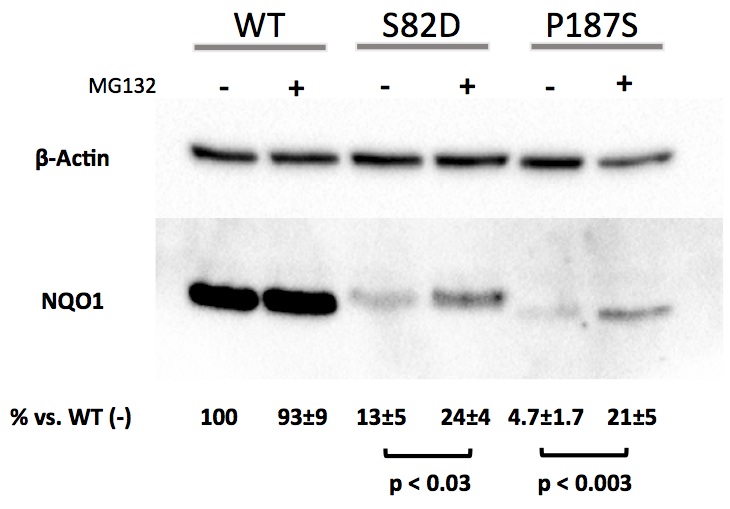


**Table S1.** **Human flavoproteins display multiple functional features.** Human flavoproteins (95) were searched according to the list provided by {Lienhart, 2013 #3986}. ***Function and catalytic activity*** was retrieved from the Uniprot server (<https://www.uniprot.org/>). For sake of simplicity, reactions are indicated only the forward direction. ***Protein:protein interactios (PPI)*** were retrieved from the BioGrid server (https://thebiogrid.org/). Only those interactions with at least two independent reports are included (note that the number of reports is indicated in parenthesis). 65% of the flavoproteins compiled in this table develop PPI. ***Subcellular locations*** were retrieved from the GeneCards server (<https://www.genecards.org/>). The confidence on the subcellular location for each protein is provided by this server a 1-5 scale (from the lowest to the highest confidence) and only those cases with a confidence ≥ 3 are included (note that the level of confidence is indicated in parenthesis. 78 % of the flavoproteins compiled are located in at least two different subcellular compartments. ***Structures for apo-proteins*** were retrieved (with their PDB code) from the Protein Data Bank in Europe (PDBe) server (<https://www.ebi.ac.uk/pdbe/>). Only those structures describing a full-length protein model without any bound ligand were compiled. Only 2% of the flavoproteins compiled in this table were found to have high-resolution crystal structure following this criteria . All data were retrieved between November 2020 and January 2021.

| **Name (Gene Name; UniProt code)** | **Function/Catalytic activity (UniProt)** | **PPI (BioGrid)** | **Subcellular location (GeneCards)** | **Apo-structures (PDBe)** |
| --- | --- | --- | --- | --- |
| 1. D-lactate dehydrogenase (LDHD; Q86WU2) | Involved in D-lactate, but not L-lactate catabolic process. /  (*R*)-lactate + 2 [Fe(III)cytochrome *c*] → 2 [Fe(II)cytochrome *c*] + 2 H^+^ + pyruvate  **EC:1.1.2.4** | None | Mitochondria (5) | None |
| 2. Xanthine dehydrogenase/oxidase (XDH; P47989) | Key enzyme in purine degradation. Catalyzes the oxidation of hypoxanthine to xanthine. Catalyzes the oxidation of xanthine to uric acid. Contributes to the generation of reactive oxygen species. Has also low oxidase activity towards aldehydes (in vitro)./  H_2_O + NAD^+^ + xanthine → H^+^ + NADH + urate  **EC:1.17.1.4;** H_2_O + hypoxanthine + NAD^+^ → H^+^ + NADH + xanthine; **EC:1.17.1.4**; H_2_O + O_2_ + xanthine → H_2_O_2_ + urate **EC:1.17.3.2** | None | Extracellular (5), peroxisome (4), cytosol (4), nucleus (3),  endoplasmic reticulum (3) | None |
| 3. Hydroxyacid oxidase 1 (HAO1; Q9UJM8) | Has 2-hydroxyacid oxidase activity. Most active on the 2-carbon substrate glycolate, but is also active on 2-hydroxy fatty acids, with high activity towards 2-hydroxy palmitate and 2-hydroxy octanoate./  (2*S*)-2-hydroxycarboxylate + O_2_ → 2-oxocarboxylate + H_2_O_2_ **EC:1.1.3.15** | None | Peroxisome (5), cytosol (5), nucleus (3) | None |
| 4. Hydroxyacid oxidase 2 (HAO2; Q9NYQ3) | Catalyzes the oxidation of L-alpha-hydroxy acids as well as, more slowly, that of L-alpha-amino acids./  (2*S*)-2-hydroxycarboxylate + O_2_ → 2-oxocarboxylate + H_2_O_2_ **EC:1.1.3.15** | None | Peroxisome (5), cytosol (4) | None |
| 5. Glycerol-3-phosphate dehydrogenase, mitochondrial (GPD2; P43304) | Calcium-responsive mitochondrial glycerol-3-phosphate dehydrogenase which seems to be a key component of the pancreatic beta-cell glucose-sensing device./  Quinone + *sn*-glycerol 3-phosphate → quinol + dihydroxyacetone phosphate **EC:1.1.5.3** | None | Mitochondria (5) | None |
| 6. Choline dehydrogenase, mitochondrial (CHDH; Q8NE62) | A + choline → AH_2_ + betaine aldehyde; **EC:1.1.99.1** | None | Mitochondria (5) | None |
| 7. L-2-hydroxyglutarate dehydrogenase, mitochondrial (L2HGDH; Q9H9P8) | (*S*)-2-hydroxyglutarate + A → 2-oxoglutarate + AH_2_ **EC:1.1.99.2** | None | Mitochondria (5) | None |
| 8. D-2-hydroxyglutarate dehydrogenase (D2HGDH; Q8N465) | Catalyzes the oxidation of D-2-hydroxyglutarate to alpha-ketoglutarate./ (R)-2-hydroxyglutarate + A → 2-oxoglutarate + AH_2_ **EC:1.1.99.-** | DHRS4L2 (2); ZNF462 (2) | Mitochondria (5) | None |
| 9. Aldehyde oxidase (AOX1; Q06278) | Oxidase with broad substrate specificity, oxidizing aromatic azaheterocycles, such as N^1^-methylnicotinamide, N-methylphthalazinium and phthalazine, as well as aldehydes, such as benzaldehyde, retinal, pyridoxal, and vanillin. Plays a key role in the metabolism of xenobiotics and drugs containing aromatic azaheterocyclic substituents. Participates in the bioactivation of prodrugs such as famciclovir, catalyzing the oxidation step from 6-deoxypenciclovir to penciclovir, which is a potent antiviral agent. Is probably involved in the regulation of reactive oxygen species homeostasis. May be a prominent source of superoxide generation via the one-electron reduction of molecular oxygen. Also may catalyze nitric oxide (NO) production via the reduction of nitrite to NO with NADH or aldehyde as electron donor. May play a role in adipogenesis./  Aldehyde + H_2_O + O_2_ → Carboxylate + H^+^ + H_2_O_2_  **EC:1.2.3.1**;  H_2_O + O_2_ + retinal → H^+^ + H_2_O_2_ + retinoate | None | Cytosol (5), extracelular (4) | None |
| 10. Dihydropyrimidine dehydrogenase (DPYD; Q12882) | 5,6-dihydrouracil + NADP^+^ → H^+^ + NADPH + uracil  **EC:1.3.1.2** | GOPC (2) | Cytosol (5), nucleus (3) | None |
| 11. Delta(24)-sterol reductase (DHCR24; Q15392) | Catalyzes the reduction of the delta-24 double bond of sterol intermediates during cholesterol biosynthesis. In addition to its cholesterol-synthesizing activity, can protect cells from oxidative stress by reducing caspase 3 activity during apoptosis induced by oxidative stress. Also protects against amyloid-beta peptide-induced apoptosis. / Cholesterol + NADP^+^ → cholesta-5,24-dien-3β-ol + H^+^ + NADPH **EC:1.3.1.723**;  H^+^ + lanosterol + NADPH → 24,25-dihydrolanosterol + NADP^+^; 5α-cholest-8-en-3β-ol + NADP^+^ = 5α-cholesta-8,24-dien-3β-ol + H^+^ + NADPH;  **EC:1.3.1.72** | ACTA2 (2), APP (2), ATP12A (2), ERAP1 (2), FUNDC2 (2), HRAS (2), LUZP1 (2) | Nucleus (5),  endoplasmic reticulum (5),  Golgi apparatus (4),  cytoskeleton (3),  cytosol (3). | None |
| 12. Dihydroorotate dehydrogenase (DHODH, Q02127) | (*S*)-dihydroorotate + a quinone → a quinol + orotate **EC:1.3.5.2** | None | Mitochondrion (5), nucleus (5), cytosol (5). | None |
| 13. Peroxisomal acyl-coenzyme A oxidase 1 (ACOX1, Q15067) | Catalyzes the desaturation of acyl-CoAs to 2-trans-enoyl-CoAs. First enzyme of the fatty acid beta-oxidation pathway./ 2,3-saturated acyl-CoA + O_2_ → (2*E*)-enoyl-CoA + H_2_O_2_ **EC:1.3.3.6**; hexadecanoyl-CoA + O_2_ → (2*E*)-hexadecenoyl-CoA + H_2_O_2_; dodecanoyl-CoA + O_2_ → (2*E*)-dodecenoyl-CoA + H_2_O_2_; O_2_ + octanoyl-CoA → (2*E*)-octenoyl-CoA + H_2_O_2_; decanoyl-CoA + O_2_ = (2*E*)-decenoyl-CoA + H_2_O_2_ ; O_2_ + tetradecanoyl-CoA → (2*E*)-tetradecenoyl-CoA + H_2_O_2_; hexadecanedioyl-CoA + O_2_ → (2*E*)-hexadecenedioyl-CoA + H_2_O_2_; O_2_ + tetracosanoyl-CoA → (2*E*)-tetracosenoyl-CoA + H_2_O_2_; glutaryl-CoA + O_2_ → (2*E*)-glutaconyl-CoA + H_2_O_2_; hexanoyl-CoA + O_2_ → (2*E*)-hexenoyl-CoA + H_2_O_2_; O_2_ + octadecanoyl-CoA → (2*E*)-octadecenoyl-CoA + H_2_O_2_. | PEX5 (4), CAT (3), FOXS1 (2), MBNL2 (2), UBE2D2 (2). | Peroxisome (5), cytosol (4), plasma membrane (3), mitochondrion (3),  nucleus (3). | None |
| 14. Peroxisomal acyl-coenzyme A oxidase 2 (ACOX2; Q99424) | Oxidizes the CoA esters of the bile acid intermediates di- and tri-hydroxycholestanoic acids. Capable of oxidizing short as well as long chain 2-methyl branched fatty acids./(25*R*)-3α,7α,12α-trihydroxy-5β-cholestan-26-oyl-CoA + A + H2O → (24*R*,25*R*)-3α,7α,12α,24-tetrahydroxy-5β-cholestan-26-oyl-CoA + AH_2_  **EC:1.17.99.31**;  (25S)-3α,7α,12α-trihydroxy-5β-cholestan-26-oyl-CoA + O_2_ → (24*E*)-3α,7α,12α-trihydroxy-5β-cholest-24-en-26-oyl-CoA + H_2_O_2_ | None | Peroxisome (5), cytosol (5) | None |
| 15. Peroxisomal acyl-coenzyme A oxidase 3  (ACOX3; O15254) | Oxidizes the CoA-esters of 2-methyl-branched fatty acids./ 2,3-saturated acyl-CoA + O_2_ → a (2*E*)-enoyl-CoA + H_2_O_2_ **EC:1.3.3.6**; (2*S*)-pristanoyl-CoA + O_2_ → (2*E*)-pristenoyl-CoA + H_2_O_2_; O_2_ + tetracosanoyl-CoA → (2*E*)-tetracosenoyl-CoA + H_2_O_2_; hexadecanoyl-CoA + O_2_ → (2*E*)-hexadecenoyl-CoA + H_2_O_2_; hexadecanedioyl-CoA + O_2_ → (2*E*)-hexadecenedioyl-CoA + H_2_O_2_ | FKBP6 (2), MICU2 (2), TMEM150A (2) | Peroxisome (5), cytosol (4) | None |
| 16. Succinate-hydroxymethylglutarate CoA-transferase  (SUGCT; Q9HAC7) | Catalyzes the succinyl-CoA-dependent conversion of glutarate to glutaryl-CoA. Can use different dicarboxylic acids as CoA acceptors, the preferred ones are glutarate, succinate, adipate, and 3-hydroxymethylglutarate./ 3-hydroxy-3-methylglutarate + succinyl-CoA → (3*S*)-hydroxy-3-methylglutaryl-CoA + succinate  **EC:2.8.3.13** | SKAP1 (2) | Mitochondrion (5) | None |
| 17. Succinate dehydrogenase [ubiquinone] flavoprotein subunit, mitochondrial  (SDHA; P31040) | Flavoprotein (FP) subunit of succinate dehydrogenase (SDH) that is involved in complex II of the mitochondrial electron transport chain and is responsible for transferring electrons from succinate to ubiquinone (coenzyme Q). Can act as a tumor suppressor. / quinone + succinate → quinol + fumarate  **EC:1.3.5.11** | SDHB (3), ACAD9 (2), ATP5F1 (2), ATXN3 (2), CS (2), CYB5R3 (2), EMC2 (2), MDH2 (2), NDUFV1 (2), PDHA1 (2), PTPN3 (2), SDHAF2 (2), SLC27A2 (2), SUCLG2 (2), UQCRB (2), UQCRFS1 (2) | Mitochondrion (5), nucleus (4) | None |
| 18. Short-chain specific acyl-CoA dehydrogenase, mitochondrial  (ACADS; P16219). | Short-chain specific acyl-CoA dehydrogenase is one of the acyl-CoA dehydrogenases that catalyze the first step of mitochondrial fatty acid beta-oxidation, an aerobic process breaking down fatty acids into acetyl-CoA and allowing the production of energy from fats. The first step of fatty acid beta-oxidation consists in the removal of one hydrogen from C-2 and C-3 of the straight-chain fatty acyl-CoA thioester, resulting in the formation of trans-2-enoyl-CoA. Among the different mitochondrial acyl-CoA dehydrogenases, short-chain specific acyl-CoA dehydrogenase acts specifically on acyl-CoAs with saturated 4 to 6 carbons long primary chains. /Short-chain 2,3-saturated fatty acyl-CoA + H^+^ + oxidized [electron-transfer flavoprotein] → short-chain (2*E*)-enoyl-CoA + reduced [electron-transfer flavoprotein]  **EC:1.3.8.11**;  butanoyl-CoA + H^+^ + oxidized [electron-transfer flavoprotein] → (2*E*)-butenoyl-CoA + reduced [electron-transfer flavoprotein]  **EC:1.3.8.11;**  H^+^ + oxidized [electron-transfer flavoprotein] + pentanoyl-CoA → (2*E*)-pentenoyl-CoA + reduced [electron-transfer flavoprotein];  H^+^ + hexanoyl-CoA + oxidized [electron-transfer flavoprotein] → (2*E*)-hexenoyl-CoA + reduced [electron-transfer flavoprotein] | SEC22C (2) | Cytoskeleton (5), mitochondrion (5), nucleus (5), cytosol (3) | None |
| 19. Medium-chain specific acyl-CoA dehydrogenase, mitochondrial  (ACADM; P11310) | Medium-chain specific acyl-CoA dehydrogenase is one of the acyl-CoA dehydrogenases that catalyze the first step of mitochondrial fatty acid beta-oxidation, an aerobic process breaking down fatty acids into acetyl-CoA and allowing the production of energy from fats. The first step of fatty acid beta-oxidation consists in the removal of one hydrogen from C-2 and C-3 of the straight-chain fatty acyl-CoA thioester, resulting in the formation of trans-2-enoyl-CoA. Electron transfer flavoprotein (ETF) is the electron acceptor that transfers electrons to the main mitochondrial respiratory chain via ETF-ubiquinone oxidoreductase (ETF dehydrogenase). Among the different mitochondrial acyl-CoA dehydrogenases, medium-chain specific acyl-CoA dehydrogenase acts specifically on acyl-CoAs with saturated 6 to 12 carbons long primary chains./ a medium-chain 2,3-saturated fatty acyl-CoA + H^+^ + oxidized [electron-transfer flavoprotein] → a medium-chain trans-(2*E*)-enoyl-CoA + reduced [electron-transfer flavoprotein] **EC:1.3.8.7**;  H^+^ + oxidized [electron-transfer flavoprotein] + pentanoyl-CoA → (2*E*)-pentenoyl-CoA + reduced [electron-transfer flavoprotein]; H^+^ + hexanoyl-CoA + oxidized [electron-transfer flavoprotein] → (2*E*)-hexenoyl-CoA + reduced [electron-transfer flavoprotein]; H^+^ + octanoyl-CoA + oxidized [electron-transfer flavoprotein] → (2*E*)-octenoyl-CoA + reduced [electron-transfer flavoprotein];  decanoyl-CoA + H^+^ + oxidized [electron-transfer flavoprotein] → (2*E*)-decenoyl-CoA + reduced [electron-transfer flavoprotein]; dodecanoyl-CoA + H^+^ + oxidized [electron-transfer flavoprotein] → (2*E*)-dodecenoyl-CoA + reduced [electron-transfer flavoprotein];  H+ + oxidized [electron-transfer flavoprotein] + tetradecanoyl-CoA → (2*E*)-tetradecenoyl-CoA + reduced [electron-transfer flavoprotein];  H^+^ + hexadecanoyl-CoA + oxidized [electron-transfer flavoprotein] → (2*E*)-hexadecenoyl-CoA + reduced [electron-transfer flavoprotein] | None | Mitochondrion (5), nucleus (5), peroxisome (3), cytosol (3) | None |
| 20. Glutaryl-CoA dehydrogenase, mitochondrial  (GCDH; Q92947) | Catalyzes the oxidative decarboxylation of glutaryl-CoA to crotonyl-CoA and CO2 in the degradative pathway of L-lysine, L-hydroxylysine, and L-tryptophan metabolism. It uses electron transfer flavoprotein as its electron acceptor./ Glutaryl-CoA + 2 H^+^ + oxidized [electron-transfer flavoprotein] → (2*E*)-butenoyl-CoA + CO_2_ + reduced [electron-transfer flavoprotein] **EC:1.3.8.6** | ISCA1 (2), MARS2 (2), NOS3 (2) | Mitochondrion (5) | None |
| 21. Isovaleryl-CoA dehydrogenase, mitochondrial  (IVD; P26440) | Catalyzes the conversion of isovaleryl-CoA/3-methylbutanoyl-CoA to 3-methylbut-2-enoyl-CoA as an intermediate step in the leucine (Leu) catabolic pathway. To a lesser extent, is also able to catalyze the oxidation of other saturated short-chain acyl-CoA thioesters as pentanoyl-CoA, hexenoyl-CoA and butenoyl-CoA./ 3-methylbutanoyl-CoA + H^+^ + oxidized [electron-transfer flavoprotein] → 3-methylbut-2-enoyl-CoA + reduced [electron-transfer flavoprotein] **EC:1.3.8.4**; H^+^ + oxidized [electron-transfer flavoprotein] + pentanoyl-CoA → (2*E*)-pentenoyl-CoA + reduced [electron-transfer flavoprotein]; H^+^ + hexanoyl-CoA + oxidized [electron-transfer flavoprotein] → (2*E*)-hexenoyl-CoA + reduced [electron-transfer flavoprotein]; butanoyl-CoA + H^+^ + oxidized [electron-transfer flavoprotein] → (2*E*)-butenoyl-CoA + reduced [electron-transfer flavoprotein] **EC:1.3.8.1** | ETFA (2), PMPCA (2) | Mitochondrion (5), nucleus (3), cytosol (3) | None |
| 22. Short/branched chain specific acyl-CoA dehydrogenase, mitochondrial  (ACADSB; P45954). | Short and branched chain specific acyl-CoA dehydrogenase that catalyzes the removal of one hydrogen from C-2 and C-3 of the fatty acyl-CoA thioester, resulting in the formation of trans-2-enoyl-CoA. Among the different mitochondrial acyl-CoA dehydrogenases, acts specifically on short and branched chain acyl-CoA derivatives such as (*S*)-2-methylbutyryl-CoA as well as short straight chain acyl-CoAs such as butyryl-CoA. Plays an important role in the metabolism of L-isoleucine by catalyzing the dehydrogenation of 2-methylbutyryl-CoA, one of the steps of the L-isoleucine catabolic pathway. Can also act on valproyl-CoA, a metabolite of valproic acid, an antiepileptic drug./  2-methylbutanoyl-CoA + H^+^ + oxidized [electron-transfer flavoprotein] → (2*E*)-2-methylbut-2-enoyl-CoA + reduced [electron-transfer flavoprotein];  (2*S*)-2-methylbutanoyl-CoA + H^+^ + oxidized [electron-transfer flavoprotein] → (2*E*)-2-methylbut-2-enoyl-CoA + reduced [electron-transfer flavoprotein]; (2*R*)-2-methylbutanoyl-CoA + H^+^ + oxidized [electron-transfer flavoprotein] → ethylacryloyl-CoA + reduced [electron-transfer flavoprotein]; butanoyl-CoA + H^+^ + oxidized [electron-transfer flavoprotein] → (2*E*)-butenoyl-CoA + reduced [electron-transfer flavoprotein]; 2-methylpropanoyl-CoA + H^+^ + oxidized [electron-transfer flavoprotein] = (2*E*)-2-methylpropenoyl-CoA + reduced [electron-transfer flavoprotein]; H^+^ + hexanoyl-CoA + oxidized [electron-transfer flavoprotein] → (2*E*)-hexenoyl-CoA + reduced [electron-transfer flavoprotein]; 2-methylhexanoyl-CoA + H^+^ + oxidized [electron-transfer flavoprotein] → 2-methylhexenoyl-CoA + reduced [electron-transfer flavoprotein];  H^+^ + oxidized [electron-transfer flavoprotein] + valproyl-CoA → (2*E*)-2-propylpent-2-enoyl-CoA + reduced [electron-transfer flavoprotein] | None | Mitochondrion (5) | None |
| 23. Long-chain specific acyl-CoA dehydrogenase, mitochondrial  (ACADL; P28330) | a long-chain 2,3-saturated fatty acyl-CoA + H^+^ + oxidized [electron-transfer flavoprotein] → a long-chain (2*E*)-enoyl-CoA + reduced [electron-transfer flavoprotein] **EC:1.3.8.8**; H^+^ + hexanoyl-CoA + oxidized [electron-transfer flavoprotein] → (2*E*)-hexenoyl-CoA + reduced [electron-transfer flavoprotein]; H+ + octanoyl-CoA + oxidized [electron-transfer flavoprotein] → (2*E*)-octenoyl-CoA + reduced [electron-transfer flavoprotein]; decanoyl-CoA + H^+^ + oxidized [electron-transfer flavoprotein] → (2*E*)-decenoyl-CoA + reduced [electron-transfer flavoprotein]; dodecanoyl-CoA + H^+^ + oxidized [electron-transfer flavoprotein] → (2*E*)-dodecenoyl-CoA + reduced [electron-transfer flavoprotein]; H^+^ + oxidized [electron-transfer flavoprotein] + tetradecanoyl-CoA → (2*E*)-tetradecenoyl-CoA + reduced [electron-transfer flavoprotein]; H^+^ + hexadecanoyl-CoA + oxidized [electron-transfer flavoprotein] → (2*E*)-hexadecenoyl-CoA + reduced [electron-transfer flavoprotein]; H^+^ + octadecanoyl-CoA + oxidized [electron-transfer flavoprotein] → (2*E*)-octadecenoyl-CoA + reduced [electron-transfer flavoprotein]; eicosanoyl-CoA + H^+^ + oxidized [electron-transfer flavoprotein] → (2*E*)-eicosenoyl-CoA + reduced [electron-transfer flavoprotein]; docosanoyl-CoA + H^+^ + oxidized [electron-transfer flavoprotein] → (2*E*)-docosenoyl-CoA + reduced [electron-transfer flavoprotein];  H^+^ + oxidized [electron-transfer flavoprotein] + tetracosanoyl-CoA → (2*E*)-tetracosenoyl-CoA + reduced [electron-transfer flavoprotein]; (5*E*)-tetradecenoyl-CoA + H+ + oxidized [electron-transfer flavoprotein] → (2*E*,5*E*)-tetradecadienoyl-CoA + reduced [electron-transfer flavoprotein]; (5*Z*)-tetradecenoyl-CoA + H^+^ + oxidized [electron-transfer flavoprotein] → (2*E*,5*Z*)-tetradecadienoyl-CoA + reduced [electron-transfer flavoprotein]; (9*Z*)-octadecenoyl-CoA + H^+^ + oxidized [electron-transfer flavoprotein] → (2E,9Z)-octadecadienoyl-CoA + reduced [electron-transfer flavoprotein] | SYT2 (2), TMPRSS11A (2) | Mitochondrion (5), cytosol (3) | None |
| 24. Very long-chain specific acyl-CoA dehydrogenase, mitochondrial  (ACADVL; P49748). | Very long-chain specific acyl-CoA dehydrogenase is one of the acyl-CoA dehydrogenases that catalyze the first step of mitochondrial fatty acid beta-oxidation, an aerobic process breaking down fatty acids into acetyl-CoA and allowing the production of energy from fats. The first step of fatty acid beta-oxidation consists in the removal of one hydrogen from C-2 and C-3 of the straight-chain fatty acyl-CoA thioester, resulting in the formation of trans-2-enoyl-CoA. Among the different mitochondrial acyl-CoA dehydrogenases, very long-chain specific acyl-CoA dehydrogenase acts specifically on acyl-CoAs with saturated 12 to 24 carbons long primary chains./  a very-long-chain 2,3-saturated fatty acyl-CoA + H^+^ + oxidized [electron-transfer flavoprotein] → a very-long-chain (2*E*)-enoyl-CoA + reduced [electron-transfer flavoprotein] **EC:1.3.8.9**; decanoyl-CoA + H^+^ + oxidized [electron-transfer flavoprotein] = (2*E*)-decenoyl-CoA + reduced [electron-transfer flavoprotein];  dodecanoyl-CoA + H^+^ + oxidized [electron-transfer flavoprotein] → (2*E*)-dodecenoyl-CoA + reduced [electron-transfer flavoprotein]; H^+^ + oxidized [electron-transfer flavoprotein] + tetradecanoyl-CoA → (2*E*)-tetradecenoyl-CoA + reduced [electron-transfer flavoprotein]; H^+^ + hexadecanoyl-CoA + oxidized [electron-transfer flavoprotein] → (2*E*)-hexadecenoyl-CoA + reduced [electron-transfer flavoprotein]; H^+^ + octadecanoyl-CoA + oxidized [electron-transfer flavoprotein] → (2*E*)-octadecenoyl-CoA + reduced [electron-transfer flavoprotein]; eicosanoyl-CoA + H^+^ + oxidized [electron-transfer flavoprotein] → (2*E*)-eicosenoyl-CoA + reduced [electron-transfer flavoprotein];  docosanoyl-CoA + H^+^ + oxidized [electron-transfer flavoprotein] → (2*E*)-docosenoyl-CoA + reduced [electron-transfer flavoprotein]; H^+^ + oxidized [electron-transfer flavoprotein] + tetracosanoyl-CoA → (2*E*)-tetracosenoyl-CoA + reduced [electron-transfer flavoprotein]; (9*Z*)-hexadecenoyl-CoA + H^+^ + oxidized [electron-transfer flavoprotein] → (2*E*,9*Z*)-hexadecadienoyl-CoA + reduced [electron-transfer flavoprotein];  (9*Z*)-octadecenoyl-CoA + H^+^ + oxidized [electron-transfer flavoprotein] → (2*E*,9*Z*)-octadecadienoyl-CoA + reduced [electron-transfer flavoprotein] | SURF1 (3), ECSIT (2), HIBCH (2), HMGCL (2), MRM1 (2), SIRT3 (2), SOCS3 (2) | Mitochondrion (5), nucleus (5), cytosol (5), peroxisome (3) | None |
| 25. Isobutyryl-CoA dehydrogenase, mitochondrial (ACAD8; Q9UKU7) | Isobutyryl-CoA dehydrogenase which catalyzes one of the steps of the valine catabolic pathway. To a lesser extent, is also able to catalyze the oxidation of (2*S*)-2-methylbutanoyl-CoA./2-methylpropanoyl-CoA + H^+^ + oxidized [electron-transfer flavoprotein] → (2*E*)-2-methylpropenoyl-CoA + reduced [electron-transfer flavoprotein]; (2*S*)-2-methylbutanoyl-CoA + H^+^ + oxidized [electron-transfer flavoprotein] → (2*E*)-2-methylbutenoyl-CoA + reduced [electron-transfer flavoprotein]; H^+^ + oxidized [electron-transfer flavoprotein] + propanoyl-CoA → acryloyl-CoA + reduced [electron-transfer flavoprotein] | SRGAP3 (2), VSTM2A (2) | Mitochondrion (5) | None |
| 26. Complex I assembly factor ACAD9, mitochondrial  (ACAD9; Q9H845) | As part of the MCIA complex, primarily participates in the assembly of the mitochondrial complex I and therefore plays a role in oxidative phosphorylation. This moonlighting protein has also a dehydrogenase activity toward a broad range of substrates with greater specificity for long-chain unsaturated acyl-CoAs. However, in vivo, it does not seem to play a primary role in fatty acid oxidation. In addition, the function in complex I assembly is independent of the dehydrogenase activity of the protein. /eicosanoyl-CoA + H^+^ + oxidized [electron-transfer flavoprotein] → (2*E*)-eicosenoyl-CoA + reduced [electron-transfer flavoprotein]; H^+^ + octadecanoyl-CoA + oxidized [electron-transfer flavoprotein] → (2*E*)-octadecenoyl-CoA + reduced [electron-transfer flavoprotein]; H^+^ + hexadecanoyl-CoA + oxidized [electron-transfer flavoprotein] → (2*E*)-hexadecenoyl-CoA + reduced [electron-transfer flavoprotein]; decanoyl-CoA + H+ + oxidized [electron-transfer flavoprotein] → (2*E*)-decenoyl-CoA + reduced [electron-transfer flavoprotein];  H+ + nonanoyl-CoA + oxidized [electron-transfer flavoprotein] → (2*E*)-nonenoyl-CoA + reduced [electron-transfer flavoprotein]; H^+^ + oxidized [electron-transfer flavoprotein] + pentadecanoyl-CoA → (2*E*)-pentadecenoyl-CoA + reduced [electron-transfer flavoprotein]; H+ + oxidized [electron-transfer flavoprotein] + undecanoyl-CoA → reduced [electron-transfer flavoprotein] + trans-2-undecenoyl-CoA;  (9*Z*)-hexadecenoyl-CoA + H^+^ + oxidized [electron-transfer flavoprotein] → (2*E*,9*Z*)-hexadecadienoyl-CoA + reduced [electron-transfer flavoprotein]; H^+^ + heptadecanoyl-CoA + oxidized [electron-transfer flavoprotein] → reduced [electron-transfer flavoprotein] + trans-2-heptadecenoyl-CoA; (9*E*)-octadecenoyl-CoA + H^+^ + oxidized [electron-transfer flavoprotein] → (2*E*,9*E*)-octadecadienoyl-CoA + reduced [electron-transfer flavoprotein]; (9*Z*)-octadecenoyl-CoA + H^+^ + oxidized [electron-transfer flavoprotein] → (2*E*,9*Z*)-octadecadienoyl-CoA + reduced [electron-transfer flavoprotein]; (9*Z*,12*Z*)-octadecadienoyl-CoA + H^+^ + oxidized [electron-transfer flavoprotein] → (2*E*,9*Z*,12*Z*)-octadecatrienoyl-CoA + reduced [electron-transfer flavoprotein];  (4*Z*,7*Z*,10*Z*,13*Z*,16*Z*,19*Z*)-docosahexaenoyl-CoA + H^+^ + oxidized [electron-transfer flavoprotein] → (2*E*,4*Z*,7*Z*,10*Z*,13*Z*,16*Z*,19*Z*)-docosaheptaenoyl-CoA + reduced [electron-transfer flavoprotein];  H^+^ + oxidized [electron-transfer flavoprotein] + tetradecanoyl-CoA → (2*E*)-tetradecenoyl-CoA + reduced [electron-transfer flavoprotein] | ECSIT (5), NDUFS3 (5), FAF2 (4), NDUFAF1 (4), TIMMDC1 (4), DBT (3), EYA2 (3), NDUFA13 (3), NDUFAF4 (3), NDUFS7 (3), PDK1 (3), COA1 (2), DLAT (2), ERAL1 (2), GLS (2), GRSF1 (2), HSPD1 (2), ICT1 (2), MRPL1 (2), MTRF1L (2), NDUFA2 (2), NDUFA8 (2), NDUFAF3 (2), NDUFS2 (2), NDUFS5 (2), NFS1 (2), P2RY12 (2), PDK2 (2), PDK3 (2), PDSS1 (2), PNPT1 (2), SDHA (2), SIRT3 (2), TRMT61B (2) | mitochondrion (5), nucleus (5) | None |
| 27. Acyl-CoA dehydrogenase family member 10  (ACAD10; Q6JQN1) | Acyl-CoA dehydrogenase only active with *R*- and *S*-2-methyl-C15-CoA./ A + a 2,3-saturated acyl-CoA → a 2,3-dehydroacyl-CoA + AH_2_ | BSG (2), NDUFA10 (2), P2RY8 (2), PTH1R (2) | Mitochondrion (5) | None. |
| 28. Acyl-CoA dehydrogenase family member 11  (ACAD11; Q709F0) | Acyl-CoA dehydrogenase, that exhibits maximal activity towards saturated C22-CoA. Probably participates in beta-oxydation and energy production but could also play a role in the metabolism of specific fatty acids to control fatty acids composition of cellular lipids in brain./ a 2,3-saturated acyl-CoA + H^+^ + oxidized [electron-transfer flavoprotein] → a (2*E*)-enoyl-CoA + reduced [electron-transfer flavoprotein]; docosanoyl-CoA + H^+^ + oxidized [electron-transfer flavoprotein] → (2*E*)-docosenoyl-CoA + reduced [electron-transfer flavoprotein]; H^+^ + oxidized [electron-transfer flavoprotein] + tetracosanoyl-CoA → (2*E*)-tetracosenoyl-CoA + reduced [electron-transfer flavoprotein]; eicosanoyl-CoA + H^+^ + oxidized [electron-transfer flavoprotein] → (2*E*)-eicosenoyl-CoA + reduced [electron-transfer flavoprotein]; H^+^ + hexacosanoyl-CoA + oxidized [electron-transfer flavoprotein] → (2*E*)-hexacosenoyl-CoA + reduced [electron-transfer flavoprotein]; H+ + oxidized [electron-transfer flavoprotein] + tricosanoyl-CoA → (2*E*)-tricosenoyl-CoA + reduced [electron-transfer flavoprotein] | CD2BP2 (3), DUSP19 (2), FAM9A (2), GTSE1 (2), KCTD17 (2), MIER2 (2), MTMR4 (2), PDYN (2), PEX5 (2), PTPN14 (2), SDF4 (2), TCL1B (2), TRAK2 (2), TTC4 (2), UBXN7 (2) | Mitochondrion (5), nucleus (5),  peroxisome (4) | None. |
| 29. D-aspartate oxidase  (DDO; Q99489) | Selectively catalyzes the oxidative deamination of D-aspartate and its N-methylated derivative, N-methyl D-aspartate./ D-aspartate + H_2_O + O_2_ → H_2_O_2_ + NH_4_^+^ + oxaloacetate **EC:1.4.3.1**. | PEX5 (2) | Peroxisome (5), cytosol (5) | None |
| 30. L-amino-acid oxidase  (IL4I1; Q96RQ9). | Secreted L-amino-acid oxidase that acts as a key immunoregulator. Has preference for L-aromatic amino acids: converts phenylalanine (Phe), tyrosine (Tyr) and tryptophan (Trp) to phenylpyruvic acid (PP), hydroxyphenylpyruvic acid (HPP), and indole-3-pyruvic acid (I3P), respectively. Also has weak L-arginine oxidase activity. Acts as a negative regulator of anti-tumor immunity by mediating Trp degradation via an indole pyruvate pathway that activates the transcription factor AHR. IL4I1-mediated Trp catabolism generates I3P, giving rise to indole metabolites (indole-3-acetic acid (IAA) and indole-3-aldehyde (I3A)) and kynurenic acid, which act as ligands for AHR, a ligand-activated transcription factor that plays important roles in immunity and cancer. AHR activation by indoles following IL4I1-mediated Trp degradation enhances tumor progression by promoting cancer cell motility and suppressing adaptive immunity. Also has an immunoregulatory function in some immune cells, probably by mediating Trp degradation and promoting downstream AHR activation: inhibits T-cell activation and proliferation, promotes the differentiation of naive CD4+ T-cells into FOXP3+ regulatory T-cells (Treg) and regulates the development and function of B-cells. Also regulates M2 macrophage polarization by inhibiting T-cell activation. Also has antibacterial properties by inhibiting growth of Gram negative and Gram positive bacteria through the production of NH_4_^+^ and H_2_O_2_./ an L-α-amino acid + H_2_O + O_2_ → a 2-oxocarboxylate + H_2_O_2_ + NH_4_^+^ **EC:1.4.3.2;** H_2_O + L-tryptophan + O_2_ → H_2_O_2_ + indole-3-pyruvate + NH_4_^+^; H_2_O + L-phenylalanine + O_2_ → 3-phenylpyruvate + H_2_O_2_ + NH_4_^+^;  H_2_O + L-tyrosine + O_2_ → 3-(4-hydroxyphenyl)pyruvate + H_2_O_2_ + NH_4_^+^ ;  H_2_O + L-arginine + O_2_ → 5-guanidino-2-oxopentanoate + H_2_O_2_ + NH_4_^+^ | None | Extracellular (4), lysosome (4),  plasma membrane (3) | None |
| 31. D-amino-acid oxidase  (DAO; P14920) | Regulates the level of the neuromodulator D-serine in the brain. Has high activity towards D-DOPA and contributes to dopamine synthesis. Could act as a detoxifying agent which removes D-amino acids accumulated during aging. Acts on a variety of D-amino acids with a preference for those having small hydrophobic side chains followed by those bearing polar, aromatic, and basic groups. Does not act on acidic amino acids./ a D-α-amino acid + H_2_O + O_2_ → a 2-oxocarboxylate + H_2_O_2_ + NH_4_^+^ **EC:1.4.3.3** | DAOA (3), PRKAB2 (3) | Peroxisome (5), cytosol (5),  extracellular (3), nucleus (3) | None |
| 32. Amine oxidase [flavin-containing] A  (MAOA; P21397) | Catalyzes the oxidative deamination of biogenic and xenobiotic amines and has important functions in the metabolism of neuroactive and vasoactive amines in the central nervous system and peripheral tissues. MAOA preferentially oxidizes biogenic amines such as 5-hydroxytryptamine (5-HT), norepinephrine and epinephrine./  a secondary aliphatic amine + H_2_O + O_2_ → a primary amine + an aldehyde + H_2_O_2_ **EC:1.4.3.4** | MAOB (2) | mitochondrion (5), cytosol (5),  nucleus (3) | None |
| 33. Amine oxidase [flavin-containing] B (MAOB; P27338) | Catalyzes the oxidative deamination of biogenic and xenobiotic amines and has important functions in the metabolism of neuroactive and vasoactive amines in the central nervous system and peripheral tissues. MAOB preferentially degrades benzylamine and phenylethylamine./  a secondary aliphatic amine + H_2_O + O_2_ → a primary amine + an aldehyde + H_2_O_2_  **EC:1.4.3.4** | MAOA (2) | mitochondrion (5), nucleus (3),  cytosol (3) | None |
| 34. Pyridoxine-5'-phosphate oxidase  (PNPO; Q9NVS9) | Catalyzes the oxidation of either pyridoxine 5'-phosphate (PNP) or pyridoxamine 5'-phosphate (PMP) into pyridoxal 5'-phosphate (PLP)./ H_2_O + O_2_ + pyridoxamine 5'-phosphate → H_2_O_2_ + NH_4_^+^ + pyridoxal 5'-phosphate  **EC:1.4.3.5**;  O_2_ + pyridoxine 5'-phosphate → H_2_O_2_ + pyridoxal 5'-phosphate  **EC:1.4.3.5** | AGTRAP (2), ARL6IP6 (2), FBXO25 (2), MOK (2), MTERF1 (2) | nucleus (5), cytosol (5),  mitochondrion (3) | None |
| 35. Renalase  (RNLS; Q5VYX0) | Catalyzes the oxidation of the less abundant 1,2-dihydro-beta-NAD(P) and 1,6-dihydro-beta-NAD(P) to form beta-NAD(P)+. The enzyme hormone is secreted by the kidney, and circulates in blood and modulates cardiac function and systemic blood pressure. Lowers blood pressure in vivo by decreasing cardiac contractility and heart rate and preventing a compensatory increase in peripheral vascular tone, suggesting a causal link to the increased plasma catecholamine and heightened cardiovascular risk. High concentrations of catecholamines activate plasma renalase and promotes its secretion and synthesis./1,2-dihydro-β-NAD + H^+^ + O_2_ → H_2_O_2_ + NAD^+^  **EC:1.6.3.5**; 1,2-dihydro-β-NADP + H^+^ + O_2_ → H_2_O_2_ + NADP^+^ **EC:1.6.3.5**; 1,6-dihydro-β-NADP + H^+^ + O_2_ → H_2_O_2_ + NADP^+^ **EC:1.6.3.5**; 1,6-dihydro-β-NAD + H^+^ + O_2_ → H_2_O_2_ + NAD^+^ **EC:1.6.3.5** | None | Extracellular (5) | None |
| 36. Methylenetetrahydrofolate reductase (MTHFR; P42898) | Catalyzes the conversion of 5,10-methylenetetrahydrofolate to 5-methyltetrahydrofolate, a co-substrate for homocysteine remethylation to methionine. /(6*S*)-5-methyl-5,6,7,8-tetrahydrofolate + NADP^+^ → (6*R*)-5,10-methylene-5,6,7,8-tetrahydrofolate + H^+^ + NADPH **EC:1.5.1.20**; (6*S*)-5-methyl-5,6,7,8-tetrahydrofolate + NAD^+^ → (6*R*)-5,10-methylene-5,6,7,8-tetrahydrofolate + H^+^ + NADH **EC:1.5.1.20**. | BCL2L11 (2), CDCA8 (2), GNAI1 (2), GNAZ (2), RASSF1 (2), SMPD2 (2) | Cytosol (4), plasma membrane (3), extracellular (3), nucleus (3) | None |
| 37. Peroxisomal sarcosine oxidase (PIPOX; Q9P0Z9) | Metabolizes sarcosine, L-pipecolic acid and L-proline./ H_2_O + O_2_ + sarcosine → formaldehyde + glycine + H_2_O_2_ **EC:1.5.3.1**; L-pipecolate + O_2_ → H^+^ + H_2_O_2_ + L-1-piperideine-6-carboxylate **EC:1.5.3.7**. | ELP4 (2), ELP5 (2), ELP (6), FKBP5 (2), NAPG (2), ZNF217 (2) | Peroxisome (5), cytosol (4) | None |
| 38. Spermine oxidase (SMOX; Q9NWM0) | Flavoenzyme which catalyzes the oxidation of spermine to spermidine. Can also use N_1_-acetylspermine and spermidine as substrates, with different affinity depending on the isoform (isozyme) and on the experimental conditions. Plays an important role in the regulation of polyamine intracellular concentration and has the potential to act as a determinant of cellular sensitivity to the antitumor polyamine analogs. May contribute to beta-alanine production via aldehyde dehydrogenase conversion of 3-amino-propanal./H_2_O + O_2_ + spermine → 3-aminopropanal + H_2_O_2_ + spermidine **EC:1.5.3.16** | None | Nucleus (5), cytosol (5), plasma membrane (3), extracellular (3) | None |
| 39. Electron transfer flavoprotein-ubiquinone oxidoreductase, mitochondrial  (ETFDH; Q16134) | Accepts electrons from ETF and reduces ubiquinone./ a ubiquinone + reduced [electron-transfer flavoprotein] → a ubiquinol + H^+^ + oxidized [electron-transfer flavoprotein] **EC:1.5.5.1** | None | Mitochondrion (5) | None |
| 40. Electron transfer flavoprotein subunit alpha, mitochondrial  (ETFA; P13804) | Heterodimeric electron transfer flavoprotein that accepts electrons from several mitochondrial dehydrogenases, including acyl-CoA dehydrogenases, glutaryl-CoA and sarcosine dehydrogenase. It transfers the electrons to the main mitochondrial respiratory chain via ETF-ubiquinone oxidoreductase (ETF dehydrogenase). Required for normal mitochondrial fatty acid oxidation and normal amino acid metabolism. | ETFB (5), LYRM5 (5), C8ORF82 (3), AGR2 (2), ATP5A1 (2), COX15 (2), CRYZ (2), GRSF1 (2), HINT2 (2), ICT1 (2), IVD (2) | Mitochondrion (5) | None |
| 41. Electron transfer flavoprotein subunit beta  (ETFB; P38117) | Heterodimeric electron transfer flavoprotein that accepts electrons from several mitochondrial dehydrogenases, including acyl-CoA dehydrogenases, glutaryl-CoA and sarcosine dehydrogenase. It transfers the electrons to the main mitochondrial respiratory chain via ETF-ubiquinone oxidoreductase. Required for normal mitochondrial fatty acid oxidation and normal amino acid metabolism. ETFB binds an AMP molecule that probably has a purely structural role. | ETFA (5), LYRM5 (4),CRYZ (2), GRSF1 (2), LRPPRC (2) | Mitochondrion (5) | None |
| 42. Sarcosine dehydrogenase, mitochondrial  (SARDH, Q9UL12) | H^+^ + H_2_O + oxidized [electron-transfer flavoprotein] + sarcosine → formaldehyde + glycine + reduced [electron-transfer flavoprotein]  **EC:1.5.8.3** | None | Mitochondrion (5) | None |
| 43. Dimethylglycine dehydrogenase, mitochondrial  (DMGDH, Q9UI17) | Catalyzes the demethylation of N,N-dimethylglycine to sarcosine. Also has activity with sarcosine in vitro. / (6*S*)-5,6,7,8-tetrahydrofolyl-(γ-L-Glu)n + H^+^ + N,N-dimethylglycine + oxidized [electron-transfer flavoprotein] → (6*R*)-5,10-methylenetetrahydrofolyl-(γ-L-Glu)(n) + reduced [electron-transfer flavoprotein] + sarcosine **EC:1.5.8.4** | None | Mitochondrion (5) | None |
| 44. Lysine-specific histone demethylase 1A  (KDM1A; O60341) | Histone demethylase that can demethylate both 'Lys-4' (H3K4me) and 'Lys-9' (H3K9me) of histone H3, thereby acting as a coactivator or a corepressor, depending on the context. Acts by oxidizing the substrate by FAD to generate the corresponding imine that is subsequently hydrolyzed. Acts as a corepressor by mediating demethylation of H3K4me, a specific tag for epigenetic transcriptional activation. Demethylates both mono- (H3K4me1) and di-methylated (H3K4me2) H3K4me (. May play a role in the repression of neuronal genes. Alone, it is unable to demethylate H3K4me on nucleosomes and requires the presence of RCOR1/CoREST to achieve such activity. Also acts as a coactivator of androgen receptor (AR)-dependent transcription, by being recruited to AR target genes and mediating demethylation of H3K9me, a specific tag for epigenetic transcriptional repression. The presence of PRKCB in AR-containing complexes, which mediates phosphorylation of 'Thr-6' of histone H3 (H3T6ph), a specific tag that prevents demethylation H3K4me, prevents H3K4me demethylase activity of KDM1A. Demethylates di-methylated 'Lys-370' of p53/TP53 which prevents interaction of p53/TP53 with TP53BP1 and represses p53/TP53-mediated transcriptional activation. Demethylates and stabilizes the DNA methylase DNMT1. Required for gastrulation during embryogenesis. Component of a RCOR/GFI/KDM1A/HDAC complex that suppresses, via histone deacetylase (HDAC) recruitment, a number of genes implicated in multilineage blood cell development. Effector of SNAI1-mediated transcription repression of E-cadherin/CDH1, CDN7 and KRT8. Required for the maintenance of the silenced state of the SNAI1 target genes E-cadherin/CDH1 and CDN7./ 2 A + 2 H_2_O + N^6^,N^6^-dimethyl-L-lysyl^4^-[histone H3] → 2 AH_2_ + 2 formaldehyde + L-lysyl4-[histone H3] **EC:1.14.99.66** | RCOR1 (23), HDAC1 (21), HDAC2 (14), HMG20B (9), SNAI1 (9), CTBP1 (8), HIST1H3A (8), ZNF217 (7), ATG16L1 (6), HMG20A (6), PHF21A (6), TP53 (5), MTA2 (5), USP7 (5), HDAC3 (4), MYC (4), PPP1R12A (4), RCOR3 (4), RIOK1 (4), USP28 (4), ZMYM2 (4), KDM1A (3), BRCA1 (3), CARM1 (3), CSNK2A1 (3), CSNK2A2 (3), DNMT1 (3), ESR1 (3), EZH2 (3), GATA3 (3), GFI1B (3), GSE1 (3), MBD3 (3), MTA1 (3), MTA3 (3), NMI (3), NR2C2 (3), NR2E1 (3), PELP1 (3), PPM1D (3), RNF168 (3), RPRD1A (3), SOCS6 (3), TAL1 (3), TRIM39 (3), USP22 (3), ZMYM3 (3), AKAP9 (2), AR (2), ARRDC1 (2), C15ORF27 (2), C8ORF74 (2), CBFA2T3 (2), CCDC14 (2), CCDC151 (2), CCDC53 (2), CDCA4 (2), CEP70 (2), CHD3 (2), DNAJA3 (2), E2F1 (2), ECE2 (2), FAM9A (2), FYCO1 (2), GOLGA2 (2), GSK3B (2), GSTCD (2), GTF2I (2), GTPBP2 (2), HAUS1 (2), HAUS6 (2), HESX1 (2), HIST2H3C (2), IMMT (2), INSM1 (2), KDM5B (2), KIFC3 (2), KLHDC4 (2), KRT33B (2), NECAB2 (2), NOTCH1 (2), NR1H2 (2), NR1H3 (2), NR2C1 (2), OFCC1 (2), OIP5 (2), PHF19 (2), PPARD (2), PRDM1 (2), PSMC1 (2), RASSF1 (2), RASSF2 (2), RBBP4 (2), RBPJ (2), SAMD3 (2), SERGEF (2), SF3B2 (2), SMAD9 (2), SMARCC1 (2), SNF8 (2), SOX2 (2), SPICE1 (2), SPSB1 (2), STAT3 (2), STX19 (2), TEX9 (2), TNNT2 (2), TRAF4 (2), TRIM28 (2), YEATS4 (2), ZBED1 (2) | Nucleus (5), extracellular (3), cytosol (3) | None. |
| 45. Proline dehydrogenase 1, mitochondrial  (PRODH; O43272) | Converts proline to delta-1-pyrroline-5-carboxylate./ a quinone + L-proline → (*S*)-1-pyrroline-5-carboxylate + a quinol + H^+^  **EC:1.5.5.2** | None | Mitochondrion (5), cytosol (3) | None |
| 46. NADH-cytochrome b5 reductase 3  (CYB5R3; P00387) | 2 [Fe(III)-cytochrome *b*_5_] + NADH → 2 [Fe(II)-cytochrome *b*_5_] + H^+^ + NAD^+^ **EC:1.6.2.2** | RTN4 (3), ACTL6A (2), CPVL (2), PDHA1 (2), SDHA (2). | Extracellular (5), mitochondrion (5),  endoplasmic reticulum (5),  cytosol (4), lysosome (4) | None |
| 47. NADPH-cytochrome P450 reductase  (POR; P16435) | This enzyme is required for electron transfer from NADP to cytochrome P450 in microsomes. It can also provide electron transfer to heme oxygenase and cytochrome B5./ NADPH + 2 oxidized [cytochrome P450] → H^+^ + NADP^+^ + 2 reduced [cytochrome P450] **EC:1.6.2.4** | CYP2E1 (3), FANCC (3), UBXN6 (3), CYP1A2 (2), CYP2C19 (2), CYP2C9 (2). | Endoplasmic reticulum (5), cytosol (4), mitochondrion (3), nucleus (3) | None |
| 48. NAD(P)H dehydrogenase [quinone] 1 (NQO1; P15559) | The enzyme apparently serves as a quinone reductase in connection with conjugation reactions of hydroquinons involved in detoxification pathways as well as in biosynthetic processes such as the vitamin K-dependent gamma-carboxylation of glutamate residues in prothrombin synthesis./ a quinone + H^+^ + NADH → a quinol + NAD^+^ **EC:1.6.5.2;** a quinone + H^+^ + NADPH → a quinol + NADP^+^  **EC:1.6.5.2** | TP53 (5), FOS (3), HIF1A (3), STUB1 (3), BAG3 (2). | Cytosol (5), mitochondrion (3),  nucleus (3) | None |
| 49. NADH dehydrogenase [ubiquinone] flavoprotein 1, mitochondrial  (NDUFV1; P49821) | Core subunit of the mitochondrial membrane respiratory chain NADH dehydrogenase (Complex I) that is believed to belong to the minimal assembly required for catalysis. Complex I functions in the transfer of electrons from NADH to the respiratory chain. The immediate electron acceptor for the enzyme is believed to be ubiquinone. / a ubiquinone + 5 H^+^(in) + NADH = a ubiquinol + 4 H^+^(out) + NAD^+^  **EC:7.1.1.2** | NDUFS3 (6), NDUFA9 (4), NDUFS1 (4), NDUFS2 (4), NDUFA8 (3), NDUFS7 (3), NDUFS8 (3), NDUFV2 (3), C6ORF203 (2), COA3 (2), COX5A (2), CPT1A (2), GRSF1 (2), HSCB (2), ICT1 (2), LONP1 (2), LRPPRC (2), NDUFA2 (2), NDUFS4 (2), NDUFS5 (2), NDUFS6 (2), NDUFV3 (2), PDHA1 (2), PHB2 (2), POR (2), SDHA (2), TOMM40 (2), TUFM (2), VDAC2 (2) | Mitochondrion (5), cytosol (4) | None |
| 50. NADPH-dependent diflavin oxidoreductase 1  (NDOR1; Q9UHB4) | Component of the cytosolic iron-sulfur (Fe-S) protein assembly (CIA) machinery. Required for the maturation of extramitochondrial Fe-S proteins. Part of an electron transfer chain functioning in an early step of cytosolic Fe-S biogenesis. Transfers electrons from NADPH to the Fe/S cluster of CIAPIN1. | CIAPIN1 (4), MTUS2 (2), TCF4 (2), TCHP (2) | Nucleus (5), cytosol (5), cytoskeleton (4) | None |
| 51. tRNA-dihydrouridine(20) synthase [NAD(P)^+^]-like  (DUS2; Q9NX74) | Dihydrouridine synthase. Catalyzes the NADPH-dependent synthesis of dihydrouridine, a modified base found in the D-loop of most tRNAs. Negatively regulates the activation of EIF2AK2/PKR./ 5,6-dihydrouridine^20^ in tRNA + NADP^+^ → H^+^ + NADPH + uridine^20^ in tRNA  **EC:1.3.1.91** | None | Cytosol (5), endoplasmic reticulum (3) | None |
| 52. Dihydrolipoyl dehydrogenase, mitochondrial  (DLD; P09622) | Lipoamide dehydrogenase is a component of the glycine cleavage system as well as an E3 component of three alpha-ketoacid dehydrogenase complexes (pyruvate-, alpha-ketoglutarate-, and branched-chain amino acid-dehydrogenase complex). The 2-oxoglutarate dehydrogenase complex is mainly active in the mitochondrion. A fraction of the 2-oxoglutarate dehydrogenase complex also localizes in the nucleus and is required for lysine succinylation of histones: associates with KAT2A on chromatin and provides succinyl-CoA to histone succinyltransferase KAT2A. In monomeric form may have additional moonlighting function as serine protease. Involved in the hyperactivation of spermatazoa during capacitation and in the spermatazoal acrosome reaction (*R*)-*N*^6^-dihydrolipoyl-L-lysyl-[protein] + NAD^+^ → (*R*)-*N*^6^-lipoyl-L-lysyl-[protein] + H^+^ + NADH **EC:1.8.1.4** | OGDH (4), PDHA1 (4), DBT (3), DLST (3), PDHB (3), PDHX (3), AIFM1 (2), AK2 (2), BTRC (2), C7ORF55 (2), CKB (2), DDX39B (2), DHTKD1 (2), FBXW11 (2), HSD17B10 (2), HSDL2 (2), LDHA (2), MRPS36 (2), NFS1 (2), NUDT21 (2), OAT (2), OGDHL (2), PARS2 (2), PCBP3 (2), PDK3 (2), PSMD4 (2), SIAH1 (2), SLX4IP (2), SOCS6 (2), SORL1 (2), UBE2M (2) | Mitochondrion (5), nucleus (5), cytosol (3) | None |
| 53. Glutathione reductase, mitochondrial  (GSR; P00390) | Maintains high levels of reduced glutathione in the cytosol./ 2 glutathione + NADP^+^ → glutathione disulfide + H^+^ + NADPH **EC:1.8.1.7** | None | Extracellular (5), mitochondrion (5), cytosol (5), plasma membrane (4), nucleus (3) | None |
| 54. Thioredoxin reductase 1, cytoplasmic  (TXNRD1; Q16881) | Isoform 1 may possess glutaredoxin activity as well as thioredoxin reductase activity and induces actin and tubulin polymerization, leading to formation of cell membrane protrusions. Isoform 4 enhances the transcriptional activity of estrogen receptors alpha and beta while isoform 5 enhances the transcriptional activity of the beta receptor only. Isoform 5 also mediates cell death induced by a combination of interferon-beta and retinoic acid./ [thioredoxin]-dithiol + NADP^+^ → [thioredoxin]-disulfide + H^+^ + NADPH **EC:1.8.1.9** | APEX1 (2), ESR1 (2), ISG15 (2), PDIA6 (2), RP2 (2), TRIM25 (2). | Nucleus (5), cytosol (5), extracellular (4), mitochondrion (4) | None |
| 55. Thioredoxin reductase 2, mitochondrial  (TXNRD2; Q9NNW7) | Involved in the control of reactive oxygen species levels and the regulation of mitochondrial redox homeostasis. Maintains thioredoxin in a reduced state. May play a role in redox-regulated cell signaling./ [thioredoxin]-dithiol + NADP^+^ → [thioredoxin]-disulfide + H^+^ + NADPH **EC:1.8.1.9** | GTF2H1 (3), PSMC3 (3), FOXR2 (2), NCOA6 (2), NCOR1 (2), PPP1R3C (2) | Mitochondrion (5), cytosol (5),  peroxisome (3) | None |
| 56. Thioredoxin reductase 3  (TXNRD3; Q86VQ6) | Displays thioredoxin reductase, glutaredoxin and glutathione reductase activities. Catalyzes disulfide bond isomerization. Promotes disulfide bond formation between GPX4 and various sperm proteins and may play a role in sperm maturation by promoting formation of sperm structural components./ [thioredoxin]-dithiol + NADP^+^ → [thioredoxin]-disulfide + H^+^ + NADPH **EC:1.8.1.9** | None | Mitochondrion (4), nucleus (4),  endoplasmic reticulum (4), cytosol (4), extracellular (3) | None |
| 57. FAD-dependent oxidoreductase domain-containing protein 2  (FOXRED2; Q8IWF2). | Probable flavoprotein which may function in endoplasmic reticulum associated degradation (ERAD). May bind non-native proteins in the endoplasmic reticulum and target them to the ubiquitination machinery for subsequent degradation | SEL1L (4), OS9 (3), TXNDC16 (2) | Endoplasmic reticulum (5) | None |
| 58. FAD-linked sulfhydryl oxidase ALR  (GFER; P55789) | FAD-dependent sulfhydryl oxidase that regenerates the redox-active disulfide bonds in CHCHD4/MIA40, a chaperone essential for disulfide bond formation and protein folding in the mitochondrial intermembrane space. The reduced form of CHCHD4/MIA40 forms a transient intermolecular disulfide bridge with GFER/ERV1, resulting in regeneration of the essential disulfide bonds in CHCHD4/MIA40, while GFER/ERV1 becomes re-oxidized by donating electrons to cytochrome c or molecular oxygen./ O_2_ + 2 R'C(R)SH → H_2_O_2_ + R'C(R)S-S(R)CR' **EC:1.8.3.2** | COPS5 (6), BNIPL (4), AIFM1 (2), AMMECR1 (2), CDK4 (2), KLHL20 (2), PGPEP1 (2), TUBB2A (2) | Extracellular (5), mitochondrion (5),  cytosol (5) | None |
| 59. Prenylcysteine oxidase 1 (PCYOX1;Q9UHG3) | Involved in the degradation of prenylated proteins. Cleaves the thioether bond of prenyl-L-cysteines, such as farnesylcysteine and geranylgeranylcysteine./ an S-prenyl-L-cysteine + H_2_O + O_2_ → a prenal + H_2_O_2_ + L-cysteine **EC:1.8.3.5** | ANTXR1 (2), BLVRA (2), DLK1 (2), PLAUR (2), UBAC2 (2) | Lysosome (5), extracellular (4) | None |
| 60.Ribosyldihydronicotinamide dehydrogenase [quinone] (NQO2; P16083) | The enzyme apparently serves as a quinone reductase in connection with conjugation reactions of hydroquinones involved in detoxification pathways as well as in biosynthetic processes such as the vitamin K-dependent gamma-carboxylation of glutamate residues in prothrombin synthesis./ 1-(β-D-ribofuranosyl)-1,4-dihydronicotinamide + a quinone + H^+^ → a quinol + β-nicotinamide D-riboside **EC:1.10.5.1** | GORASP2 (2), LRRC7 (2) | Nucleus (5), cytosol (5), extracellular (4) | 4qod. |
| 61. Dimethylaniline monooxygenase [N-oxide-forming] 1(FMO1; Q01740) | This protein is involved in the oxidative metabolism of a variety of xenobiotics such as drugs and pesticides. Form I catalyzes the N-oxygenation of secondary and tertiary amines.  / H^+^ + *N,N*-dimethylaniline + NADPH + O_2_ → H_2_O + *N,N*-dimethylaniline *N*-oxide + NADP^+^ **EC:1.14.13.8** | None | Endoplasmic reticulum (4),  peroxisome (3) | None |
| 62. Dimethylaniline monooxygenase [N-oxide-forming] 2 (FMO2; Q99518) | Catalyzes the N-oxidation of certain primary alkylamines to their oximes via an N-hydroxylamine intermediate. Inactive toward certain tertiary amines, such as imipramine or chloropromazine. Can catalyze the S-oxidation of methimazole./ H^+^ + *N,N*-dimethylaniline + NADPH + O_2_ → H_2_O + *N,N*-dimethylaniline *N*-oxide + NADP^+^ **EC:1.14.13.8** | None | Endoplasmic reticulum (4) | None |
| 63. Dimethylaniline monooxygenase [N-oxide-forming] 3 (FMO3; P31513) | Essential hepatic enzyme that catalyzes the oxygenation of a wide variety of nitrogen- and sulfur-containing compounds including drugs as well as dietary compounds. Plays an important role in the metabolism of trimethylamine (TMA), via the production of trimethylamine N-oxide (TMAO) metabolite. TMA is generated by the action of gut microbiota using dietary precursors such as choline, choline containing compounds, betaine or L-carnitine. By regulating TMAO concentration, FMO3 directly impacts both platelet responsiveness and rate of thrombus formation./ H^+^ + *N,N*-dimethylaniline + NADPH + O_2_ → H_2_O + *N,N*-dimethylaniline N-oxide + NADP^+^ **EC:1.14.13.8**; NADPH + O_2_ + trimethylamine → H_2_O + NADP^+^ + trimethylamine N-oxide **EC:1.14.13.148**; fenbendazole + O_2_ + reduced [NADPH—hemoprotein reductase] → fenbendazole S-oxide + H+ + H_2_O + oxidized [NADPH—hemoprotein reductase] **EC:1.14.14.73;** albendazole + O_2_ + reduced [NADPH—hemoprotein reductase] → albendazole S-oxide + H^+^ + H_2_O + oxidized [NADPH—hemoprotein reductase] **EC:1.14.14.73**; (*S*)-nicotine + NADPH + O_2_ → H_2_O + NADP^+^ + trans-*(S*)-nicotine *N*^1'^-oxide | None | Endoplasmic reticulum (4), cytosol (4),  plasma membrane (3), nucleus (3) | None |
| 64. Dimethylaniline monooxygenase [N-oxide-forming] 4 (FMO4; P31512) | This protein is involved in the oxidative metabolism of a variety of xenobiotics such as drugs and pesticides./ H^+^ + *N,N*-dimethylaniline + NADPH + O_2_ → H_2_O + *N,N*-dimethylaniline *N*-oxide + NADP^+^ **EC:1.14.13.8** | None | Endoplasmic reticulum (3) | None |
| 65. Flavin-containing monooxygenase 5 (FMO5;P49326) | Acts as Baeyer-Villiger monooxygenase on a broad range of substrates. Catalyzes the insertion of an oxygen atom into a carbon-carbon bond adjacent to a carbonyl, which converts ketones to esters. Active on diverse carbonyl compounds, whereas soft nucleophiles are mostly non- or poorly reactive. In contrast with other forms of FMO it is non- or poorly active on 'classical' substrates such as drugs, pesticides, and dietary components containing soft nucleophilic heteroatoms (Probable). Able to oxidize drug molecules bearing a carbonyl group on an aliphatic chain, such as nabumetone and pentoxifylline. Also, in the absence of substrates, shows slow but yet significant NADPH oxidase activity. Acts as a positive modulator of cholesterol biosynthesis as well as glucose homeostasis, promoting metabolic aging via pleiotropic effects./ H^+^ + *N,N*-dimethylaniline + NADPH + O_2_ → H_2_O + *N,N*-dimethylaniline N-oxide + NADP^+^ **EC:1.14.13.8**; H^+^ + NADPH + O_2_ → H_2_O_2_ + NADP^+^ **EC:1.6.3.1**; H^+^ + heptan-2-one + NADPH + O_2_ → H_2_O + NADP^+^ + pentyl acetate; H^+^ + NADPH + O_2_ + octan-3-one → H_2_O + NADP^+^ + pentyl propanoate; H^+^ + NADPH + O_2_ + octan-3-one → ethyl hexanoate + H_2_O + NADP^+^; H^+^ + hexan-3-one + NADPH + O_2_ → ethyl butanoate + H_2_O + NADP^+^; H^+^ + hexan-3-one + NADPH + O_2_ → H_2_O + NADP^+^ + propyl propanoate; H^+^ + heptan-4-one + NADPH + O_2_ → H_2_O + NADP^+^ + propyl butanoate; geranial + H^+^ + NADPH + O_2_ → (1*E*)-2,6-dimethylhepta-1,5-dien-1-yl formate + H_2_O + NADP^+^; H^+^ + NADPH + O_2_ + sulcatone → 4-methylpent-3-en-1-yl acetate + H_2_O + NADP^+^. | None | Endoplasmic reticulum (5), cytosol (4) | None |
| 66. Kynurenine 3-monooxygenase (KMO;O15229) | Catalyzes the hydroxylation of L-kynurenine (L-Kyn) to form 3-hydroxy-L-kynurenine (L-3OHKyn). Required for synthesis of quinolinic acid, a neurotoxic NMDA receptor antagonist and potential endogenous inhibitor of NMDA receptor signaling in axonal targeting, synaptogenesis and apoptosis during brain development. Quinolinic acid may also affect NMDA receptor signaling in pancreatic beta cells, osteoblasts, myocardial cells, and the gastrointestinal tract (Probable)./ H^+^ + L-kynurenine + NADPH + O_2_ → 3-hydroxy-L-kynurenine + H_2_O + NADP^+^ **EC:1.14.13.9** | None | Mitochondrion (4), cytosol (4), plasma membrane (3), extracellular (3) | None |
| 67. Nitric oxide synthase, brain  (NOS1; P29475) | Produces nitric oxide (NO) which is a messenger molecule with diverse functions throughout the body. In the brain and peripheral nervous system, NO displays many properties of a neurotransmitter. Probably has nitrosylase activity and mediates cysteine S-nitrosylation of cytoplasmic target proteins such SRR./ H^+^ + 2 L-arginine + 3 NADPH + 4 O_2_ → 4 H_2_O + 2 L-citrulline + 3 NADP^+^ + 2 NO **EC:1.14.13.39** | DLG4 (5), PRKD1 (4), STUB1 (4). NOS1AP (3), SOX2 (2), ZDHHC23 (2) | Plasma membrane (5), nucleus (5)  cytoskeleton (4), mitochondrion (4),  endoplasmic reticulum (4), cytosol (4) | None |
| 68. Nitric oxide synthase, inducible (NOS2; P35228) | Produces nitric oxide (NO) which is a messenger molecule with diverse functions throughout the body. In macrophages, NO mediates tumoricidal and bactericidal actions. Also has nitrosylase activity and mediates cysteine S-nitrosylation of cytoplasmic target proteins such PTGS2/COX2. As component of the iNOS-S100A8/9 transnitrosylase complex involved in the selective inflammatory stimulus-dependent S-nitrosylation of GAPDH on 'Cys-247' implicated in regulation of the GAIT complex activity and probably multiple targets including ANXA5, EZR, MSN and VIM. Involved in inflammation, enhances the synthesis of proinflammatory mediators such as IL6 and IL8./ H^+^ + 2 L-arginine + 3 NADPH + 4 O_2_ → 4 H_2_O + 2 L-citrulline + 3 NADP^+^ + 2 NO **EC:1.14.13.39** | SPSB2 (9), CALM1 (5), SPSB1 (5), CAV1 (2), CUL5 (2), FBXO45 (2), HSP90AA1 (2), RAC2 (2), SPSB4 (2), UCHL5 (2) | Peroxisome (5), plasma membrane (4), nucleus (4), cytosol (4), extracellular (3), cytoskeleton (3), mitochondrion (3) | None |
| 69. Nitric oxide synthase, endotelial (NOS3; P29474) | Produces nitric oxide (NO) which is implicated in vascular smooth muscle relaxation through a cGMP-mediated signal transduction pathway. NO mediates vascular endothelial growth factor (VEGF)-induced angiogenesis in coronary vessels and promotes blood clotting through the activation of platelets./  H^+^ + 2 L-arginine + 3 NADPH + 4 O_2_ → 4 H_2_O + 2 L-citrulline + 3 NADP^+^ + 2 NO **EC:1.14.13.39** | HSP90AA1 (7), CALM1 (6), CAV1 (4), NOSIP (4), NOSTRIN (4), ACTB (3), AKT1 (3), CDC37 (3), APOE (2), APP (2), GCDH (2), GOLGA2 (2), GUCY1B3 (2), PPP2R4 (2), ST13 (2). | Plasma membrane (5), cytoskeleton (5), golgi apparatus (5), nucleus (4),  cytosol (4), extracellular (3), mitochondrion (3), endoplasmic reticulum (3) | None |
| 70. Squalene monooxygenase (SQLE; Q14534) | Catalyzes the stereospecific oxidation of squalene to (S)-2,3-epoxysqualene, and is considered to be a rate-limiting enzyme in steroid biosynthesis./ O_2_ + reduced [NADPH—hemoprotein reductase] + squalene → (*S*)-2,3-epoxysqualene + H^+^ + H_2_O + oxidized [NADPH—hemoprotein reductase] **EC:1.14.14.17** | FAF2 (2), MARCH6 (2), TREML2 (2) | Plasma membrane (4), endoplasmic reticulum (4), cytoskeleton (3), cytosol (3) | None |
| 71. Ubiquinone biosynthesis monooxygenase COQ6, mitochondrial (COQ6; Q9Y2Z9) | FAD-dependent monooxygenase required for the C5-ring hydroxylation during ubiquinone biosynthesis. Catalyzes the hydroxylation of 3-hexaprenyl-4-hydroxybenzoic acid (HHB) to 3-hexaprenyl-4,5-dihydroxybenzoic acid (DHHB). The electrons required for the hydroxylation reaction may be funneled indirectly from NADPH via a ferredoxin/ferredoxin reductase system to COQ6 (By similarity). Is able to perform the deamination reaction at C4 of 3-hexaprenyl-4-amino-5-hydroxybenzoic acid (HHAB) to produce DHHB when expressed in yeast cells lacking COQ9, even if utilization of para-aminobenzoic acid (pABA) involving C4-deamination seems not to occur in bacteria, plants and mammals, where only C5 hydroxylation of HHB has been shown./ 4-hydroxy-3-*all*-*trans*-hexaprenylbenzoate + 2 H^+^ + O_2_ + 2 reduced [2Fe-2S]-[ferredoxin] → 3,4-dihydroxy-5-*all*-*trans*-hexaprenylbenzoate + H_2_O + 2 oxidized [2Fe-2S]-[ferredoxin] | COQ3 (3), AGR2 (2), COQ5 (2), COQ9(2) | Mitochondrion (5), golgi apparatus (3) | None |
| 72. Metalloreductase STEAP3 (STEAP3; Q658P3) | Endosomal ferrireductase required for efficient transferrin-dependent iron uptake in erythroid cells. Participates in erythroid iron homeostasis by reducing Fe^3+^ to Fe^2+^. Can also reduce of Cu^2+^ to Cu^1+^, suggesting that it participates in copper homeostasis. Endosomal ferrireductase required for efficient transferrin-dependent iron uptake in erythroid cells. Participates in erythroid iron homeostasis by reducing Fe^3+^ to Fe^2+^. Can also reduce of Cu^2+^ to Cu^1+^, suggesting that it participates in copper homeostasis. | NRAS (3), BNIP3L (2), BSG (2), DLK1 (2), ENPP6 (2), FAM177A1 (2), HRAS (2), KRAS (2), NT5E (2), PKMYT1 (2), SLC39A4 (2), TEX29 (2), TMEFF1 (2) | Endosome (5), plasma membrane (4) | None |
| 73. Methionine synthase reductase (MTRR; Q9UBK8) | Key enzyme in methionine and folate homeostasis responsible for the reactivation of methionine synthase (MTR/MS) activity by catalyzing the reductive methylation of MTR-bound cob(II)alamin. Cobalamin (vitamin B12) forms a complex with MTR to serve as an intermediary in methyl transfer reactions that cycles between MTR-bound methylcob(III)alamin and MTR bound-cob(I)alamin forms, and occasional oxidative escape of the cob(I)alamin intermediate during the catalytic cycle leads to the inactive cob(II)alamin species. The processing of cobalamin in the cytosol occurs in a multiprotein complex composed of at least MMACHC, MMADHC, MTRR and MTR which may contribute to shuttle safely and efficiently cobalamin towards MTR in order to produce methionine. Also necessary for the utilization of methyl groups from the folate cycle, thereby affecting transgenerational epigenetic inheritance. Also acts as a molecular chaperone for methionine synthase by stabilizing apoMTR and incorporating methylcob(III)alamin into apoMTR to form the holoenzyme. Also serves as an aquacob(III)alamin reductase by reducing aquacob(III)alamin to cob(II)alamin; this reduction leads to stimulation of the conversion of apoMTR and aquacob(III)alamin to MTR holoenzyme./ 2 [methionine synthase]-methylcob(III)alamin + H^+^ + NADP^+^ + 2 *S*-adenosyl-L-homocysteine → 2 [methionine synthase]-cob(II)alamin + NADPH + 2 *S*-adenosyl-L-methionine **EC:1.16.1.8;**  2 cob(II)alamin + H^+^ + 2 H_2_O + NADP^+^ → 2 aquacob(III)alamin + NADPH | MTR (2) | Cytosol (4), nucleus (3) | None |
| 74. NADPH:adrenodoxin oxidoreductase, mitochondrial (FDXR; P22570) | Serves as the first electron transfer protein in all the mitochondrial P450 systems including cholesterol side chain cleavage in all steroidogenic tissues, steroid 11-beta hydroxylation in the adrenal cortex, 25-OH-vitamin D3-24 hydroxylation in the kidney, and sterol C-27 hydroxylation in the liver./ H^+^ + NADP^+^ + 2 reduced [adrenodoxin] → NADPH + 2 oxidized [adrenodoxin] **EC:1.18.1.6** | CBWD1 (2), TBC1D22A (2), THBS3 (2), TSSC1 (2), TTC39B (2) | Mitochondrion (5), nucleus (3) | None |
| 75. Cytochrome b-245 heavy chain (CYBB; P04839) | Critical component of the membrane-bound oxidase of phagocytes that generates superoxide. It is the terminal component of a respiratory chain that transfers single electrons from cytoplasmic NADPH across the plasma membrane to molecular oxygen on the exterior. Also functions as a voltage-gated proton channel that mediates the H^+^ currents of resting phagocytes. It participates in the regulation of cellular pH and is blocked by zinc. | IQGAP1 (2) | Plasma membrane (5), endoplasmic reticulum (5), nucleus (4), extracellular (3), mitochondrion (3), cytosol (3), golgi apparatus (3) | None |
| 76. Dual oxidase 1 (DUOX1; Q9NRD9) | Generates hydrogen peroxide which is required for the activity of thyroid peroxidase/TPO and lactoperoxidase/LPO. Plays a role in thyroid hormones synthesis and lactoperoxidase-mediated antimicrobial defense at the surface of mucosa. May have its own peroxidase activity through its N-terminal peroxidase-like domain./ H^+^ + NADH + O_2_ → H_2_O_2_ + NAD^+^ **EC:1.6.3.1;**  H^+^ + NADPH + O_2_ → H_2_O_2_ + NADP^+^ **EC:1.6.3.1** | None | Plasma membrane (5), endoplasmic reticulum (5), extracellular (3), mitochondrion (3),  cytosol (3) | None |
| 77. Dual oxidase 2 (DUOX2; Q9NRD8) | Generates hydrogen peroxide which is required for the activity of thyroid peroxidase/TPO and lactoperoxidase/LPO. Plays a role in thyroid hormones synthesis and lactoperoxidase-mediated antimicrobial defense at the surface of mucosa. May have its own peroxidase activity through its N-terminal peroxidase-like domain./ H^+^ + NADH + O_2_ → H_2_O_2_ + NAD^+^ **EC:1.6.3.1;**  H^+^ + NADPH + O_2_ → H_2_O_2_ + NADP^+^ **EC:1.6.3.1** | None | Plasma membrane (5), endoplasmic reticulum (5), cytosol (5), extracellular (4), mitochondrion (3) | None |
| 78. 2-hydroxyacyl-CoA lyase 2 (ILVBL; A1L0T0) | Endoplasmic reticulum 2-OH acyl-CoA lyase involved in the cleavage (C1 removal) reaction in the fatty acid alpha-oxydation in a thiamine pyrophosphate (TPP)-dependent manner. Involved in the phytosphingosine degradation pathway./ 2-hydroxyoctadecanoyl-CoA → formyl-CoA + heptadecanal  (2*R*)-hydroxyhexadecanoyl-CoA → formyl-CoA + pentadecanal | GBA (2) | Endoplasmic reticulum (5),  plasma membrane (3) | None |
| 79. Alkyldihydroxyacetonephosphate synthase, peroxisomal (AGPS; O00116) | Catalyzes the exchange of the acyl chain in acyl-dihydroxyacetonephosphate (acyl-DHAP) for a long chain fatty alcohol, yielding the first ether linked intermediate, i.e. alkyl-dihydroxyacetonephosphate (alkyl-DHAP), in the pathway of ether lipid biosynthesis./ A 1-acylglycerone 3-phosphate + a long chain fatty alcohol → 1-O-alkylglycerone 3-phosphate + a long-chain fatty acid + H^+^ **EC:2.5.1.26;**  1-hexadecanoylglycerone 3-phosphate + hexadecan-1-ol → 1-O-hexadecylglycerone 3-phosphate + H^+^ + hexadecanoate;  1-hexadecanoylglycerone 3-phosphate + a long-chain fatty acid → a 1-acylglycerone 3-phosphate + hexadecanoate | CAT (2), EMC2 (2), GNPAT (2), GORASP1 (2), PDHA1 (2), PEX5 (2) | Peroxisome (5), nucleus (5), cytosol (5), mitochondrion (4) | None |
| 80. Phosphopantothenoylcysteine decarboxylase (PPCDC; Q96CD2) | Necessary for the biosynthesis of coenzyme A. Catalyzes the decarboxylation of 4-phosphopantothenoylcysteine to form 4'-phosphopantotheine./ H^+^ + N-[(*R*)-4-phosphopantothenoyl]-L-cysteine → CO_2_ + D-pantetheine 4'-phosphate **EC:4.1.1.36** | ZNF232 (3), FOXR1 (2), TXN2 (2), WDYHV1 (2) | Cytosol (5), extracellular (3) | None |
| 81. Cryptochrome-1 (CRY1; Q16526) | Transcriptional repressor which forms a core component of the circadian clock. The circadian clock, an internal time-keeping system, regulates various physiological processes through the generation of approximately 24 hour circadian rhythms in gene expression, which are translated into rhythms in metabolism and behavior. It is derived from the Latin roots 'circa' (about) and 'diem' (day) and acts as an important regulator of a wide array of physiological functions including metabolism, sleep, body temperature, blood pressure, endocrine, immune, cardiovascular, and renal function. Consists of two major components: the central clock, residing in the suprachiasmatic nucleus (SCN) of the brain, and the peripheral clocks that are present in nearly every tissue and organ system. Both the central and peripheral clocks can be reset by environmental cues, also known as Zeitgebers (German for 'timegivers'). The predominant Zeitgeber for the central clock is light, which is sensed by retina and signals directly to the SCN. The central clock entrains the peripheral clocks through neuronal and hormonal signals, body temperature and feeding-related cues, aligning all clocks with the external light/dark cycle. Circadian rhythms allow an organism to achieve temporal homeostasis with its environment at the molecular level by regulating gene expression to create a peak of protein expression once every 24 hours to control when a particular physiological process is most active with respect to the solar day. Transcription and translation of core clock components (CLOCK, NPAS2, ARNTL/BMAL1, ARNTL2/BMAL2, PER1, PER2, PER3, CRY1 and CRY2) plays a critical role in rhythm generation, whereas delays imposed by post-translational modifications (PTMs) are important for determining the period (tau) of the rhythms (tau refers to the period of a rhythm and is the length, in time, of one complete cycle). A diurnal rhythm is synchronized with the day/night cycle, while the ultradian and infradian rhythms have a period shorter and longer than 24 hours, respectively. Disruptions in the circadian rhythms contribute to the pathology of cardiovascular diseases, cancer, metabolic syndromes and aging. A transcription/translation feedback loop (TTFL) forms the core of the molecular circadian clock mechanism. Transcription factors, CLOCK or NPAS2 and ARNTL/BMAL1 or ARNTL2/BMAL2, form the positive limb of the feedback loop, act in the form of a heterodimer and activate the transcription of core clock genes and clock-controlled genes (involved in key metabolic processes), harboring E-box elements (5'-CACGTG-3') within their promoters. The core clock genes: PER1/2/3 and CRY1/2 which are transcriptional repressors form the negative limb of the feedback loop and interact with the CLOCK\|NPAS2-ARNTL/BMAL1\|ARNTL2/BMAL2 heterodimer inhibiting its activity and thereby negatively regulating their own expression. This heterodimer also activates nuclear receptors NR1D1/2 and RORA/B/G, which form a second feedback loop and which activate and repress ARNTL/BMAL1 transcription, respectively. CRY1 and CRY2 have redundant functions but also differential and selective contributions at least in defining the pace of the SCN circadian clock and its circadian transcriptional outputs. More potent transcriptional repressor in cerebellum and liver than CRY2, though more effective in lengthening the period of the SCN oscillator. On its side, CRY2 seems to play a critical role in tuning SCN circadian period by opposing the action of CRY1. With CRY2, is dispensable for circadian rhythm generation but necessary for the development of intercellular networks for rhythm synchrony. Capable of translocating circadian clock core proteins such as PER proteins to the nucleus. Interacts with CLOCK-ARNTL/BMAL1 independently of PER proteins and is found at CLOCK-ARNTL/BMAL1-bound sites, suggesting that CRY may act as a molecular gatekeeper to maintain CLOCK-ARNTL/BMAL1 in a poised and repressed state until the proper time for transcriptional activation. Represses the CLOCK-ARNTL/BMAL1 induced transcription of BHLHE40/DEC1. Represses the CLOCK-ARNTL/BMAL1 induced transcription of ATF4, MTA1, KLF10 and NAMPT. May repress circadian target genes expression in collaboration with HDAC1 and HDAC2 through histone deacetylation. Mediates the clock-control activation of ATR and modulates ATR-mediated DNA damage checkpoint. In liver, mediates circadian regulation of cAMP signaling and gluconeogenesis by binding to membrane-coupled G proteins and blocking glucagon-mediated increases in intracellular cAMP concentrations and CREB1 phosphorylation. Inhibits hepatic gluconeogenesis by decreasing nuclear FOXO1 levels that downregulates gluconeogenic gene expression. Besides its role in the maintenance of the circadian clock, is also involved in the regulation of other processes. Represses glucocorticoid receptor NR3C1/GR-induced transcriptional activity by binding to glucocorticoid response elements (GREs). Plays a key role in glucose and lipid metabolism modulation, in part, through the transcriptional regulation of genes involved in these pathways, such as LEP or ACSL4. Represses PPARD and its target genes in the skeletal muscle and limits exercise capacity. Plays an essential role in the generation of circadian rhythms in the retina. Represses the transcriptional activity of NR1I2. | FBXL3 (8), PER2 (7), PER1 (5), ARNTL (4), CSNK1E (4), CUL1 (3), FOXO1 (3), SKP1 (3), USP7 (3), AP2M1 (2), CRY2 (2), CSNK2A1 (2), CSNK2B (2), PLSCR1 (2), PPP2R1B (2), QPRT (2), TEKT4 (2), USP2 (2), XPO1 (2) | Nucleus (5), cytoskeleton (3), mitochondrion (3), cytosol (3) | None |
| 82. Cryptochrome-2 (CRY2; Q49AN0) | Transcriptional repressor which forms a core component of the circadian clock. The circadian clock, an internal time-keeping system, regulates various physiological processes through the generation of approximately 24 hour circadian rhythms in gene expression, which are translated into rhythms in metabolism and behavior. It is derived from the Latin roots 'circa' (about) and 'diem' (day) and acts as an important regulator of a wide array of physiological functions including metabolism, sleep, body temperature, blood pressure, endocrine, immune, cardiovascular, and renal function. Consists of two major components: the central clock, residing in the suprachiasmatic nucleus (SCN) of the brain, and the peripheral clocks that are present in nearly every tissue and organ system. Both the central and peripheral clocks can be reset by environmental cues, also known as Zeitgebers (German for 'timegivers'). The predominant Zeitgeber for the central clock is light, which is sensed by retina and signals directly to the SCN. The central clock entrains the peripheral clocks through neuronal and hormonal signals, body temperature and feeding-related cues, aligning all clocks with the external light/dark cycle. Circadian rhythms allow an organism to achieve temporal homeostasis with its environment at the molecular level by regulating gene expression to create a peak of protein expression once every 24 hours to control when a particular physiological process is most active with respect to the solar day. Transcription and translation of core clock components (CLOCK, NPAS2, ARNTL/BMAL1, ARNTL2/BMAL2, PER1, PER2, PER3, CRY1 and CRY2) plays a critical role in rhythm generation, whereas delays imposed by post-translational modifications (PTMs) are important for determining the period (tau) of the rhythms (tau refers to the period of a rhythm and is the length, in time, of one complete cycle). A diurnal rhythm is synchronized with the day/night cycle, while the ultradian and infradian rhythms have a period shorter and longer than 24 hours, respectively. Disruptions in the circadian rhythms contribute to the pathology of cardiovascular diseases, cancer, metabolic syndromes and aging. A transcription/translation feedback loop (TTFL) forms the core of the molecular circadian clock mechanism. Transcription factors, CLOCK or NPAS2 and ARNTL/BMAL1 or ARNTL2/BMAL2, form the positive limb of the feedback loop, act in the form of a heterodimer and activate the transcription of core clock genes and clock-controlled genes (involved in key metabolic processes), harboring E-box elements (5'-CACGTG-3') within their promoters. The core clock genes: PER1/2/3 and CRY1/2 which are transcriptional repressors form the negative limb of the feedback loop and interact with the CLOCK\|NPAS2-ARNTL/BMAL1\|ARNTL2/BMAL2 heterodimer inhibiting its activity and thereby negatively regulating their own expression. This heterodimer also activates nuclear receptors NR1D1/2 and RORA/B/G, which form a second feedback loop and which activate and repress ARNTL/BMAL1 transcription, respectively. CRY1 and CRY2 have redundant functions but also differential and selective contributions at least in defining the pace of the SCN circadian clock and its circadian transcriptional outputs. Less potent transcriptional repressor in cerebellum and liver than CRY1, though less effective in lengthening the period of the SCN oscillator. Seems to play a critical role in tuning SCN circadian period by opposing the action of CRY1. With CRY1, dispensable for circadian rhythm generation but necessary for the development of intercellular networks for rhythm synchrony. May mediate circadian regulation of cAMP signaling and gluconeogenesis by blocking glucagon-mediated increases in intracellular cAMP concentrations and in CREB1 phosphorylation. Besides its role in the maintenance of the circadian clock, is also involved in the regulation of other processes. Plays a key role in glucose and lipid metabolism modulation, in part, through the transcriptional regulation of genes involved in these pathways, such as LEP or ACSL4. Represses glucocorticoid receptor NR3C1/GR-induced transcriptional activity by binding to glucocorticoid response elements (GREs). Represses the CLOCK-ARNTL/BMAL1 induced transcription of BHLHE40/DEC1. Represses the CLOCK-ARNTL/BMAL1 induced transcription of NAMPT. Represses PPARD and its target genes in the skeletal muscle and limits exercise capacity. Represses the transcriptional activity of NR1I2. | FBXL3 (10), PER2 (4), PER1 (3), AP2M1 (2), ARNTL (2), CLOCK (2), CRY1 (2), CSNK1E (2), CSNK2B (2), CUL1 (2), DDB1 (2), MTUS2 (2), PDE9A (2), PPP5C (2), QPRT (2), SKP1 (2), TEKT4 (2), XPO1 (2), XRN2 (2) | Extracellular (5), nucleus (5), cytosol (5) | None |
| 83. Apoptosis-inducing factor 1, mitochondrial (AIFM1; O95831) | Functions both as NADH oxidoreductase and as regulator of apoptosis. In response to apoptotic stimuli, it is released from the mitochondrion intermembrane space into the cytosol and to the nucleus, where it functions as a proapoptotic factor in a caspase-independent pathway. The soluble form (AIFsol) found in the nucleus induces 'parthanatos' i.e. caspase-independent fragmentation of chromosomal DNA (By similarity). Binds to DNA in a sequence-independent manner. Interacts with EIF3G, and thereby inhibits the EIF3 machinery and protein synthesis, and activates caspase-7 to amplify apoptosis. Plays a critical role in caspase-independent, pyknotic cell death in hydrogen peroxide-exposed cells. In contrast, participates in normal mitochondrial metabolism. Plays an important role in the regulation of respiratory chain biogenesis by interacting with CHCHD4 and controlling CHCHD4 mitochondrial import./ A + H^+^ + NADH → AH_2_ + NAD^+^ | AK2 (3), ARAF (3), BAG6 (3), CHCHD4 (3), EIF3G (3), HSPA1A (3), MLF2 (3), NDUFS5 (3), PGAM5 (3), RHOT2 (3), TOMM40 (3), TOR1AIP1 (3), APOOL (2), ATP5C1 (2), CFTR (2), CLPB (2), CLPP (2), CLPX (2), COX2 (2), COX4I1 (2), COX6B1 (2), CPOX (2), DLD (2), DNAJC11 (2), ENDOG (2), ERAL1 (2), GFER (2), GRPEL1 (2), HAX1 (2), HSD17B10 (2), HTRA2 (2), IARS2 (2), ILK (2), IMMT (2), LACTB (2), MLH1 (2), MTHFD1L (2), NDUFA13 (2), NDUFA8 (2), OMA1 (2), PARK2 (2), PHB (2), POLDIP2 (2), SAMM50 (2), SCO2 (2), SEC16A (2), SLC25A12 (2), SLC25A5 (2), TIMM13 (2), TIMM8A (2), TIMM8B (2), TSC22D4 (2), TTC19 (2), TUFM (2), TXN (2), XIAP (2) | Mitochondrion (5), nucleus (5), cytosol (4) | None |
| 84. Ferroptosis suppressor protein 1 (AIFM2; Q9BRQ8) | A NAD(P)H-dependent oxidoreductase involved in cellular oxidative stress response. At the plasma membrane, catalyzes reduction of coenzyme Q/ubiquinone-10 to ubiquinol-10, a lipophilic radical-trapping antioxidant that prevents lipid oxidative damage and consequently ferroptosis. Cooperates with GPX4 to suppress phospholipid peroxidation and ferroptosis. This anti-ferroptotic function is independent of cellular glutathione levels. May play a role in mitochondrial stress signaling. Upon oxidative stress, associates with the lipid peroxidation end product 4-hydroxy-2-nonenal (HNE) forming a lipid adduct devoid of oxidoreductase activity, which then translocates from mitochondria into the nucleus triggering DNA damage and cell death. Capable of DNA binding in a non-sequence specific way./ H^+^ + NADH + ubiquinone-10 → NAD^+^ + ubiquinol-10 | None. | Plasma membrane (5), mitochondrion (5), cytosol (5), extracellular (4), nucleus (4). | None |
| 85. Iodotyrosine deiodinase 1 (IYD; Q6PHW0) | Catalyzes the oxidative NADPH-dependent deiodination of monoiodotyrosine (L-MIT) or diiodotyrosine (L-DIT). Acts during the hydrolysis of thyroglobulin to liberate iodide, which can then reenter the hormone-producing pathways. Acts more efficiently on monoiodotyrosine than on diiodotyrosine./  2 iodide + L-tyrosine + 2 NADP^+^ → 3,5-diiodo-L-tyrosine + H+ + 2 NADPH **EC:1.21.1.1** | DDRGK1 (2), TRIM69 (2) | Plasma membrane (5),  nucleus (4) | None |
| 86. [F-actin]-monooxygenase MICAL1 (MICAL1; Q8TDZ2) | Monooxygenase that promotes depolymerization of F-actin by mediating oxidation of specific methionine residues on actin to form methionine-sulfoxide, resulting in actin filament disassembly and preventing repolymerization. In the absence of actin, it also functions as a NADPH oxidase producing H_2_O_2_. Acts as a cytoskeletal regulator that connects NEDD9 to intermediate filaments. Also acts as a negative regulator of apoptosis via its interaction with STK38 and STK38L; acts by antagonizing STK38 and STK38L activation by MST1/STK4. Involved in regulation of lamina-specific connectivity in the nervous system such as the development of lamina-restricted hippocampal connections. Through redox regulation of the actin cytoskeleton controls the intracellular distribution of secretory vesicles containing L1/neurofascin/NgCAM family proteins in neurons, thereby regulating their cell surface levels. May act as Rab effector protein and play a role in vesicle trafficking./ H^+^ + L-methionyl-[F-actin] + NADPH + O_2_ → H_2_O + L-methionyl-(*R*)-S-oxide-[F-actin] + NADP^+^ **EC:1.14.13.225** | EHD1 (2), NEDD9 (2), RAB1A (2), SLC25A41 (2), TAS2R7 (2), VIM (2) | Extracellular (5), cytoskeleton (5), cytosol (5), plasma membrane (4), nucleus (3) | None |
| 87. [F-actin]-monooxygenase MICAL2 (MICAL2; O94851) | Nuclear monooxygenase that promotes depolymerization of F-actin by mediating oxidation of specific methionine residues on actin to form methionine-sulfoxide, resulting in actin filament disassembly and preventing repolymerization. In the absence of actin, it also functions as a NADPH oxidase producing H_2_O_2_. Acts as a key regulator of the SRF signaling pathway elicited by nerve growth factor and serum: mediates oxidation and subsequent depolymerization of nuclear actin, leading to increase MKL1/MRTF-A presence in the nucleus and promote SRF:MKL1/MRTF-A-dependent gene transcription. Does not activate SRF:MKL1/MRTF-A through RhoA./ H^+^ + L-methionyl-[F-actin] + NADPH + O_2_ → H_2_O + L-methionyl-(R)-S-oxide-[F-actin] + NADP^+^  **EC:1.14.13.225** | None | Nucleus (5), cytoskeleton (4), cytosol (3) | None |
| 88. [F-actin]-monooxygenase MICAL3 (MICAL3; Q7RTP6) | Monooxygenase that promotes depolymerization of F-actin by mediating oxidation of specific methionine residues on actin to form methionine-sulfoxide, resulting in actin filament disassembly and preventing repolymerization. In the absence of actin, it also functions as a NADPH oxidase producing H_2_O_2_. Seems to act as Rab effector protein and plays a role in vesicle trafficking. Involved in exocytic vesicles tethering and fusion: the monooxygenase activity is required for this process and implicates RAB8A associated with exocytotic vesicles. Required for cytokinesis. Contributes to stabilization and/or maturation of the intercellular bridge independently of its monooxygenase activity. Promotes recruitment of Rab8 and ERC1 to the intercellular bridge, and together these proteins are proposed to function in timely abscission./ H^+^ + L-methionyl-[F-actin] + NADPH + O_2_ → H_2_O + L-methionyl-(R)-S-oxide-[F-actin] + NADP^+^ **EC:1.14.13.225** | KIF23 (2), NINL (2) | Nucleus (5), cytosol (5), plasma membrane (4), extracellular (4), cytoskeleton (4) | None |
| 89. FAD-dependent oxidoreductase domain-containing protein 1 (FOXRED1; Q96CU9) | Required for the assembly of the mitochondrial membrane respiratory chain NADH dehydrogenase (Complex I). Involved in mid-late stages of complex I assembly. | CLPP (2), HSPD1 (2), MTIF2 (2), NDUFS5 (2) | Mitochondrion (5) | None |
| 90 Solute carrier family 52, riboflavin transporter, member 1 (SLC52A1; Q9NWF4) | Plasma membrane transporter mediating the uptake by cells of the water soluble vitamin B2/riboflavin that plays a key role in biochemical oxidation-reduction reactions of the carbohydrate, lipid, and amino acid metabolism. Humans are unable to synthesize vitamin B2/riboflavin and must obtain it via intestinal absorption. | None | Plasma membrane (5) | None |
| 91. Solute carrier family 52, riboflavin transporter, member 2 (SLC52A2; Q9HAB3). | Plasma membrane transporter mediating the uptake by cells of the water soluble vitamin B2/riboflavin that plays a key role in biochemical oxidation-reduction reactions of the carbohydrate, lipid, and amino acid metabolism. Humans are unable to synthesize vitamin B2/riboflavin and must obtain it via intestinal absorption. May also act as a receptor for 4-hydroxybutyrate. | ADRB2 (2) | Plasma membrane (5) | None |
| 92. Solute carrier family 52, riboflavin transporter, member 3 (SLC52A3; Q9NQ40). | Plasma membrane transporter mediating the uptake by cells of the water soluble vitamin B2/riboflavin that plays a key role in biochemical oxidation-reduction reactions of the carbohydrate, lipid, and amino acid metabolism. Humans are unable to synthesize vitamin B2/riboflavin and must obtain it via intestinal absorption | None | Plasma membrane (5), nucleus (5) | None |
| 93. Flavin reductase (NADPH)  (BLVRB; P30043). | Broad specificity oxidoreductase that catalyzes the NADPH-dependent reduction of a variety of flavins, such as riboflavin, FAD or FMN, biliverdins, methemoglobin and PQQ (pyrroloquinoline quinone). Contributes to heme catabolism and metabolizes linear tetrapyrroles. Can also reduce the complexed Fe^3+^ iron to Fe^2+^ in the presence of FMN and NADPH. In the liver, converts biliverdin to bilirubin./ NADP^+^ + reduced riboflavin → 2 H^+^ + NADPH + riboflavin  **EC:1.5.1.30;** bilirubin IXα + NAD^+^ → biliverdin IXα + H^+^ + NADH **EC:1.3.1.24;** bilirubin IXα + NADP^+^ → biliverdin IXα + H^+^ + NADPH **EC:1.3.1.24** | ARL6IP6 (2), FCGR2A (2), FSD1 (2), NENF (2) | Plasma membrane (5), nucleus (5), cytosol (5), extracellular (4) | 5OOG; 5OOH |
| 94. Riboflavin kinase (RFK; Q969G6) | Catalyzes the phosphorylation of riboflavin (vitamin B2) to form flavin-mononucleotide (FMN), hence rate-limiting enzyme in the synthesis of FAD. Essential for TNF-induced reactive oxygen species (ROS) production. Through its interaction with both TNFRSF1A and CYBA, physically and functionally couples TNFRSF1A to NADPH oxidase. TNF-activation of RFK may enhance the incorporation of FAD in NADPH oxidase, a critical step for the assembly and activation of NADPH oxidase./  ATP + riboflavin → ADP + FMN + H^+^ **EC:2.7.1.26** | TNFRSF1A (2) | Cytosol (5), golgi apparatus (3) | None |
| 95. FAD synthase (FLAD1; Q8NFF5) | Catalyzes the adenylation of flavin mononucleotide (FMN) to form flavin adenine dinucleotide (FAD) coenzyme./ ATP + FMN + H^+^ → diphosphate + FAD **EC:2.7.7.2** | CDKN1A (2), KLHL14 (2), PLEKHO2 (2), PRTFDC1 (2), REL (2), TIM23 (2) | Cytosol (5), plasma membrane (4), mitochondrion (4) | None |

**Table S2. FoldX structure-based calculation of the stability effects (ΔΔG) due to phosphorylation and disease-associated missense mutations on human flavoproteins.** Flavoproteins were selected from the set analyzed by Vabulas and coworkers in terms of flavin-dependent proteasomal degradation [[4](#_ENREF_4)]. For each protein, the gene and protein name, UniProt code and the structural model used (PDB code) is indicated. Phosphorylation sites were retrieved from PhosphoSitePlus® (<https://www.phosphosite.org/>). Those sites indicated with an asterisk (*) denoted sites identified in at least 3 high-throughput proteomic reports (HTPs). Disease-associated variants were retrieved from OMIM (<https://www.omim.org/>), UniProt (<https://www.uniprot.org/>) or ClinVar (<https://www.ncbi.nlm.nih.gov/clinvar/>) databases.

| **Protein (PDB code)** | **Type** | **Variant/site** | **ΔΔG (kcal·mol^-1^)** | **ASA (%)** |
| --- | --- | --- | --- | --- |
| **CYBR3**  **(1UMK)** | Phosphorylation | T31 | 0.37 | 100 |
|  | Phosphorylation | S38 | 0.71 | 30.2 |
|  | Phosphorylation | S66 | 1.16 | 32.6 |
|  | Phosphorylation | S82(*) | 3.64 | 0 |
|  | Phosphorylation | T95 | 6.57 | 4.1 |
|  | Phosphorylation | S146 | 0.31 | 34.1 |
|  | Phosphorylation | T171 | -0.10 | 67.9 |
|  | Phosphorylation | S174(*) | 1.90 | 1 |
|  | Phosphorylation | T182 | 0.07 | 46.3 |
|  | Phosphorylation | T185 | 5.98 | 8 |
|  | Phosphorylation | Y43 | 1.31 | 15 |
|  | Phosphorylation | Y80 | -0.84 | 28 |
|  | Phosphorylation | Y248 | 0.20 | 26 |
|  | Disease-associated | S128P | -1.71 | 16.9 |
|  | Disease-associated | R58Q | 2.48 | 9.4 |
|  | Disease-associated | L149P | 7.90 | 0.2 |
|  | Disease-associated | V106M | 2.59 | 0.2 |
|  | Disease-associated | C204R | 10.49 | 0 |
|  | Disease-associated | L73P | 7.56 | 4.3 |
| **ACAD8 (1RXO)** | **Type** | **Variant/site** | **ΔΔG (kcal·mol^-1^)** | **ASA (%)** |
|  | Phosphorylation | S216 | 44.32 | 5 |
|  | Phosphorylation | S261(*) | -2.76 | 8 |
|  | Disease-associated | M128I | 3.52 | 4.6 |
|  | Disease-associated | D134Y | 13.90 | 3.4 |
|  | Disease-associated | G137R | 60.41 | 14.9 |
|  | Disease-associated | M152T | 29.46 | 0.1 |
|  | Disease-associated | V203I | 5.90 | 0.1 |
|  | Disease-associated | R302Q | 7.16 | 19.6 |
|  | Disease-associated | A320T | 15.02 | 0 |
|  | Disease-associated | Q385R | 35.20 | 1.5 |
| **ETFE (1EFV)** | **Type** | **Variant/site** | **ΔΔG (kcal·mol^-1^)** | **ASA (%)** |
|  | Phosphorylation | S65 | 6.79 | 0.3 |
|  | Phosphorylation | T172 | 4.60 | 20.7 |
|  | Phosphorylation | T182(*) | 10.04 | 0.6 |
|  | Phosphorylation | T194(*) | -0.32 | 69.7 |
|  | Phosphorylation | Y192(*) | -0.19 | 95 |
|  | Disease-associated | R164Q | 2.78 | 5.8 |
|  | Disease-associated | D128N | 0.46 | 14.1 |
| **ACADM (1EGD)** | **Type** | **Variant/site** | **ΔΔG (kcal·mol^-1^)** | **ASA (%)** |
|  | Phosphorylation | T70 | 1.45 | 51.3 |
|  | Phosphorylation | S207 | 17.76 | 14.9 |
|  | Phosphorylation | T228 | 16.84 | 11.7 |
|  | Phosphorylation | T300 | 45.11 | 0 |
|  | Phosphorylation | S320 | 8.09 | 14.9 |
|  | Phosphorylation | T351(*) | 15.16 | 5.3 |
|  | Phosphorylation | Y73 | 23.59 | 10.4 |
|  | Phosphorylation | Y183 | 11.14 | 2.2 |
|  | Phosphorylation | Y352 | 16.96 | 14.2 |
|  | Phosphorylation | Y353 | 8.15 | 17.8 |
|  | Phosphorylation | Y400(*) | 18.08 | 28.6 |
|  | Disease-associated | K329E | 4.81 | 31.9 |
|  | Disease-associated | R53C | 6.94 | 53.5 |
|  | Disease-associated | G267R | 9.80 | 12.7 |
|  | Disease-associated | I375R | 26.74 | 0 |
|  | Disease-associated | C244R | 26.81 | 1 |
|  | Disease-associated | M149I | 5.01 | 0 |
| **ACADSB (2JIF)** | **Type** | **Variant/site** | **ΔΔG (kcal·mol^-1^)** | **ASA (%)** |
|  | Phosphorylation | S81 | 0.07 | 0 |
|  | Phosphorylation | T82 | -0.01 | 55.7 |
|  | Phosphorylation | S183 | 0.02 | 31.6 |
|  | Phosphorylation | S185 | 6.05 | 24.2 |
|  | Phosphorylation | S289 | 33.85 | 1.1 |
|  | Phosphorylation | Y198(*) | 3.55 | 57.2 |
|  | Phosphorylation | Y199(*) | 8.68 | 7.7 |
|  | Phosphorylation | Y372 | 9.15 | 9.7 |
|  | Phosphorylation | Y373 | 57.70 | 9.3 |
|  | Phosphorylation | Y413(*) | 35.85 | 18.7 |
|  | Disease-associated | L255F | 17.20 | 2.4 |
|  | Disease-associated | I316V | 5.41 | 0 |
| **IVD (1IVH)** | **Type** | **Variant/site** | **ΔΔG (kcal·mol^-1^)** | **ASA (%)** |
|  | Phosphorylation | S158 | 4.62 | 49.9 |
|  | Phosphorylation | T214(*) | 27.58 | 3.7 |
|  | Phosphorylation | S222(*) | -2.69 | 37.9 |
|  | Phosphorylation | T334 | 22.12 | 1.7 |
|  | Phosphorylation | Y161 | 2.93 | 32.3 |
|  | Phosphorylation | Y277 | -3.98 | 71.9 |
|  | Phosphorylation | Y333 | 15.75 | 8.3 |
|  | Phosphorylation | Y344 | 51.97 | 0 |
|  | Disease-associated | L13P | 16.42 | 0.3 |
|  | Disease-associated | G170V | 40.29 | 1.6 |
|  | Disease-associated | R21C | 10.68 | 7.9 |
|  | Disease-associated | A282V | -2.69 | 18.8 |
|  | Disease-associated | G123R | 33.37 | 51.6 |
|  | Disease-associated | V314A | 8.57 | 0.4 |
| **AIFM1 (5KVI)** | **Type** | **Variant/site** | **ΔΔG (kcal·mol^-1^)** | **ASA (%)** |
|  | Phosphorylation | S176 | 5.84 | 5.1 |
|  | Phosphorylation | S182 | 3.47 | 5.4 |
|  | Phosphorylation | T188 | 0.82 | 29.8 |
|  | Phosphorylation | T190 | 0.22 | 43.8 |
|  | Phosphorylation | T263 | 0.53 | 63.3 |
|  | Phosphorylation | S266(*) | 0.98 | 28.1 |
|  | Phosphorylation | S268(*) | -0.70 | 82.9 |
|  | Phosphorylation | S279(*) | 0.91 | 63 |
|  | Phosphorylation | S292(*) | -0.02 | 58.9 |
|  | Phosphorylation | T328 | 6.40 | 13.6 |
|  | Phosphorylation | S371(*) | 0.31 | 58.7 |
|  | Phosphorylation | S375(*) | -0.25 | 55.8 |
|  | Phosphorylation | S376 | 0.43 | 81.2 |
|  | Phosphorylation | S416 | 0.21 | 100 |
|  | Phosphorylation | S519(*) | 1.26 | 23.3 |
|  | Phosphorylation | T521(*) | 14.33 | 0 |
|  | Phosphorylation | S524(*) | 4.29 | 15.2 |
|  | Phosphorylation | T526 | 6.27 | 17 |
|  | Phosphorylation | S530 | 3.13 | 5.6 |
|  | Phosphorylation | S532 | 2.94 | 1.5 |
|  | Phosphorylation | T542 | 0.79 | 45.5 |
|  | Phosphorylation | Y347 | 0.48 | 45.3 |
|  | Phosphorylation | Y443(*) | -1.40 | 60.9 |
|  | Disease-associated | G308E | 5.04 | 4.3 |
|  | Disease-associated | G360R | 3.44 | 53.1 |
|  | Disease-associated | T260A | 1.38 | 12.2 |
|  | Disease-associated | E493V | -2.48 | 0.3 |
| **ETFA (2A1U)** | **Type** | **Variant/site** | **ΔΔG (kcal·mol^-1^)** | **ASA (%)** |
|  | Phosphorylation | S32 | 0.59 | 82 |
|  | Phosphorylation | T37 | 13.59 | 1.1 |
|  | Phosphorylation | T42 | 3.32 | 31.3 |
|  | Phosphorylation | T45 | 1.22 | 45.4 |
|  | Phosphorylation | S52 | 4.67 | 2.4 |
|  | Phosphorylation | T93(*) | 4.00 | 2.5 |
|  | Phosphorylation | S140(*) | 1.02 | 46 |
|  | Phosphorylation | T143 | 2.27 | 17.2 |
|  | Phosphorylation | T171 | 3.24 | 16 |
|  | Phosphorylation | S172 | 1.49 | 14.2 |
|  | Phosphorylation | S179 | -0.48 | 98.7 |
|  | Phosphorylation | S190 | 0.10 | 87.3 |
|  | Phosphorylation | S192 | 0.15 | 100 |
|  | Phosphorylation | S207 | 1.04 | 22.7 |
|  | Phosphorylation | T213 | 1.94 | 31.7 |
|  | Phosphorylation | S227 | 0.27 | 45.5 |
|  | Phosphorylation | S248 | 0.36 | 14.8 |
|  | Phosphorylation | Y84(*) | 8.56 | 0.2 |
|  | Phosphorylation | Y149 | 1.90 | 14 |
|  | Phosphorylation | Y235(*) | -0.08 | 27.5 |
|  | Disease-associated | V157G | 4.08 | 8.2 |
|  | Disease-associated | T266M | -0.88 | 11.7 |
|  | Disease-associated | G116R | 11.94 | 0 |
|  | Disease-associated | T171I | -2.31 | 16 |
| **ACADS (2VIG)** | **Type** | **Variant/site** | **ΔΔG (kcal·mol^-1^)** | **ASA (%)** |
|  | Phosphorylation | S121 | 53.84 | 0.1 |
|  | Phosphorylation | T219 | 9.30 | 8.6 |
|  | Phosphorylation | T305 | 13.33 | 2 |
|  | Disease-associated | R46W | 7.30 | 31.1 |
|  | Disease-associated | R107C | 8.51 | 14 |
|  | Disease-associated | P55L | 7.72 | 55 |
| **PPOX (3NKS)** | **Type** | **Variant/site** | **ΔΔG (kcal·mol^-1^)** | **ASA (%)** |
|  | Phosphorylation | T242 | -0.25 | 72.8 |
|  | Phosphorylation | T473 | 0.97 | 83.8 |
|  | Disease-associated | G232R | 13.89 | 0 |
|  | Disease-associated | R59W | 2.02 | 9.1 |
|  | Disease-associated | R168C | 5.32 | 3.8 |
|  | Disease-associated | H20P | 4.79 | 17.9 |
|  | Disease-associated | D349A | 2.01 | 8.8 |
| **DLD (1ZMD)** | **Type** | **Variant/site** | **ΔΔG (kcal·mol^-1^)** | **ASA (%)** |
|  | Phosphorylation | T135(*) | 5.21 | 7.1 |
|  | Phosphorylation | S230 | 0.70 | 1.6 |
|  | Phosphorylation | T276 | 7.47 | 13.7 |
|  | Phosphorylation | S297(*) | -1.69 | 94 |
|  | Phosphorylation | T435 | 2.28 | 27.8 |
|  | Phosphorylation | S502 | 12.09 | 11.1 |
|  | Phosphorylation | Y153(*) | 2.12 | 34.1 |
|  | Disease-associated | K72E | 1.89 | 43.7 |
|  | Disease-associated | G229C | 10.51 | 0 |
|  | Disease-associated | P488L | 7.94 | 6.2 |
|  | Disease-associated | R495G | 7.21 | 14.1 |
|  | Disease-associated | E375K | -0.82 | 15 |
|  | Disease-associated | D479V | 5.00 | 39.6 |
| **ACADVL (2UXW)** | **Type** | **Variant/site** | **ΔΔG (kcal·mol^-1^)** | **ASA (%)** |
|  | Phosphorylation | S328 | 0.30 | 50.4 |
|  | Phosphorylation | S485 | 0.82 | 74.7 |
|  | Phosphorylation | S489(*) | 0.80 | 64.5 |
|  | Phosphorylation | S517 | 1.08 | 29.5 |
|  | Phosphorylation | S522(*) | -1.03 | 0 |
|  | Phosphorylation | S586 | 5.98 | 7.6 |
|  | Phosphorylation | Y631 | 5.18 | 9.6 |
|  | Disease-associated | K382Q | 1.33 | 26.6 |
|  | Disease-associated | R613W | 1.35 | 48.6 |
|  | Disease-associated | G401D | 6.11 | 0 |
|  | Disease-associated | A416T | 1.79 | 4.6 |
| **KDM1A (2DW4)** | **Type** | **Variant/site** | **ΔΔG (kcal·mol^-1^)** | **ASA (%)** |
|  | Phosphorylation | S172(*) | -0.21 | 79.5 |
|  | Phosphorylation | S299 | 2.7 | 12.6 |
|  | Phosphorylation | T305 | 3.12 | 3.0 |
|  | Phosphorylation | T335 | 1.73 | 37.9 |
|  | Phosphorylation | S346 | 3.23 | 11.3 |
|  | Phosphorylation | T588 | 3.32 | 5.4 |
|  | Phosphorylation | S611(*) | -0.01 | 83.5 |
|  | Phosphorylation | S683 | -0.03 | 74.5 |
|  | Phosphorylation | S687 | -0.12 | 61.5 |
|  | Disease-associated | E403K | -0.52 | 54.4 |
|  | Disease-associated | D580G | -0.87 | 59.9 |
|  | Disease-associated | Y785H | 1.0 | 24.7 |
| **POR (3QE2)** | **Type** | **Variant/site** | **ΔΔG (kcal·mol^-1^)** | **ASA (%)** |
|  | Phosphorylation | S68 | 2.78 | 27.6 |
|  | Phosphorylation | T251 | 0.90 | 82 |
|  | Phosphorylation | T525 | 0.04 | 48.2 |
|  | Phosphorylation | T526 | 0.60 | 10 |
|  | Phosphorylation | Y245 | 1.59 | 5.7 |
|  | Phosphorylation | Y259 | 1.38 | 14.3 |
|  | Phosphorylation | Y373(*) | -1.34 | 37.5 |
|  | Phosphorylation | Y374(*) | 21.15 | 0 |
|  | Phosphorylation | Y416 | 2.92 | 2.1 |
|  | Phosphorylation | Y564 | 14.35 | 0 |
|  | Phosphorylation | Y573 | 3.92 | 7.8 |
|  | Phosphorylation | Y575 | 6.96 | 1.6 |
|  | Phosphorylation | Y604 | -0.67 | 43.2 |
|  | Disease-associated | V492E | 2.17 | 3.4 |
|  | Disease-associated | A287P | 5.40 | 0 |
|  | Disease-associated | C569Y | 28.29 | 0.6 |
|  | Disease-associated | R457H | 0.36 | 31 |
|  | Disease-associated | G539R | 13.17 | 0.5 |
| **DHODH (6FMD)** | **Type** | **Variant/site** | **ΔΔG (kcal·mol^-1^)** | **ASA (%)** |
|  | Phosphorylation | T62 | 7.30 | 1.8 |
|  | Phosphorylation | S63 | 1.72 | 0 |
|  | Phosphorylation | T121 | 16.03 | 0 |
|  | Phosphorylation | S214 | 1.58 | 51 |
|  | Phosphorylation | T356 | 3.40 | 8.7 |
|  | Phosphorylation | T359 | 1.75 | 29.1 |
|  | Phosphorylation | Y146 | 4.11 | 7.4 |
|  | Phosphorylation | Y194 | 2.45 | 5.7 |
|  | Phosphorylation | Y355 | 0.96 | 26 |
|  | Disease-associated | G152R | 10.37 | 1.1 |
|  | Disease-associated | G202D | 10.46 | 0 |
|  | Disease-associated | R135C | 2.00 | 12 |
|  | Disease-associated | R346W | 5.44 | 21.6 |
| **MTHFR (6FCX)** | **Type** | **Variant/site** | **ΔΔG (kcal·mol^-1^)** | **ASA (%)** |
|  | Phosphorylation | T329 | 22.99 | 0 |
|  | Phosphorylation | T330 | 5.69 | 15.6 |
|  | Phosphorylation | T549 | 28.23 | 11.1 |
|  | Phosphorylation | Y90 | 27.12 | 0 |
|  | Disease-associated | A222V | 3.31 | 0 |
|  | Disease-associated | R335C | 2.04 | 55.5 |
|  | Disease-associated | W339G | 7.58 | 11.1 |
| **PNPO (6H00)** | **Type** | **Variant/site** | **ΔΔG (kcal·mol^-1^)** | **ASA (%)** |
|  | Phosphorylation | S164 | -2.83 | 89.2 |
|  | Phosphorylation | S165 | 0.58 | 52.7 |
|  | Phosphorylation | Y157(*) | 0.76 | 20.2 |
|  | Phosphorylation | Y212 | -2.46 | 44.1 |
|  | Disease-associated | R229Q | 6.27 | 20.7 |
|  | Disease-associated | R229W | 17.57 | 20.7 |
|  | Disease-associated | R225H | 1.94 | 91.5 |

**Table S3. Details of the HDX experiment and digestion metrics.**

| **Data Set** | **WT** | **S82D** | **P187S** |
| --- | --- | --- | --- |
| HDX reaction details | 50 mM K-HEPES, pD 7.4, 1 mM TCEP, 25 °C | 50 mM K-HEPES, pD 7.4, 1 mM TCEP, 25 °C | 50 mM K-HEPES, pD 7.4, 1 mM TCEP, 25 °C |
| HDX time course (sec) | 10, 30, 120, 300, 1200, 3600, 10800 | 10, 30, 120, 300, 1200, 3600, 10800 | 10, 30, 120, 300, 1200, 3600, 10800 |
| HDX control samples | fully deuterated WT NQO1 | fully deuterated S82D NQO1 | fully deuterated P187S NQO1 |
| Average back-exchange | 26.20% | 26.60% | 26.90% |
| # of Peptides | 139 | 139 | 136 |
| Sequence coverage | 99% | 99% | 99% |
| Average peptide length / Redundancy | 8.3/4.2 | 8.3/4.2 | 8.2/4.1 |
| Replicates (biological or technical) | duplicate of 10, 300, 10800 sec and FD | duplicate of 10, 300, 10800 sec and FD | duplicate of 10, 300, 10800 sec and FD |
| Repeatability (average SD) | 0.74% | 0.99% | 1.00% |

**Table S4. HDXMS data for all peptides characterized in this study.** In some cases, some *proteoforms* (i.e. WT, S82D or P187S) displayed additional peptides detected in these analyses; a, h and d indicate apo-protein, holo-protein and holo-protein with dicoumarol.

**
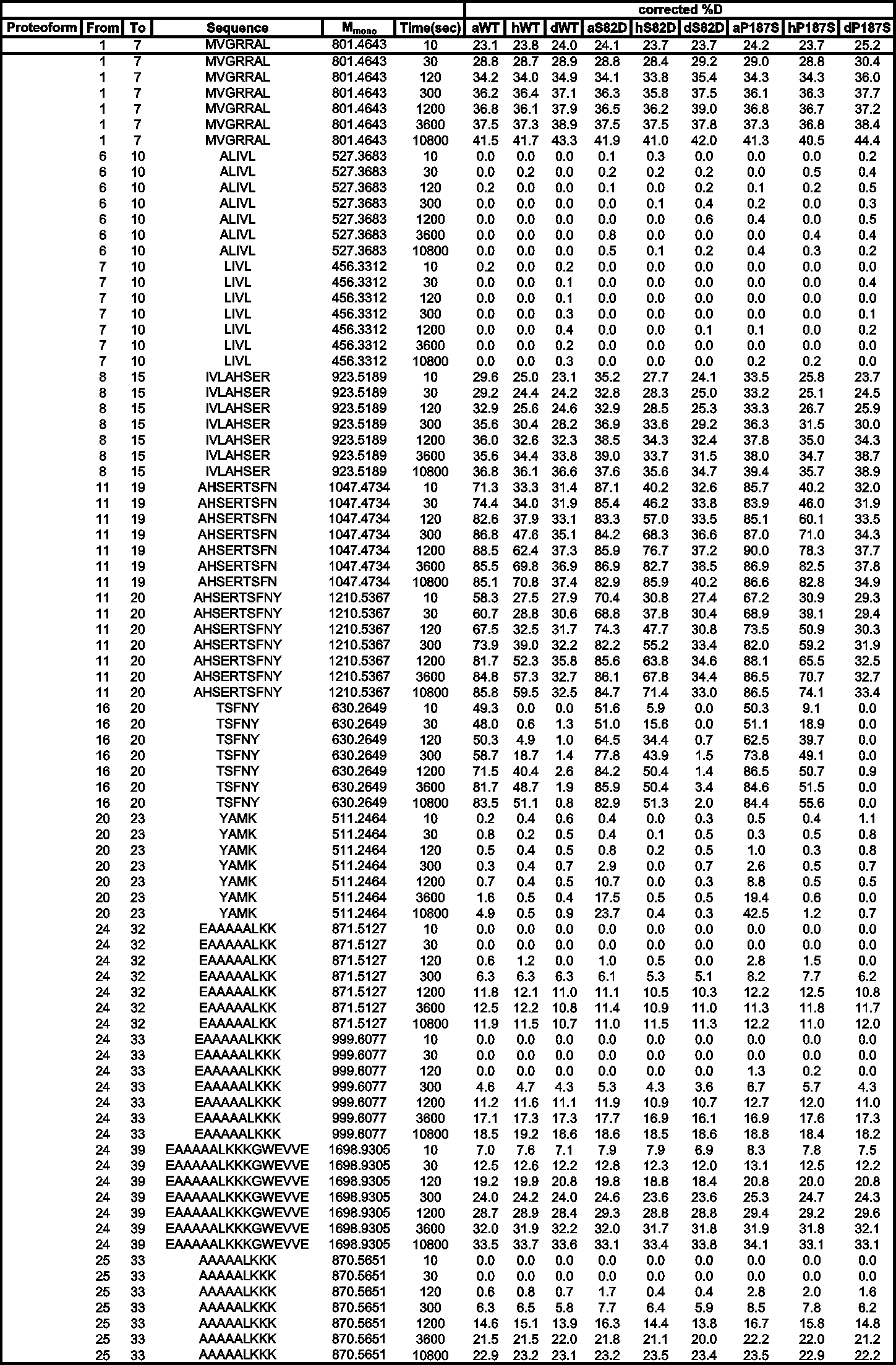
**

**
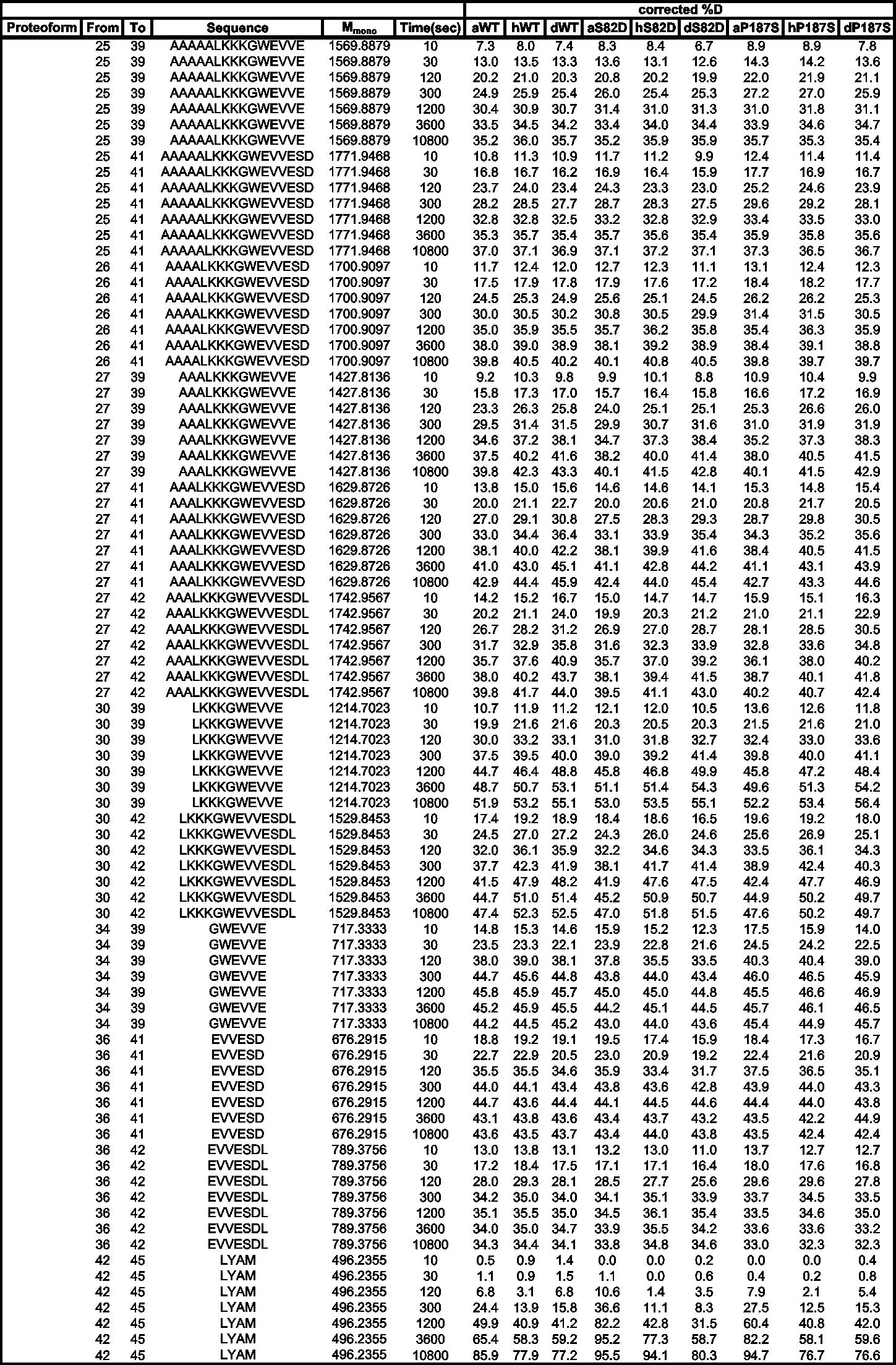
**

**
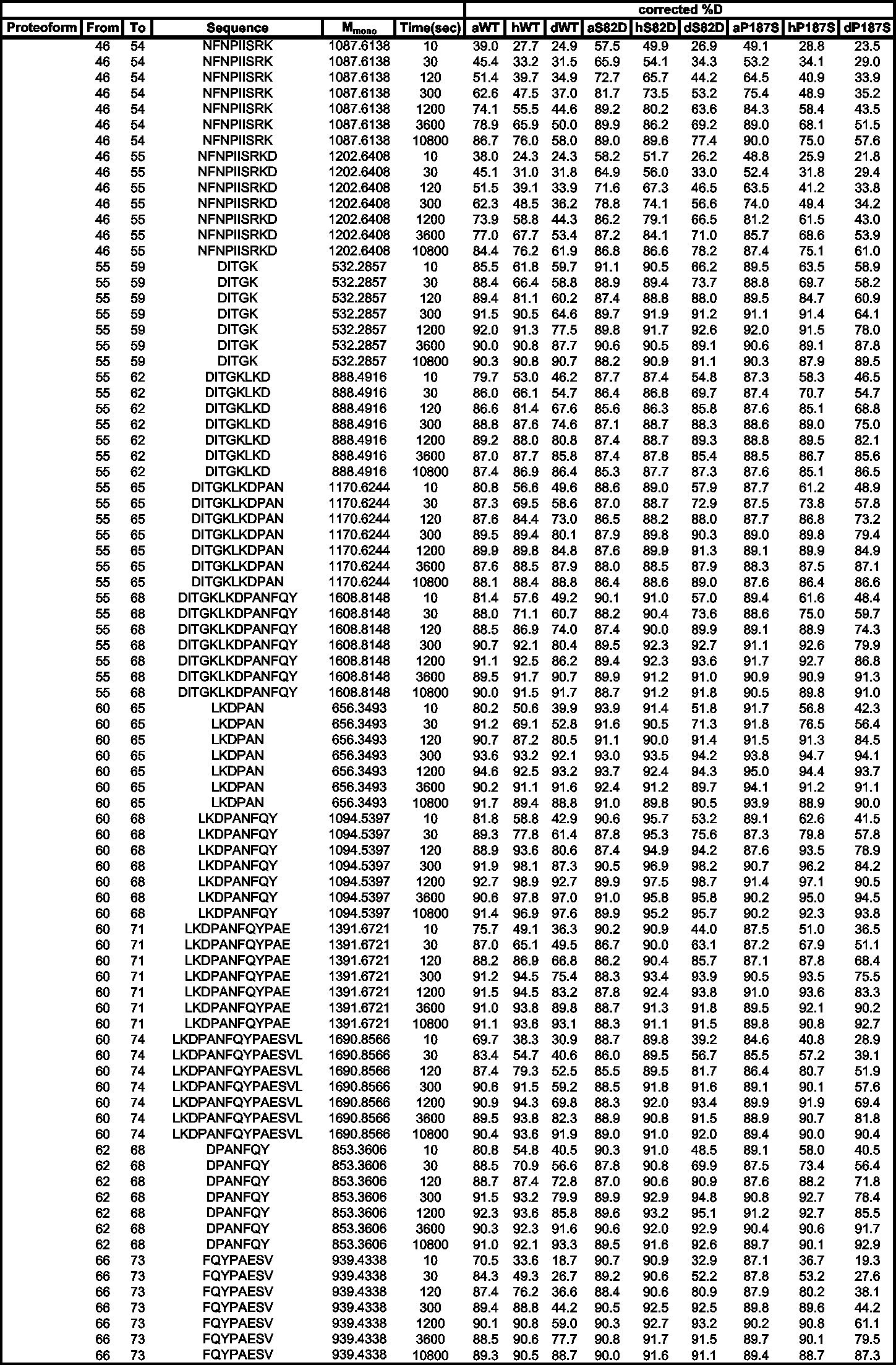
**

**
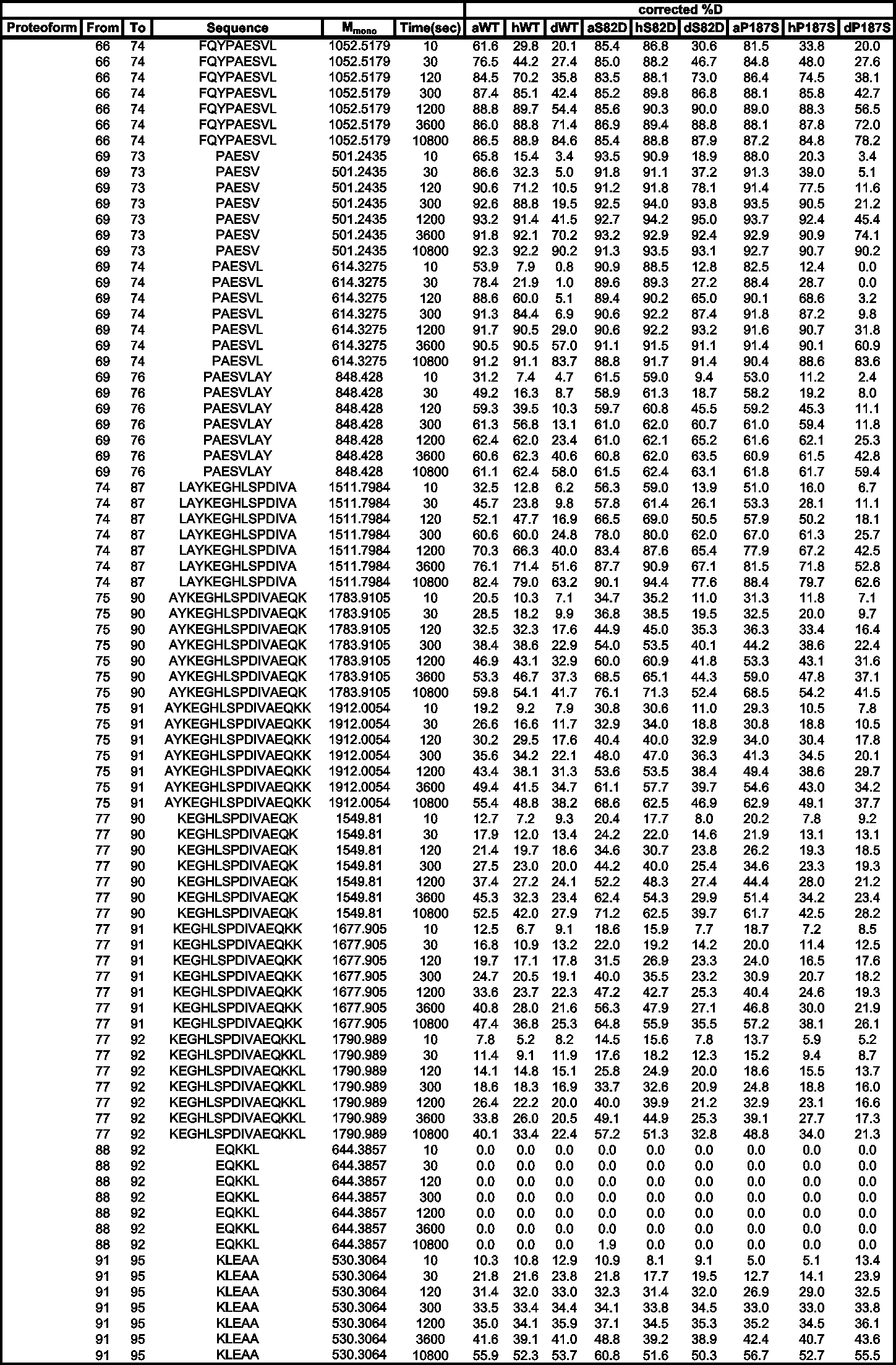
**

**
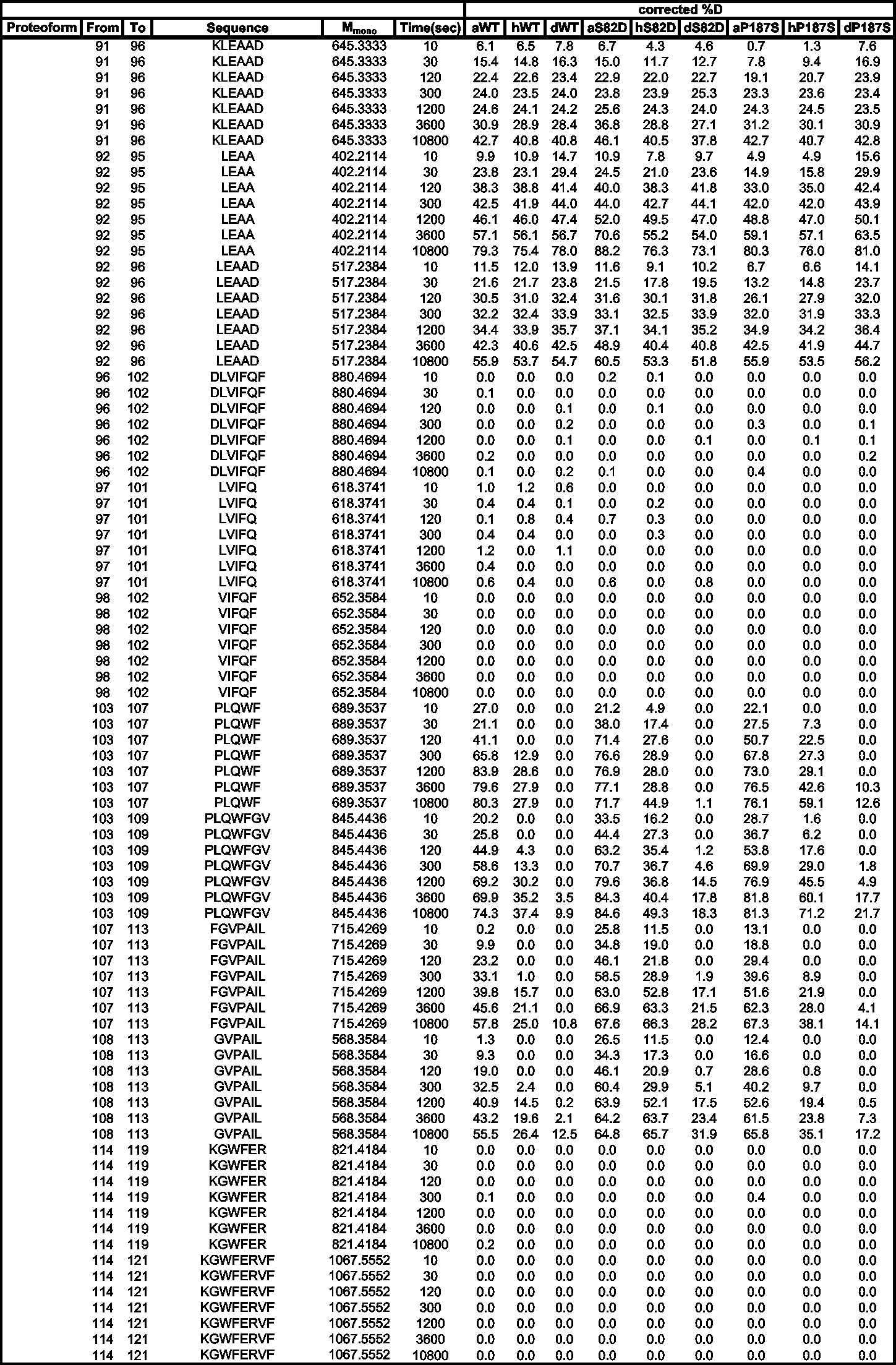
**

**
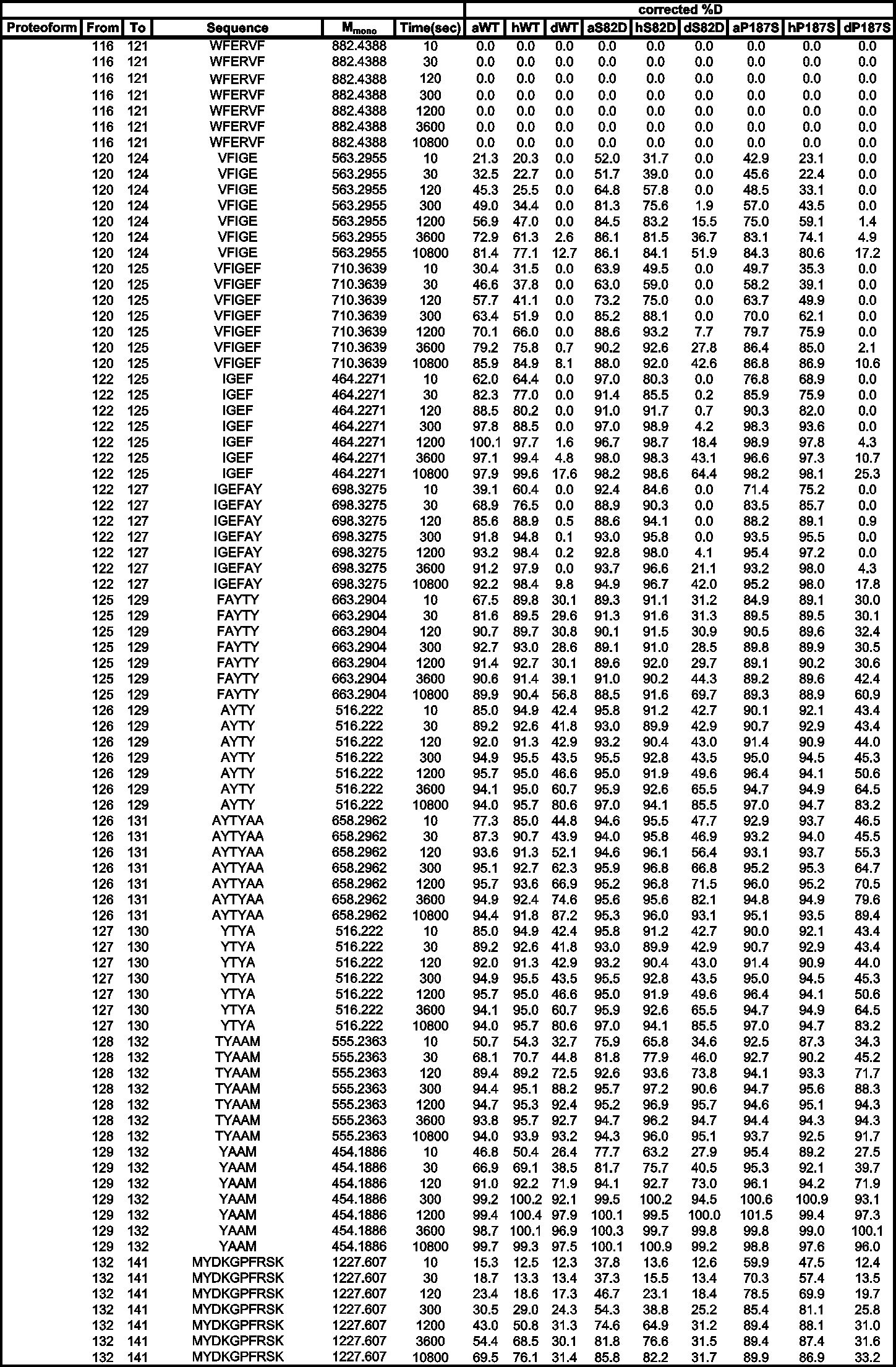
**

**
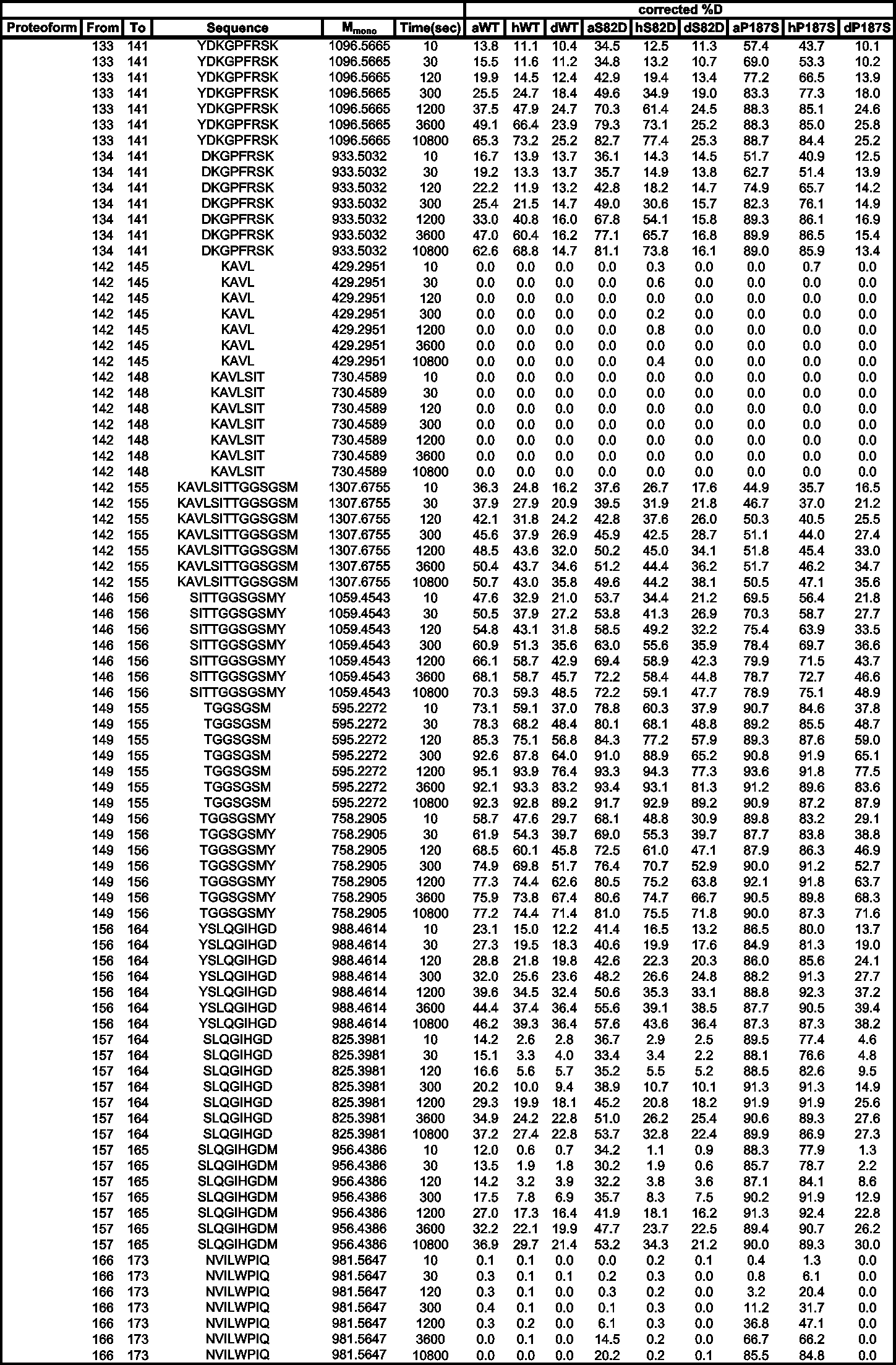
**

**
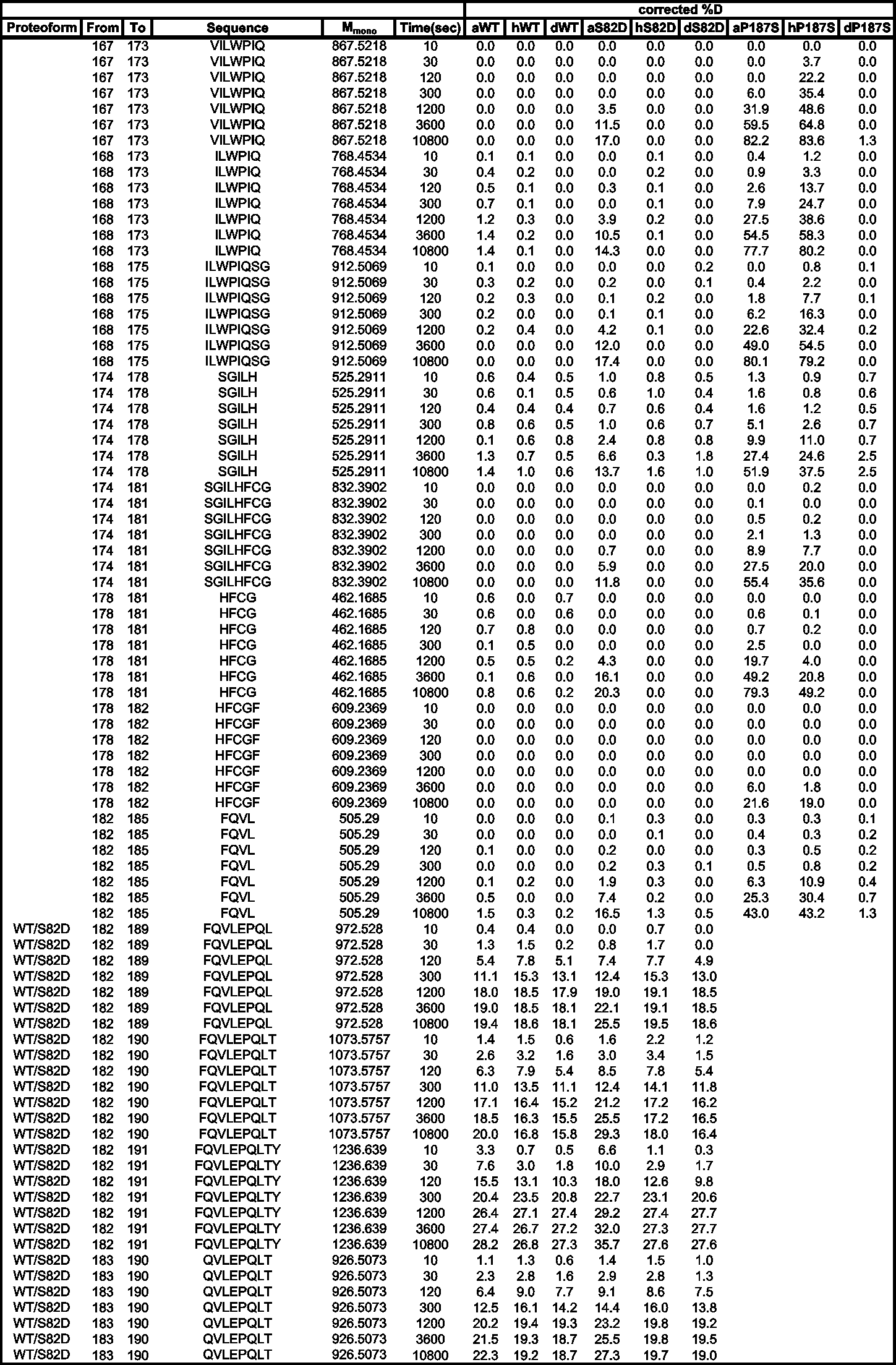
**

**
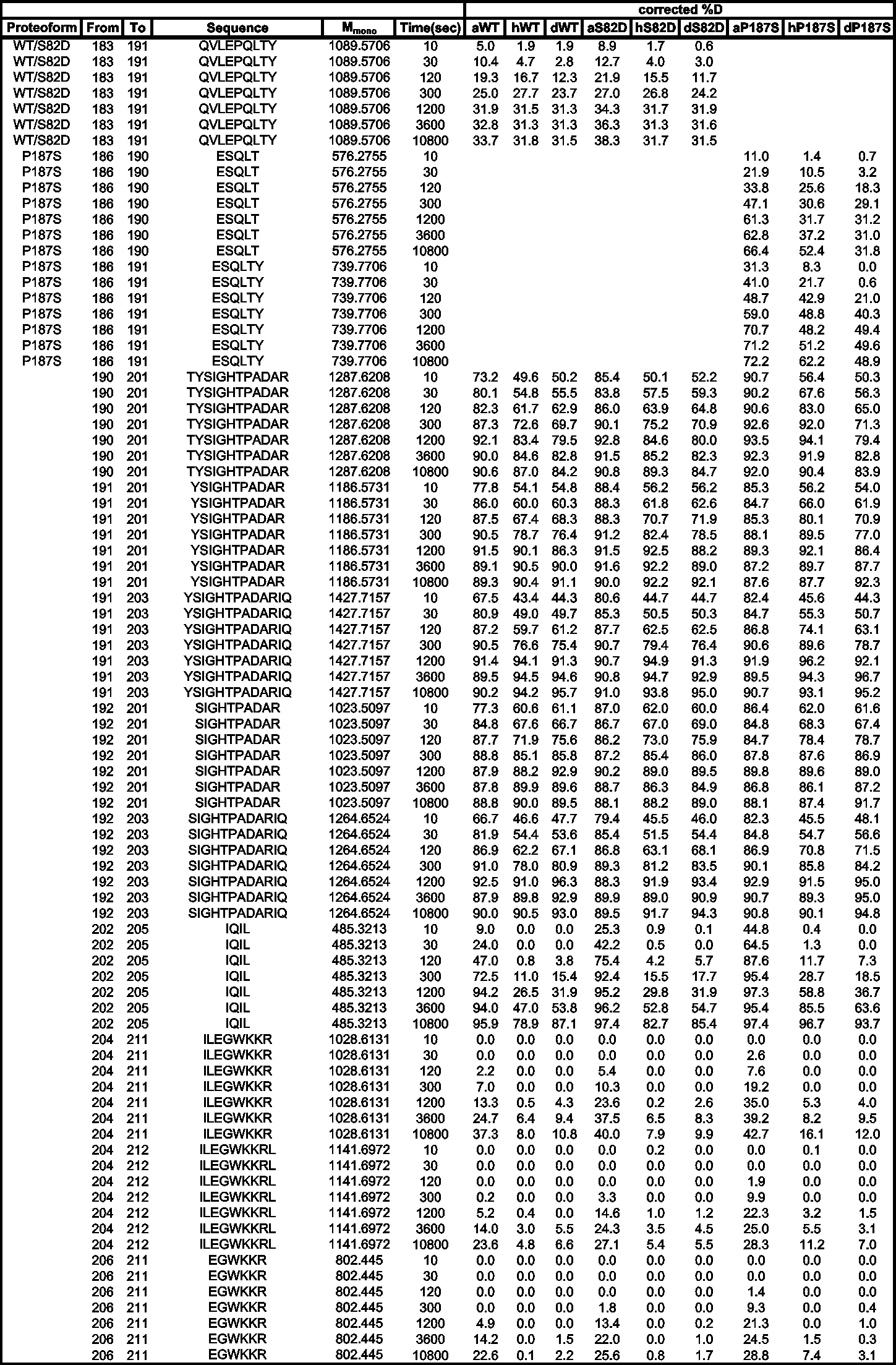
**

**
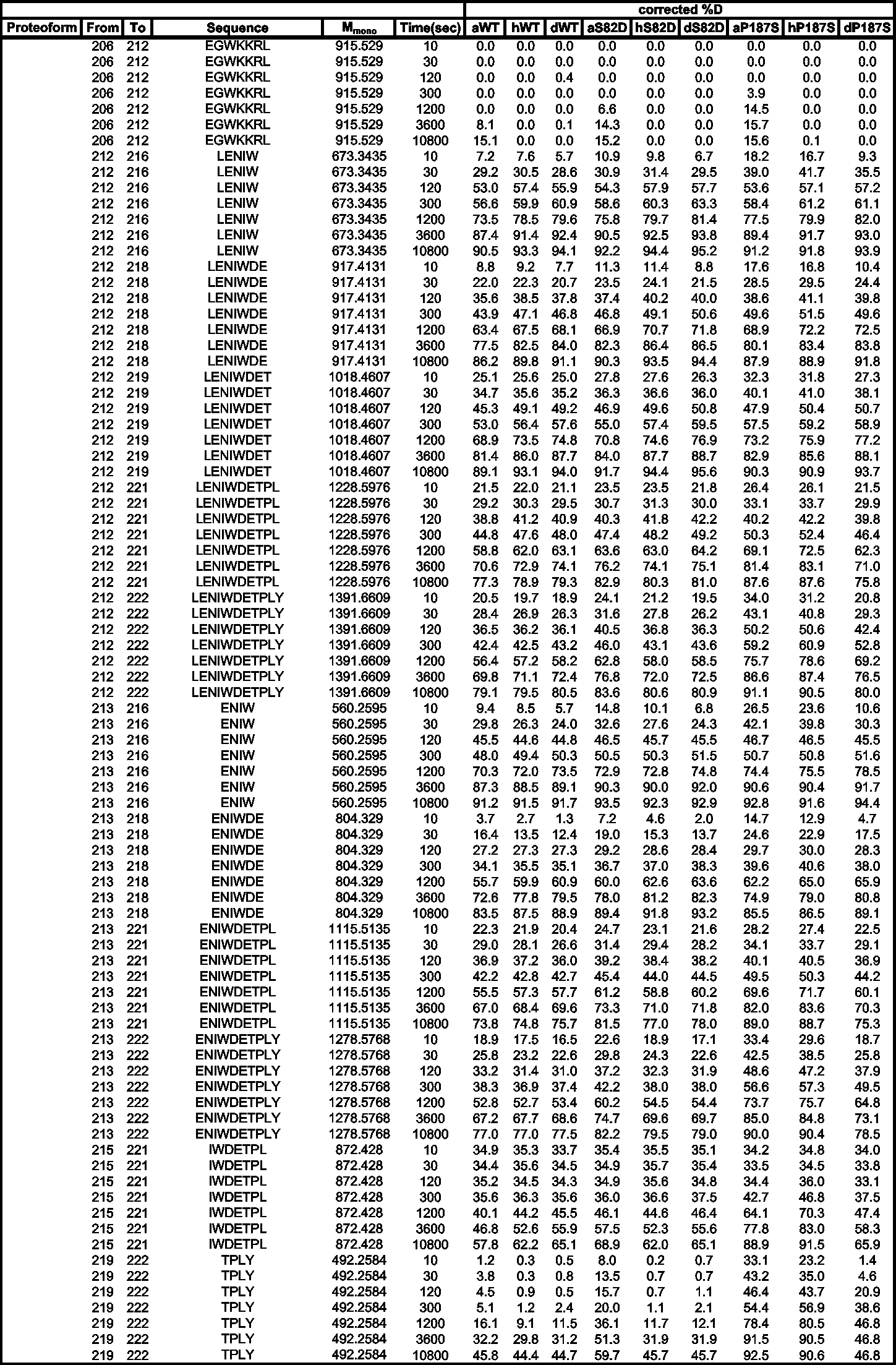
**

**
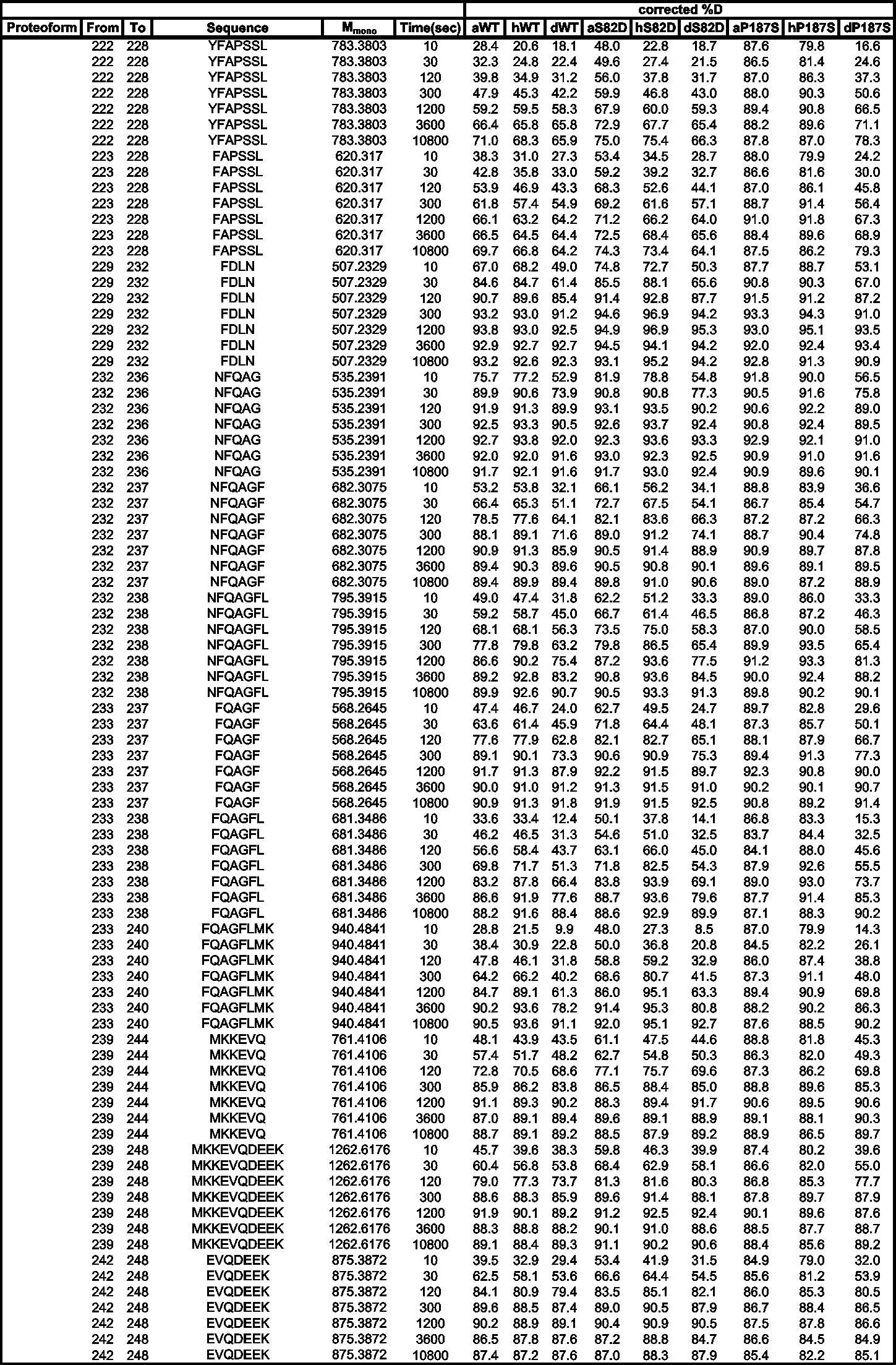
**

**
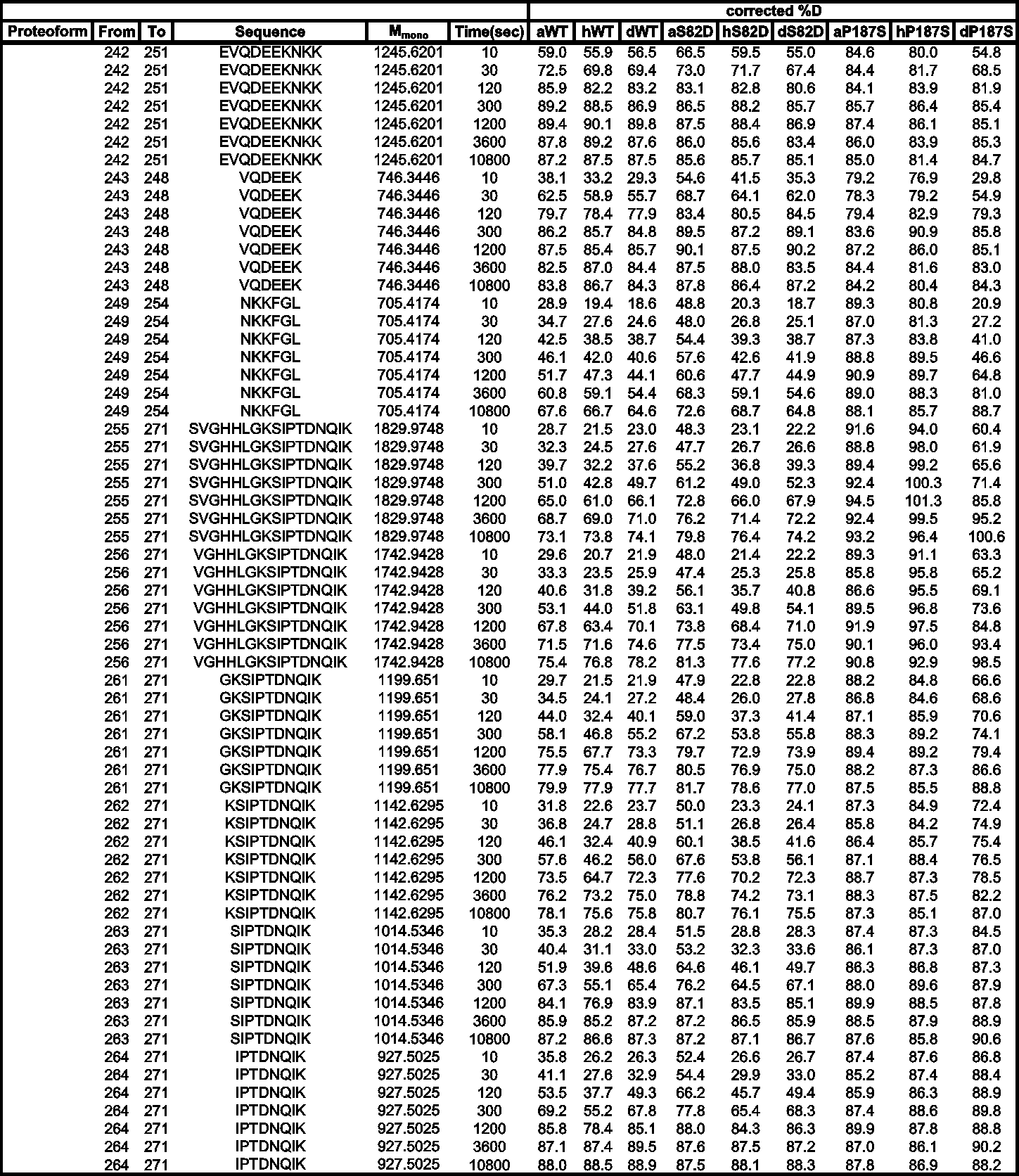
**

**Table S5. Parameters for the fittings to hyperbolic functions for the reduction of FAD by NADH.** Experiments were carried out at 6^o^C**.** Data for WT are from [[7](#_ENREF_7)].

|  | **Fast FAD reduction**  **(A→B)** | | | **Slow FAD reduction**  **(B→C)** | | |
| --- | --- | --- | --- | --- | --- | --- |
|  | ***k*_HT1_**  **(s^-1^)** | ***K*_d1_^NADH^ (μM)** | ***k*_HT1_/*K*_d1_^NADH^ (μM^-1^·s^-1^)** | ***k*_HT2_**  **(s^-1^)** | ***K*_d2_^NADH^ (μM)** | ***k*_HT2_/*K*_d2_^NADH^ (μM^-1^·s^-1^)** |
| **WT** | 281±14 | 15±2 | 19±3 | 14±2 | 8.2±3.6 | 1.9±1.1 |
| **S82D** | 368±33 | 23±5 | 16±5 | 9.8±0.9 | 43±8 | 0.23±0.06 |
| **P187S** | 480±79 | 59±18 | 8.1±3.8 | 1.8±0.5 | 75±34 | 0.024±0.06 |

**Table S6. Observed rate constants for the anaerobic reoxidation of NQO1_hq_ variants (i.e. NQO1_holo_ reduced with NADH) by equimolecular concentrations of DCPIP.** Data are mean ± SD from n > 3. Experiments carried out at 6^o^C. Data for WT are from [[7](#_ENREF_7)].

| **Variant** | **NQO1_hq_ reoxidation by DCPIP** | |
| --- | --- | --- |
|  | $\boldsymbol{k}_{\boldsymbol{obs}\boldsymbol{1}}$  **(s^-1^)** | $\boldsymbol{k}_{\boldsymbol{obs}\boldsymbol{2}}$  **(s^-1^)** |
| **WT** | > 500 | 160 ± 14 |
| **S82D** | 277 ± 18 | 72 ± 9 |
| **P187S** | 226 ± 14 | 32 ± 4 |

**Table S7. KIEs and Arrhenius parameters for the HT in the reduction of NQO1 variants by NADH.** All values correspond to data obtained with equimolecular concentrations of the reactants in the stopped-flow equipment. (n > 3, mean ± SD). Analysis were performed as previously described [[7](#_ENREF_7)].

**Parameters for the fast FAD reduction**

|  | **By NADH** | | |  | **By NADD** | | |  | **KIE** | **ΔE_aD1_-_H1_**  **(kcal·mol^-1^)** | **A_H1_/A_D1_** |
| --- | --- | --- | --- | --- | --- | --- | --- | --- | --- | --- | --- |
|  | $\boldsymbol{k}_{\boldsymbol{obs}\boldsymbol{1}}^{\boldsymbol{a}}$  **(s^-1^)** | **E_aH1_**  **(kcal·mol^-1^)** | **A_H1_**  **(s^-1^)** |  | $\boldsymbol{k}_{\boldsymbol{obs}\boldsymbol{1}}^{\boldsymbol{a}}$  **(s^-1^)** | **E_aD1_**  **(kcal·mol^-1^)** | **A_D1_**  **(s^-1^)** |  |  |  |  |
| **WT** ^b^ | 78±1 | 6.1±0.2 | (5.3±1.2)  ·10^6^ |  | 44±2 | 6.3 ± 0.4 | (4.1±1.1)  ·10^6^ |  | 1.8±0.1 | 0.2±0.4 | 1.3±0.6 |
| **S82D** | 68±3 | 4.9±0.1 | (4.9±1.0)  ·10^5^ |  | 41±1 | 5.4±0.2 | (6.4±3.3)  ·10^5^ |  | 1.7±0.1 | 0.5±0.2 | 0.8±0.4 |
| **P187S** | 90±1 | 4.4±0.1 | (2.7±0.8)  ·10^5^ |  | 46±1 | 4.2±0.4 | (9.6±9.0)  ·10^4^ |  | 2.0±0.1 | -0.2±0.4 | 2.8±1.6 |

**Parameters for the slow FAD reduction**

|  | **By NADH** | | |  | **By NADD** | | |  | **KIE** | **ΔE_aD2-H2_ (kcal**  **·mol^-1^)** | **A_H2_/A_D2_** |
| --- | --- | --- | --- | --- | --- | --- | --- | --- | --- | --- | --- |
|  | $\boldsymbol{k}_{\boldsymbol{obs}\boldsymbol{2}}^{\boldsymbol{a}}$  **(s^-1^)** | **E_aH2_**  **(kcal**  **·mol^-1^)** | **A_H2_**  **(s^-1^)** |  | $\boldsymbol{k}_{\boldsymbol{obs}\boldsymbol{2}}^{\boldsymbol{a}}$  **(s^-1^)** | **E_aD2_**  **(kcal**  **·mol^-1^)** | **A_D2_**  **(s^-1^)** |  |  |  |  |
| **WT** ^b^ | 8.9±0.9 | 10.9±0.5 | (3.4±0.9) ·10^9^ |  | 5.3±0.2 | 9.8±0.5 | (2.6±0.6) ·10^8^ |  | 1.7±0.2 | -1.1±0.7 | 13±6 |
| **S82D** | 2.0±0.8 | 13.0±0.1 | (3.0±0.8) ·10^10^ |  | 1.4±0.3 | 17.7±0.3 | (1.0±0.5) ·10^14^ |  | 1.4±0.6 | 4.7±0.3 | (3±2) ·10^-4^ |
| **P187S** | 0.13±0.01 | 4.8±0.2 | (7.6±3.1) ·10^2^ |  | 0.13±0.01 | 4.7±0.2 | (6.3±2.0) ·10^2^ |  | 1.0±0.1 | -0.1±0.3 | 1.2±0.6 |

^a^Values at 6 °C. ^b^Data from [[7](#_ENREF_7)].

**Table S8. Thermodynamic stability of the CTD inferred from proteolysis kinetics.**

|  | **NQO1_apo_** | | **NQO1_holo_** | | **NQO1_dic_** | |
| --- | --- | --- | --- | --- | --- | --- |
|  | *k*_prot_*  (M^-1^·min^-1^) | ΔΔG_prot_** (kcal·mol^-1^) | *k*_prot_  (M^-1^·min^-1^) | ΔΔG_prot_ (kcal·mol^-1^) | *k*_prot_  (M^-1^·min^-1^) | ΔΔG_prot_ (kcal·mol^-1^) |
| **WT** | 4.07±0.24  ·10^6^ | 0** | 3.28±0.10  ·10^4^ | 2.86±0.04 | 1.84±0.19  ·10^4^ | 3.20±0.07 |
| **S82D** | 2.69±0.20  ·10^6^ | 0.25±0.06 | 5.30±0.27  ·10^5^ | 1.21±0.05 | 5.33±0.37  ·10^4^ | 2.57±0.03 |
| **P187S** | 1.05±0.12  ·10^7^ | -0.56±0.08 | 1.20±0.24  ·10^7^ | -0.64±0.12 | 4.53±0.49  ·10^5^ | 1.30±0.07 |

* Second-order rate constants for proteolysis with trypsin.

** Change in unfolding free energy using WT-NQO1_apo_ as reference.

**Table S9. Apparent binding thermodynamic parameters for the interaction between NQO1 and dicoumarol.**

|  | N* | K_d_ **  (nM) | ΔG**  (kcal·mol^-1^) | ΔH**  (kcal·mol^-1^) | -TΔS**  (kcal·mol^-1^) | ΔC_p_***  (kcal·mol^-1^·K^-1^) |
| --- | --- | --- | --- | --- | --- | --- |
| WT | 1.05±0.04 | 18±11 | -10.5±0.3 | -8.7±0.3 | -1.8±0.4 | -0.37±0.04 |
| S82D | 1.14±0.06 | 91±36 | -9.6±0.2 | -12.0±0.8 | 2.4±0.9 | -0.59±0.07 |
| P187S | 0.90±0.09 | 560±270 | -8.4±0.3 | -31.7±4.8 | 23.3±4.7 | -2.02±0.23 |

* Mean±s.d. from at least five experiments at different temperatures.

** Mean±s.d. from at least three experiments at 25^o^C.

*** Slope from a linear fitting of the binding ΔH vs. Temperature.

**References**

[1] J. Schymkowitz, J. Borg, F. Stricher, R. Nys, F. Rousseau, L. Serrano, The FoldX web server: an online force field, Nucleic Acids Res, 33 (2005) W382-388.

[2] R. Fraczkiewicz, W. Braun, Exact and Efficient Analytical Calculation of the Accessible Surface Areas and Their Gradients for Macromolecules, J.Comp.Chem., 19 (1998) 319-333.

[3] H. Wickham, ggplot2: Elegant Graphics for Data Analysis. , Springer-Verlag, 2016.

[4] A. Martinez-Limon, M. Alriquet, W.H. Lang, G. Calloni, I. Wittig, R.M. Vabulas, Recognition of enzymes lacking bound cofactor by protein quality control, Proc Natl Acad Sci U S A, 113 (2016) 12156-12161.

[5] N. Tokuriki, F. Stricher, J. Schymkowitz, L. Serrano, D.S. Tawfik, The stability effects of protein mutations appear to be universally distributed, J Mol Biol, 369 (2007) 1318-1332.

[6] P. Vankova, E. Salido, D.J. Timson, P. Man, A.L. Pey, A dynamic core in human NQO1 controls the functional and stability effects of ligand binding and their communication across the enzyme dimer, Biomolecules, 9 (2019) 728.

[7] E. Anoz-Carbonell, D.J. Timson, A.L. Pey, M. Medina, The Catalytic Cycle of the Antioxidant and Cancer-Associated Human NQO1 Enzyme: Hydride Transfer, Conformational Dynamics and Functional Cooperativity, Antioxidants (Basel), 9 (2020).
